# Optical Study of Solvatochromic Isocyanoaminoanthracene Dyes and 1,5-Diaminoanthracene

**DOI:** 10.3390/ijms23031315

**Published:** 2022-01-24

**Authors:** Miklós Nagy, Béla Fiser, Milán Szőri, László Vanyorek, Béla Viskolcz

**Affiliations:** Institute of Chemistry, University of Miskolc, Miskolc-Egyetemváros, 3515 Miskolc, Hungary; kemfiser@uni-miskolc.hu (B.F.); kemvanyi@uni-miskolc.hu (L.V.); bela.viskolcz@uni-miskolc.hu (B.V.)

**Keywords:** anthracene, fluorescence, solvatochromic effect, isonitrile, DFT

## Abstract

Isocyanoaminoarenes (ICAAr-s) are a novel and versatile group of solvatochromic fluorophores. Despite their versatile applicability, such as antifungals, cancer drugs and analytical probes, they still represent a mostly unchartered territory among intramolecular charge-transfer (ICT) dyes. The current paper describes the preparation and detailed optical study of novel 1-isocyano-5-aminoanthrace (ICAA) and its N-methylated derivatives along with the starting 1,5-diaminoanthracene. The conversion of one of the amino groups of the diamine into an isocyano group significantly increased the polar character of the dyes, which resulted in a significant 50–70 nm (2077–2609 cm^−1^) redshift of the emission maximum and a broadened solvatochromic range. The fluorescence quantum yield of ICAAs is strongly influenced by the polarity of the solvent. The starting anthracene-diamine is highly fluorescent in every solvent (√_f_ = 12–53%), while the isocyano derivatives are practically nonfluorescent in solvents more polar than dioxane. This phenomenon implies the potential application of ICAAs to probe the polarity of the medium and is favorable in practical applications, such as cell-staining, resulting in a reduced background fluorescence. The ICT character of the emission states of ICAAs are in good agreement with the computational findings presented in TD-DFT calculations and molecular electrostatic potential (MESP) isosurfaces.

## 1. Introduction

Polycyclic aromatic hydrocarbon (PAH) moieties such as pyrene and anthracene are important building blocks of smart electronic and fluorescent materials [1,2,3]. Owing to their rigid planar structure and easy substitutability with reactive or bulky functional groups, they can be incorporated into more complex structures, such as graphene nanoribbons [4] and ribbon-like pyrene-fused pyrazaacenes (PPAs) [5]. The unprecedented optoelectronic properties of these complex structures can be utilized in many optoelectronic applications, for example, in dye sensitized solar cells (DSSCs) [6] among all. In addition, anthracene moiety makes up the core of many important fluorescent probes used for the detection of transition metal ions such as Zn^2+^ [7]. The formation of at least two amino groups on PAHs in symmetric positions offers an easy way to incorporate the aromatic moiety into more complex structures through alkylation, acylation or diazotation reactions. In addition, by varying the number and position of N atoms and the substitution on the aromatic core along the π-framework, it is possible to modulate the electronic structure, stability, solubility and supramolecular organization [8,9]. In this context, the molecular organization in π-conjugated systems could be further controlled by virtue of the cooperative effect of stronger non-covalent interactions. Among them, hydrogen bonding represents an appropriate tool [10,11,12], as it is evidenced by biological systems in which the combination of π-stacking and hydrogen bonds determines the macrostructure of proteins or nucleic acids, just to mention well-known examples. One of the most important members of diamino PAHs is 1,5-diaminoanthracene, which is used to construct organic semiconductors [13], dinuclear nickel complexes for highly active ethylene dimerization [14], self-assembled small-molecule-based hole-transporting material for inverted perovskite solar cells [15], photoactivated healable vitrimeric copolymers [16], amorphous porous organic polymers for highly efficient iodine capture [17], bimetallic aluminum complexes for ring-opening polymerization of lactide [18], Luminescent Supramolecular Lanthanide Complexes [19], Squaraine dyes [20] and others. Despite its widespread application, the literature on the optical properties of 1,5-diaminoanthracene is very limited.

The optical properties may be further enhanced (while preserving the important H-bonding ability) by converting one of the amino groups into isocyanide using dichlorocarbene [21]. Only a few members of the resulting isocyanoaminoarene substance family have been prepared and studied until recently, despite their simple structure and preparation. Isocyanoaminoarenes are typically built up from an electron-donating (amino, D) and an electron-withdrawing (isocyano, A) group, connected through an aromatic π-linker moiety. These so-called D-π-A type solvatochromic fluorophores are therefore based on the shift of the electron density from the donor group to the acceptor moiety through the π-system upon excitation; hence, an intramolecular charge-transfer (ICT) takes place [22,23], which may result in an increase in the excited state dipole moment with respect to that of the ground state. It is believed that the presence of ICT, in the absence of any specific interaction, i.e., hydrogen-bonding, between the fluorophore and the solvent is the primary reason of solvatochromism. Fluorophores, whose fluorescence emissions are particularly sensitive to the polarity of their microenvironment and hence they alter the emitted light color upon the effect of polarity change, are called solvatochromic fluorophores [23].

Recently, we have synthesized a series of new solvatochromic fluorophores based on the preliminary concept of ICT in which the donor amine and the acceptor isocyano groups are connected via the naphthalene moiety in its 1,5-positions to yield 1,5-isocyanoamino- (1,5-ICAN) derivatives [21,24,25,26]. The 1,5-ICANs exhibit large solvatochromic and Stokes shifts and turned out to be one of the most versatile “smart” fluorophore dye families. They found application as nontoxic supravital stains for the investigation and characterization of both plant and animal cells [27,28], enable the selective detection of Hg^2+^ and, at the same time, can indicate the presence of Ag^+^, which is unprecedented among fluorescent sensors [29]. ICANs can also be utilized in silver analytics as isocyanide ligands [26] and the simultaneous presence of the amino, isocyano and naphthalene groups yielded a most effective antifungal drug, the efficacy of which was demonstrated in vivo in mice against *Candida* strains [30]. Moreover, the small modification of acridine orange (the aromatic core is very similar to the anthracene in this study) resulted in a very efficient physiological pH-probe [31] and the new isocyano-aminoacridines opened up a new pathway in cancer treatment based on phototoxicity studies [32].

The rational design and development of more efficient solvatochromic fluorophores requires a deeper understanding of their structure–property relationships. One of the key factors is the aromatic core of the D-π-A system, which determines the distance between the donor and acceptor groups, and therefore also the dipole moment (and its change upon excitation) of the molecule. We assume that the solvatochromic range of ICANs could be extended while keeping their advantageous properties by exchanging the naphthalene ring to the larger anthracene. Since the field of isocyanoaminoarenes is still largely unexplored, we believe that they may contain a considerable potential to become versatile smart dyes. The aim of this study is therefore the preparation of novel 1-amino-5-isocyanoanthracenes and the detailed optical study of these fluorophores along with the starting 1,5-diaminoanthracene.

## 2. Results and Discussion

**Figure 1:**
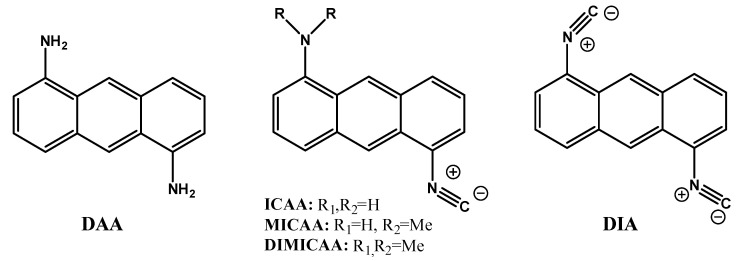
The structure and name of the dyes used in this study. The full names are given in the experimental section.

1,5-Diaminoanthracene (**DAA**) was obtained by the reduction of 1,5-Diaminoanthraquinone and was easily converted to 1-Amino-5-isocyanoanthracene (**ICAA**) and 1,5-Diisocyano-anthracene (**DIA**) by the reaction of the amino group(s) with dichlorocarbene. To enhance the optical properties, alkylation of **ICAA** was carried out by using MeI on the free amino group to yield (**MICAA**) and (**DIMICAA**)**.** The structures of the dyes in this study are presented in Figure 1. The purity and structure of the compounds were checked by ^1^H- and ^13^C-NMR (spectra can be found in the Supporting Information). ESI-MS measurements further confirmed the structures as the measured m/z values differed with no more than ±0.01 Da from those of the ones calculated.

### 2.1. UV–Vis Electronic Absorption Properties of 1-Amino-5-Isocyanoanthracene Derivatives

To obtain a deeper understanding of the ground state electronic properties of the starting 1,5-diaminoanthracene and its isocyano derivatives, UV–vis spectra were recorded in 15 different solvents, selected to cover a broad range of solvent polarity, spanning from the non-polar hexane to the polar H_2_O. Another criterion for solvent selection was their ability to form H-bond, either as donor or acceptor, to study the specific solute–solvent interactions, too. All the UV–vis, steady-state emission and excitation spectra in various solvents are presented in the Supporting Information (SI) for all the substances as separate chapters.

**Figure 2:**
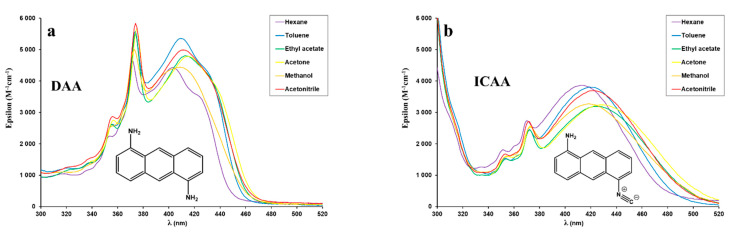

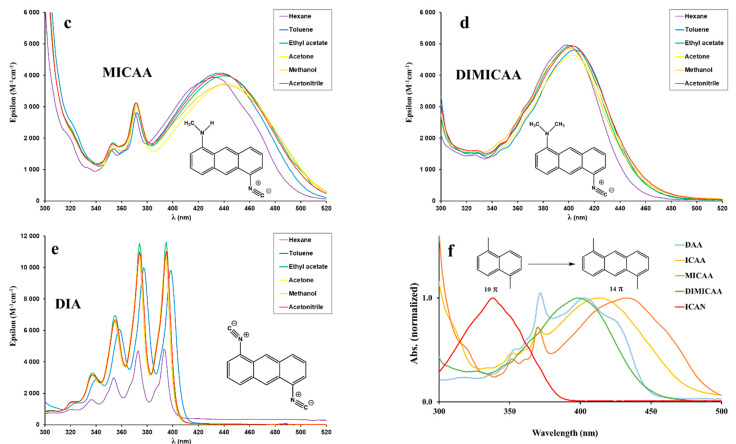
UV–vis spectra of 1,5-diaminoanthracene and the isocyano derivatives recorded in solvents of different polarity (**a**–**e**). The normalized UV–vis spectra (**f**) of DAA-DIMICAA in hexane in comparison to 1-amino-5-isocyanonaphthalene (**ICAN**). ([dye] = 5 × 10^−5^ M, T = 20 °C).

The UV–vis spectra of all the dyes in this study in different solvents are shown in Figure 2, while the absorption maximum wavelength (λ_Abs_) and the corresponding molar extinction coefficients (ε) are compiled in Table 1.

As it is evident from Figure 2f and the data of Table 1, the exchange of the 10 π-electron naphthalene ring of ICAN to the 14 π-electron anthracene ring in ICAA, MICAA and DIMICAA results in a significant bathochromic shift (from 338 nm of ICAN to 412 nm of ICAA) of the absorption maximum. This >70 nm (5315 cm^−1^) redshift is favorable for the practical applicability of the dyes (i.e., cell-staining), since they can be excited using blue-light instead of UV-light, which is toxic for living cells. Independently of solvent polarity, similarly for DAA-DIMICAA, a broad long wavelength absorption band is seen in the range of ~350–500 nm (or ~28571–20000 cm^−1^, Figure 2f), which can be attributed to the intramolecular charge-transfer transition (ICT) between the donor amino and the acceptor isocyano groups. The ICT character of this band is further supported by the fact that the absorption spectrum of the diisocyano derivative (DIA), where both substituents are electron withdrawing, is almost identical to that of the unsubstituted anthracene [33]. In addition, the absorption bands of the 1,5-diaminoanthracene (DAA, Figure 2a, Figures S2–S16) are narrower and more structured than those of the amino-isocyano derivatives (ICAA-DIMICAA). The ICT band is accompanied by an overlapping sharp absorption band around 370 nm (27027 cm^−1^) in DAA, ICAA and MICAA, belonging either to the locally excited state (LE) of the aromatic anthracene ring or may appear owing to the vibrational progression associated with N-H bonding. Since this sharp band is completely absent in the case of the dimethylamino-derivative (DIMICAA), which does not have any N-H bond, the N-H vibrational origin is more plausible, as we have shown previously in the case of our ICAN derivatives [24].

It is clearly seen from the data of Table 1 that λ_Abs_ is dependent upon the solvent polarity, i.e., the ICT absorption bands suffer a slight redshift with increasing solvent polarity. The bathochromic shifts (from n-hexane to DMSO) are approximately 20 nm (1340 cm^−1^) for DAA and MICAA and 30 nm (1750 cm^−1^) for ICAA, while surprisingly only 10 nm (739 cm^−1^) for DIMICAA. The shifts of the low energy bands are indicative of the polar character of the ground state. Indeed, DFT calculations yielded 2.0, 6.0, 5.5 and 5.5 D ground state dipole moments in hexane (Table 2.) for the 1,5-diamino (DAA), 1-amino-5-isocyano (ICAA), 1-*N*-methylamino-5-isocyano (MICAA) and 1-*N,N*-dimethylamino-5-isocyano (DIMICAA) derivatives, respectively. It should be noted, however, that the symmetric product: 1,5-diisocyanoanthracene does not have a dipole moment in either ground or excited state, which explains the lack of ICT band and the structured anthracene-like absorption spectrum.

It is evident from the data of Table 2 that the conversion of one of the amino groups of DAA into isocyano group significantly increased the polar character (larger dipole moments) of the dyes. However, it can also be surmised that besides dipole moments, specific dye–solvent interactions, such as H-bonds, may also be responsible for the position of λ_Abs_. In H-bond acceptor solvents, such as THF, dioxane and pyridine, λ_Abs_ values are significantly higher than would be expected based on the dielectric constants (polarity) of the solvents. For example, in the case of MICAA, λ_Abs_ = 451 nm in pyridine, the highest value is the same as in DMSO. However, the dielectric constant of pyridine is only ε_r_ = 12.4, while that of DMSO is ε_r_ = 46.7. In contrast, in H-bond donor solvents, such as isopropanol, methanol and chloroform, λ_Abs_ values are significantly lower than would be expected based on polarity of the solvent. In DIMICAA, where the amino group is dimethylated and there is no N-H bond, the possibility of the formation of H-bonds is limited, which can explain its narrower λ_Abs_ range in different solvents compared to those of DAA-MICAA, where at least 1 N-H bond is present.

Interestingly, λ_Abs_ values in water, as in the most polar compound listed in Table 1, are lower than those measured in DMSO for all the amino-isocyano derivatives (ICAA-DIMICAA) and for the starting diamine (DAA), too. The wavelength differences (λ_Abs_, _DMSO_–λ_Abs_, _H2O_) are 31 nm (1842 cm^−1^), 34 nm (1868 cm^−1^), 36 nm (1923 cm^−1^) for the 1,5-DAA, 1,5-ICAA and 1,5-MICAA, respectively, while only 10 nm (550 cm^−1^) for 1,5-DIMICAA. The solvatochromic shifts in DMSO can be probably explained by hydrogen bonds. DMSO is a surprisingly good hydrogen bond acceptor and the large shifts observed for the compounds possessing NH groups are most probably the results of H-bond donation to DMSO molecules. This suggest that the solvatochromic response is strongly modulated by specific interactions, as well. Protic solvents, on the other hand, may form hydrogen bonds with the amine nitrogen. In this case, hypsochromic shifts are expected, since hydrogen bonding to the amine hydrogen would destabilize the ground state resulting in redshift, whereas hydrogen bonding to the amine nitrogen would stabilize the ground state resulting in blueshift. We previously showed the strong H-bond forming capability of amino-isocyanoarenes with pyridine [24]. Of course, the shifts are additionally influenced by the solvent polarity due to dielectric stabilization. 

### 2.2. Steady-State Fluorescence Properties 

**Figure 3:**
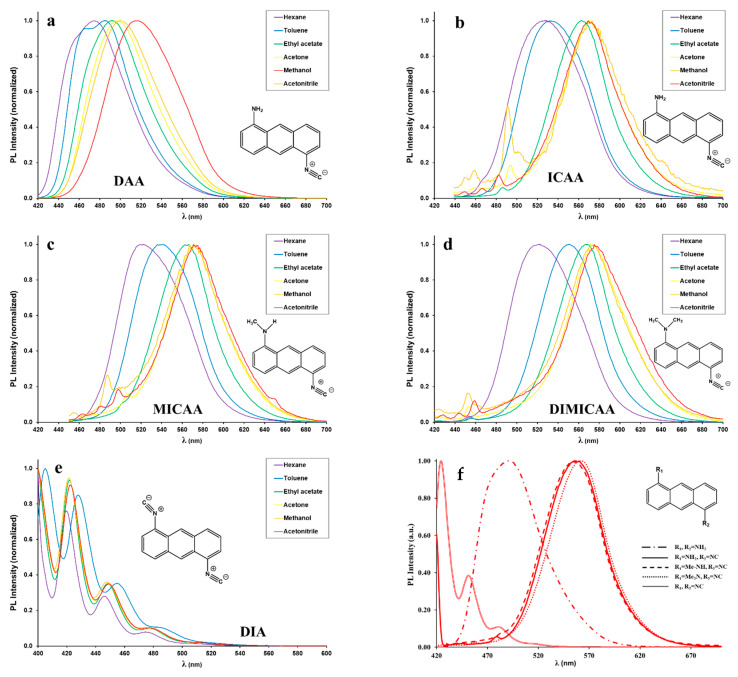
(**a**–**e**) Steady-state fluorescence spectra of DAA, ICAA, MICAA, DIMICAA and DIA recorded in various solvents of different polarity. (**f**) The normalized emission spectra of DAA-DIA recorded in dioxane. ([dye] = 5 × 10^−5^ M, T = 20 °C).

**Figure 4:**
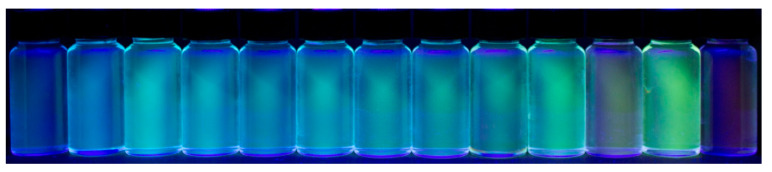

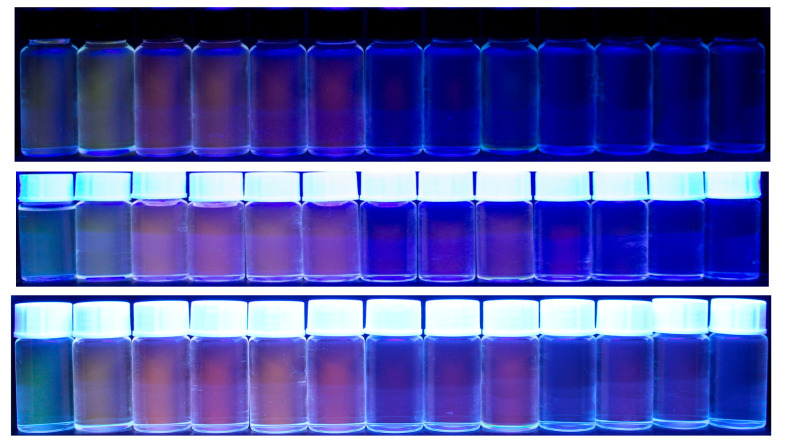
Demonstration of the fluorescence properties of 1,5-diaminoanthracene (DAA), 1-amino-5-isocyanoanthracene (ICAA), 1-*N*-methylamino-5-isocyanoanthracene (MICAA) and 1-*N,N*-dimethylamino-5-isocyanoanthracene (DIMICAA) (from top to bottom) in different solvents illuminated by light of λ = 365 nm. Solvents from left to the right are hexane, toluene, 1,4-dioxane, dichloromethane, chloroform, tetrahydrofuran (THF), acetonitrile, acetone, pyridine, methanol, dimethylformamide (DMF), dimethyl sulfoxide (DMSO), water.

Fluorescence spectra of all dyes (DAA-DIA) were recorded using λ_Abs_ values of Table 1 as the excitation wavelengths (λ_Ex_). The results are summarized in Figure 3 and Figure 4 (Figures S1, S33, S51, S69 and S72) and in Table 3.

As it is evident from Figure 3, the conversion of one amino group into isocyano resulted in a significant 50–70 nm (2077–2609 cm^−1^) redshift of the emission maximum of ICAA-DIMICAA compared to that of the starting diamine (DAA) in most of the solvents. ICAA, MICAA and DIMICAA (and to a limited extent DAA) clearly show positive solvatochromic behavior, i.e., their emission maximum shifts to higher wavelengths with increasing solvent polarity, due to the stabilizing effect of solvent reorganization around the excited dye [23]. The solvatochromic ranges (λ_em,DMSO_–λ_em,hexane_) are Δλ = 36 nm (1483 cm^−1^), 49 nm (1614 cm^−1^), 50 nm (1742 cm^−1^) and 56 nm (1863 cm^−1^) for DAA, ICAA, MICAA and DIMICAA, respectively. It is important to note that the diisocyano derivative DIA does not show any solvatochromic behavior because of the lack of ICT. When the fluorescence properties of ICAA are compared to those of our naphthalene-cored solvatochromic dye, ICAN, the bathochromic shifts are more significant due to the exchange of the 10π naphthalene core to the 14π anthracene core. For example, the emission maximum of ICAN was found to be at 409 and 497 nm for hexane and DMSO in the blue and greenish-blue region of visible light, while λ_em,max_ for ICAA is redshifted to 527 and 574 nm in the same solvents (Figure 3 and Figure 4). The bathochromic shift is almost 120 nm (5474 cm^−1^) in hexane; however, it is only 77 nm (2699 cm^−1^) in DMSO meaning the contraction of the emission range of ICAA to 49 nm (1553 cm^−1^), which is only half of that of ICAN. A possible explanation can be the limited solubility of ICAA in polar solvents, which is further backed by the observation that ICAA is practically nonfluorescent in solvents more polar than dioxane (Figure 4). Surprisingly, increasing the electron donating character of the amino group by methylation did not enhance the optical properties of ICAA as was shown previously for our ICAN compounds. Both the solvatochromic range and the position of the emission maximum are virtually the same for the methylated (MICAA), dimethylated (DIMICAA) and nonmethylated (ICAA) 1-amino-5-isocyanoanthracenes.

### 2.3. Fluorescence Quantum Yield of ICAA Derivatives in Different Solvents 

**Figure 5:**
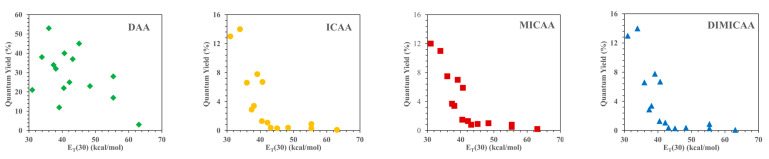
Dependence of quantum fluorescence yields of the 1,5-disubstituted anthracene dyes on the empirical parameter of solvent polarity E_T_(30).

An essential property, which also determines the practical applicability of any fluorophore, is their quantum yield (√_f_), i.e., the ratio of the emitted and absorbed photons. The fluorescence quantum yield of ICAAs is strongly influenced by the polarity of the solvent (Table 3). The starting anthracene-diamine is highly fluorescent in every solvent (√_f_ = 12–53%); however, in water, the quantum yield drops to only 3%. In contrast, √_f_ rarely exceeds 10% in nonpolar solvents for the isocyano derivatives ICAA, MICAA and DIMICAA, and they are practically nonfluorescent in solvents more polar than dioxane (Figure 4). In polar solvents, the quantum yields of ICAA-DIMICAA are close to only 1%; moreover, it drops to only √_f_ = 0.1–0.2% in water. The fluorescence quantum yield of DAA, ICAA, MICAA and DIMICAA in different solvents was correlated with the empirical Dimroth polarity parameters ET(30) of the solvents (Figure 5). While no clear correlation was obtained for the diamine (DAA), the isocyano derivatives (ICAA-DIMICAA) show almost identical behavior, i.e., a sharp decrease in √_f_ between E_T_(30) = 30–40 (kcal/mol), followed by a constant minimum range above E_T_(30) > 40 (kcal/mol). A very similar behavior was described for 1,8-naphthalimides [34,35]. This phenomenon implies the potential application of ICAAs to probe the polarity of the medium. The very low quantum yield in water is favorable in practical applications, such as cell-staining, resulting in a reduced background fluorescence as we have shown previously for the ICAN dyes [26]. The reduced fluorescence in polar solvents can also be practical for the selective staining of different nonpolar cell compartments such as cell membrane and/or nucleus.

**Figure 6:**
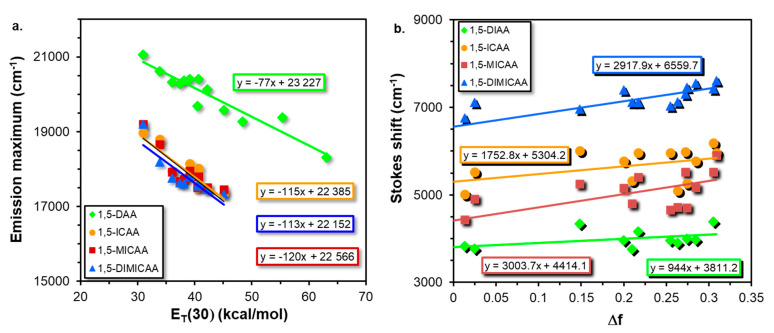
Variation of the fluorescence emission maximum with the empirical solvent polarity parameter E_T_(30) (**a**) and the Lippert–Mataga (LM) (**b**) plots for the 1,5-disubstituted anthracene dyes.

Two of the most common ways to quantify the solvatochromic effect in solvents of different polarity is to plot the fluorescence emission maxima (ν_Em_) as a function of the empirical solvent polarity parameter E_T_(30) [36] and the Lippert–Mataga (LM) plot [37,38], which are presented in Figure 6.

Interestingly, two groups can be identified: one belonging to the diamine (DAA) and the other to the isocyano (ICAA-DIMICAA) dyes (Figure 6a). In both cases, the correlation is linear between the fluorescence emission maximum and E_T_(30) for all the anthracene fluorophores. It can be surmised from the corresponding slopes that ICAA, MICAA and DIMICAA have almost the same solvatochromic shift (113–120 kcal^−1^cm^−1^mol), while this value is significantly lower for the 1,5-DAA (77 kcal^−1^cm^−1^mol) supporting the ICT character of the emission state of ICAA-DIMICAA, which is in good agreement with the computational findings presented in Table 2. It should be noted, however, that because of their strong H-bond donating character (i.e., the solvatochromic response is strongly modulated by specific interactions), protic solvents (iPrOH, MeOH and H_2_O) were not included in the plot. The plots containing all the solvents are found in the Supplementary Information (Figure S89).

Lippert–Mataga’s Equation (1), which is based on the correlation of energy difference between the ground and excited states (Stokes’ shift) with the solvent orientation polarizability (Δ*f*), can be used to investigate the change in dipole moment between the excited singlet state (*µ_e_*) and ground state (*µ_g_*).

(1) $$\Delta \nu_{SS}=\frac{2{\left(\mu_{e}-\mu_{g}\right)}^{2}}{4\pi \varepsilon_{0}hca_{0}^{3}}\Delta f+const$$

where $\Delta \nu_{SS}\;$(in cm^−1^) is the Stokes shift, ε_0_, *h* and c are the vacuum permittivity (8.8541 × 10^−12^ C⋅V^−1^m^−1^), Planck constant (h = 6.626 × 10^−34^ Js), speed of light (c = 299,792,458 m s^−1^), respectively, and the dipole moment is given in Debye. The Onsager cavity radius (a_0_), which closely reflects to the radius of a spherical cavity the fluorophore molecule occupies, is either obtained from quantum chemical calculations or by using Suppan’s Equation (2) [39],

(2) $$a={\left(\frac{3M}{4\pi \rho N_{A}}\right)}^{1/3}$$

where *M* is the molecular weight of the fluorophore, *N_A_* is the Avogadro’s constant and *ρ* is the density. Δ*f* stands for the orientation polarizability defined as:

(3) $$\Delta f=\left(\frac{\varepsilon -1}{2\varepsilon +1}-\frac{n^{2}-1}{2n^{2}+1}\right)$$

where *ε* and *n* are the dielectric constant and the refractive index of the solvent, respectively.

After plotting the Stoke’s shift values versus Δ*f* (Figure 6b), the dipole moment change can be calculated from the slope of the plot as:

(4) $$|\mu_{e}-\mu_{g}|=\sqrt{slope\times \frac{4\pi \varepsilon_{0}hca^{3}}{2}}$$

According to Figure 6b, MICAA has the highest slope (3003 cm^−1^) obtained from the LM plot, which is closely followed by DIMICAA (2917 cm^−1^). The smallest slope belongs to DAA (944 cm^−1^), which is almost twice as small as the corresponding values for ICAA (1752 cm^−1^). In addition, the Stokes shifts at Δf_LM_ = 0, i.e., the intercepts of the lines determined from the LM plots, decrease in the order of DIMICAA > ICAA > MICAA > DAA. The difference between the excited and the ground state dipole moments, i.e., Δμ = μ_E_ − μ_G,_ have been calculated according to Equation (4) and by using the DFT results (Table 2). To calculate Δμ, first, *a* has to be determined, and instead of using Equation (2), it has been associated with the half distance between the amino and isocyano groups of the corresponding optimized geometries (Table 2). The fully DFT-based dipole moment difference (μ_E_ − μ_G_)**_DFT_** computed in hexane, dioxane and water are summarized in Table 4 along with the experimental values (μ_E_ − μ_G_)**_LM_** calculated from Figure 6b using Equation (4).

**Figure 7:**
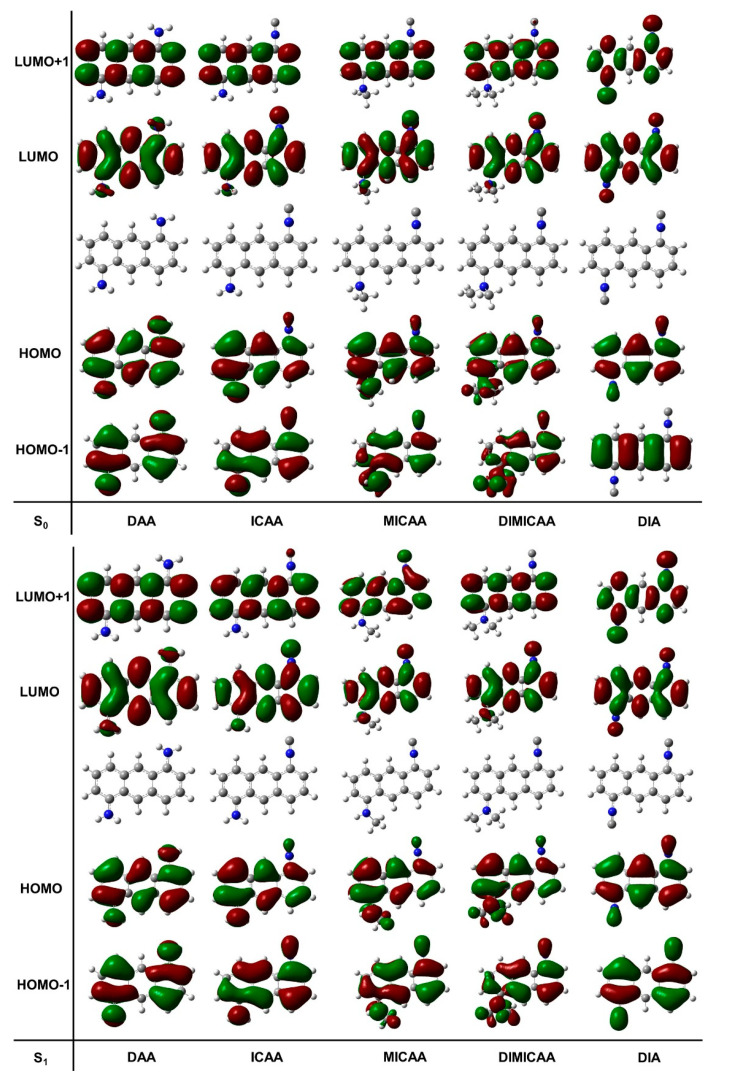
Representation of the relevant molecular orbitals (MO) for the relaxed ground (S_0_(solv, s_0_)) and excited states (S_1_(solv, s_1_)) of the 1,5-disubstituted anthracene dye structures in dioxane (isovalue for electron density was set to 0.000400 a.u.).

It is evident from the data of Table 4 that the simultaneous presence of one amino and one isocyano group (ICAA, MICAA and DIMICAA) yields a higher dipole moment change than in the case of DAA, and in line with the expectations, DIA with the symmetric structure does not have a dipole moment or dipole moment change. However, contrary to the expectations and DFT calculations, DAA also has a significant dipole moment change (Δμ = 2.22 D). Amongst the molecules studied, only the structure of DIA is planar, and the isocyano groups linearly attached to the ring structure resulted in a Cs symmetric molecule. On the other hand, the structure of DAA has unique structural features in such a way that the aromatic rings are slightly bent which come from the interaction of amine nitrogens with the ring structure. One of the hydrogens in each amine group is almost in the rings’ plane while the other one sticks out of the plane (Figure 7). Since two amine groups are attached to this structure, these hydrogens can be on one side of the rings or on opposite sides. Since the latter conformer has only a permanent dipole moment, we have considered it.

Interestingly, the Δμ values calculated from the Lippert–Mataga equation are half than those obtained by DFT. However, the change of the values from the LM plots are in good agreement with the fully DFT-based results. That is, Δμ changes in the order of 0 D = DIA < DAA < ICAA < MICAA ≅ DIMICAA. In addition, Δμ values are lower for ICAA, MICAA and DIMICAA, while in the case of DAA, Δμ is higher compared to the results in water.

TD-DFT calculations were performed to obtain a deeper understanding of the electronic behavior of the excited states of the studied structures (Figure 7). Optimizations have been carried out on the ground and excited state geometries. The HOMO, LUMO, HOMO-1, LUMO+1 molecular orbitals for all the dye structures in this study are depicted in Figure 7. There are no significant differences between the corresponding ground and excited state molecular orbitals.

**Figure 8:**
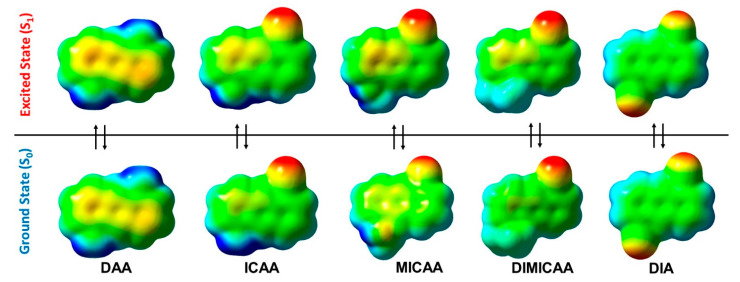
Molecular electrostatic potential (MESP) isosurfaces (isovalue = 0.02 a.u.) for the relaxed ground (S_0_(solv, s_0_)) and excited states (S_1_(solv, s_1_)) of the 1,5-disubstituted anthracene dye structures in dioxane (blue color corresponds to +0.045. a.u. (ca. +120 kJ/mol) potential while red represents −0.045 a.u. (ca. −120 kJ/mol)).

Molecular electrostatic potential (MESP) isosurfaces have also been created for the relaxed ground and excited states as shown in Figure 8. There is no visible difference in the MESPs of the S_0_ and S_1_ in the case of the diamino and diisocyano structures, DAA and DIA, respectively (Figure 8). However, a slight difference between the ground and excited state MESPs of ICAA, MICAA and DIMICAA can be seen. However, these cannot be associated with significant changes in the geometries. The largest difference between the S_0_ and S_1_ MESPs occurs on the isocyano group in case of MICAA, where a rotation of the methylated amino group is also experienced.

## 3. Materials and Methods

Acetone, dichloromethane (DCM), hexane, 2-propanol (iPrOH), toluene, ethyl-acetate (EtOAc) (reagent grade, Molar Chemicals, Hungary) were purified by distillation. Acetonitrile (MeCN), tetrahydrofuran (THF), methanol (MeOH), dimethyl formamide (DMF), dimethyl sulfoxide (DMSO), pyridine (HPLC grade, VWR, Germany), cyclohexane, 1,4-dioxane (reagent grade, Reanal, Hungary), chloroform, 1,5-diaminoanthraquinone (Sigma-Aldrich, Germany) were used without further purification.

*NMR*: ^1^H and ^13^C-NMR spectra were recorded in CDCl_3_ and DMSO-d_6_ at 25 °C on a Bruker Avance DRX-400 and a Bruker AM 360 spectrometer at 400 and 360 MHz, respectively, with tetramethylsilane as the internal standard.

*UV–vis*: The UV–vis spectra were recorded on an Agilent Cary 60 spectrophotometer (Agilent, Santa Clara, CA, USA) in a quartz cuvette of 1.00 cm optical length. A 3.00 cm^3^ solution was prepared from the sample.

### 3.1. Synthesis

#### 3.1.1 1-Amino-5-Isocyanoanthracene (ICAA) and 1,5-Diisocyanoanthracene (DIA)

In a 250 mL round-bottom flask, 1,5-diaminoanthracene* (1.00 g, 4.80 mmol) and potassium hydroxide (2.69 g, 48.0 mmol) suspended in 50 mL chloroform and 50 mL toluene were stirred for 1 h, then extracted with water. The organic phase was dried over anhydrous magnesium sulfate, and the solvent was removed on a rotary evaporator. The crude product was purified on a column filled with normal-phase silica gel, using dichloromethane as eluent. Yield: 0.16 g, 15% ICAA (orange-red crystals) and 0.12 g, 11% DIA (pale yellow crystals).

**Figure 9:**
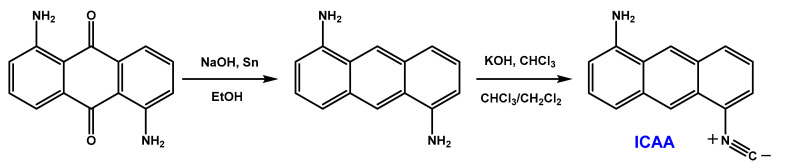
The synthesis of DAA and ICAA from 1,5-diaminoanthraquinone.

*1,5-Diaminoanthracene was synthesized by the reduction of 1,5-Diaminoanthraquinone according to the literature (Figure 9) [9].

ICAA

^1^H NMR (360 MHz, DMSO) δ = 8.95 (s, 1H), 8.50 (s, 1H), 8.16 (d, *J* = 8.6 Hz, 1H), 7.85 (d, *J* = 7.0 Hz, 1H), 7.48 (dd, *J* = 17.2, 8.5 Hz, 2H), 7.37 (t, *J* = 7.8 Hz, 1H), 6.73 (d, *J* = 7.2 Hz, 1H), 6.09 (s, 2H) ppm (Figure S17).

^13^C NMR (95 MHz, DMSO) δ = 167.89 (C_NC_), 145.32 (C_1,5_), 133.93 (C_8a_), 131.43 (C_6_), 129.80 (C_4a_), 128.91 (C_8_), 125.43 (C_3_), 125.22 (C_9a_), 124.13 (C_10a_), 123.90 (C_7_), 123.34 (C_9_), 120.65 (C_4_), 115.90 (C_10_), 106.18 (C_2_) ppm (Figure S17).

DIA

^1^H NMR (360 MHz, DMSO) δ = 8.96 (s, 1H), 8.54 (d, *J* = 8.6 Hz, 1H), 8.02 (d, *J* = 7.1 Hz, 1H), 7.70 (t, *J* = 7.9 Hz, 1H) ppm (Figure S35).

#### 3.1.2 1-N-Methylamino-5-Isocyanoanthracene (MICAA) and 1-N,N-Dimethylamino-5-Isocyanoanthracene (DIMICAA)

A 250 mL round-bottomed flask, equipped with a magnetic stir bar, was charged with 1-Amino-5-isocyanoanthracene (1.00 g, 4.58 mmol), potassium hydroxide (2.82 g, 50.4 mmol) and absolute dimethyl formamide freshly distilled over phosphorous pentoxide (50 mL). Methyl iodide (2.85 mL, 45.8 mmol) was added to the solution, then the flask was flushed with argon and sealed with a rubber septum. The reaction mixture was stirred at room temperature, protected from light. After 2 days, 200 mL methylene chloride and 5% ammonia were added, and the solution was extracted 5 times with water, then the organic phase was dried over anhydrous magnesium sulfate. Solvent was removed on a rotary evaporator and the residue was purified on a normal-phase silica gel-filled column, using methylene chloride: hexane (1:1) as eluent. Yield: 0.23 g, 22% MICAA (orange crystals) and 0.35 g, 31% DIMICAA (yellow-orange crystals).

MICAA

^1^H NMR (360 MHz, CDCl_3_) δ = 8.64 (s, 1H), 8.40 (s, 1H), 8.02 (d, J = 8.5 Hz, 1H), 7.59 (d, J = 7.0 Hz, 1H), 7.55–7.43 (m, 2H), 7.41–7.33 (m, 1H), 6.59 (d, J = 6.7 Hz, 1H), 4.66 (s, 1H), 3.10 (s, 3H) ppm (Figure S53).

DiMICAA

^1^H NMR (360 MHz, CDCl_3_) δ = 8.84 (s, 1H), 8.67 (s, 1H), 8.08 (d, *J* = 8.6 Hz, 1H), 7.75 (d, *J* = 8.5 Hz, 1H), 7.57 (d, *J* = 6.9 Hz, 1H), 7.46 (dd, *J* = 8.4, 7.3 Hz, 1H), 7.37 (dd, *J* = 8.5, 7.2 Hz, 1H), 7.07 (d, *J* = 7.1 Hz, 1H), 2.97 (s, 6H) ppm (Figure S71).

^13^C NMR (91 MHz, CDCl_3_) δ = 167.38 (C_NC_), 150.87 (C_1,5_), 134.06 (C_8a_), 130.95 (C_6_), 130.69 (C_4a_), 128.58 (C_9a_), 126.84 (C_8_), 125.66 (C_10a_), 124.38 (C_3_), 124.27 (C_7_), 123.53 (C_9_), 123.36 (C_4_), 122.28 (C_10_), 113.77 (C_2_), 45.24 (C_CH3_) ppm (Figure S71).

### 3.2. Fluorescence Measurements

Steady-state fluorescence measurements were carried out using a Jasco FP-8200 fluorescence spectrophotometer equipped with a Xe lamp light source. The excitation and emission spectra were recorded at 20 °C, using 2.5 nm excitation, 5.0 nm emission bandwidth and 200 nm/min scanning speed. Fluorescence quantum yields (Φ_F_) were calculated by using quinine-sulfate as the reference, using the following equation:

(5) $$\Phi_{F}=\Phi r*\frac{I}{I_{ref}}*\frac{A_{ref}}{A}*\frac{n^{2}}{n_{ref}^{2}}$$

where Φ_r_ is the quantum yield of the reference compound (quinine-sulfate in 0.1 mol/L sulfuric acid, absolute quantum efficiency (Φ_r_ = 55%)), *n* is the refractive index of the solvent, *I* is the integrated fluorescence intensity and *A* is the absorbance at the excitation wavelength. The absorbances at the wavelength of excitation were kept below *A* = 0.1 in order to avoid inner filter effects.

For UV–vis and fluorescence measurements, the investigated compounds were dissolved in acetonitrile at a concentration of 1.19 mM and were diluted to 2.38 × 10^−5^ M and 4.76 × 10^−6^ M in the solvents in interest. The spectra were processed using Spectragryph software [40].

### 3.3. Density Functional Theory (DFT) Calculations

To obtain an explanation for the spectral changes which take into account the electronic structure of the species, a previously tested calculation protocol was adopted [41,42,43]. The geometry optimization of solvated molecules (DAA-DIA) was carried out by using M06 [44] density functional combined with triple-ζ Karlsruhe basis set (TZVP) [45]. The solvent effect on the geometries was mimicked by integral equation formalism of the polarizable continuum model (IEF-PCM) and the solvent cavity for hexane, dioxan and water (solv) was constructed as implemented in Gaussian09 software package. [46] Normal mode analysis was performed to ensure that optimized geometry corresponds to real minima of the potential energy surface (noted as S_0_(solv, s_0_)). The harmonic vibrational wavenumbers were used to obtain the thermochemical properties. The first singlet vertical excitation energies (VEE) were computed for each molecule by time dependent (TD) counterpart of M06/TZVP (TD-M06/TZVP). To properly account for nonequilibrium solvation, the corrected linear response formalism was employed [47] via PisaLR protocol [www.dcci.unipi.it/molecolab/tools/white-papers/pisalr (last accessed on 29 December 2021.)]. The calculated VEE values (for S_1_(solv, s_0_)) were then compared with the wavenumber at the maximum in experimental excitation spectra (λ_ex,max_). The geometry of the first singlet excited states (S_1_) of molecules were optimized at TD-M06/TZVP level of theory considering equilibrium solvation (S_1_(solv, s_1_)). The local minimum nature of each optimized S_1_ structures were verified by normal mode analysis using numerical Hessian. The maximum of the emission spectra (λ_em,max_) was approximated as energy difference of the first singlet excited (S_1_(solv, s_1_)) and ground states (vertical de-excitation) in such a way that nonequilibrium solvation is considered for the ground state (S_0_(solv, s_1_)). For both S_0_(solv, s_0_)) and S_1_(solv, s_1_)), the magnitude of the permanent electric dipole moments (noted μ_S0_ and μ_S1_, respectively) and molecular electrostatic potentials (MESP) of the studied molecules were also calculated by using the M06/TZVP(IEF-PCM) level of theory.

## 4. Conclusions

The electronic absorption, solvatochromic and photophysical properties of novel 1-amino-5-isocyanoanthracenes (ICAAs) and the synthetically important 1,5-diaminoanthracene (DAA) have been studied. The new isocyano-aminoanthracenes (and to a limited extent DAA) clearly show positive solvatochromic behavior. Contrary to expectations, the symmetrical DAA behaves as a weak solvatochromic dye, and its fluorescence is shifted bathochromically with increasing solvent polarity (λ_Em_ = 475 and 511 nm in hexane and DMSO, respectively). DAA is strongly fluorescent in most of the solvents Φf = 21–53% (hexane-dioxane) and its solvatochromic behavior may be explained by the significant change of its dipole moment between excited and ground states (Δμ = 2.22 D). DAA has unique structural features because the aromatic rings are slightly distorted due to the interaction of amine nitrogens with the ring. By replacing one of the amino groups of DAA with isocyanide, the absorption maximum is redshifted 10–20 nm in the case of ICAA and monomethylation of the remaining amino group adds another 10 nm redshift in the case of MICAA. Interestingly, dimethylation results in an opposite effect, and the absorption maxima are found approximately 10 nm lower than in the case of DAA. The molar absorption coefficients in various solvents were slightly lower for ICAA than those of DAA and were found to decrease in the order of DAA > DIMICAA > MICAA > ICAA. The differences in the dipole moments of the excited and ground states were determined by using the Lippert–Mataga equation and DFT calculations. According to DFT calculations, the ICT is limited within the ICAA molecules, that is, no HOMO-LUMO pairs were found that were dominantly located on the amino and isocyano groups. Instead, ICT to the aromatic ring was observed, which is in line with the results obtained from MESP isosurfaces. In contrast to DAA, the Φ_f_ rarely exceeds 10% in nonpolar solvents for the isocyano derivatives, and they are practically nonfluorescent in solvents more polar than dioxane. This phenomenon implies the potential application of ICAAs to probe the polarity of the medium. Furthermore, the very low quantum yield in water is favorable in cell-staining, and thus, by using the studied species, even the selective visualization of different non-polar cell compartments (e.g., cell membrane and/or nucleus) is feasible.

## Figures and Tables

**Table 1:** The absorption maximum wavelengths (λ_Abs_) and the molar extinction coefficients at λ_Abs_ (ε) determined in various solvents for the 1,5-diaminoanthracene (**DAA**) and its isocyano derivatives (**ICAA**, **MICAA**, **DIMICAA**). The dielectric constants of the solvents (ε_r_) are listed next to the solvent names.

Solvent (ε_r_)	DAA		ICAA		MICAA		DIMICAA	
	λ_Abs_ (nm)	ε ×10^−3^ (M^−1^cm^−1^)	λ_Abs_ (nm)	ε ×10^−3^ (M^−1^cm^−1^)	λ_Abs_ (nm)	ε ×10^−3^ (M^−1^cm^−1^)	λ_Abs_ (nm)	ε ×10^−3^ (M^−1^cm^−1^)
n-Hexane (1.89)	403	4.4	412	3.9	432	3.9	397	4.9
Toluene (2.38)	409	5.4	420	3.8	433	4.1	401	4.8
DCM (8.93)	407	4.7	417	3.9	431	4.0	404	4.8
2-propanol (17.9)	403	4.6	426	3.6	434	3.9	403	4.9
THF (7.58)	416	5.0	435	3.1	445	3.7	404	4.8
Chloroform (4.81)	404	4.6	414	3.8	431	3.9	404	4.8
EtOAc (6.02)	411	4.8	425	3.2	437	4.0	400	4.9
Dioxane (2.25)	415	5.2	426	3.7	438	4.1	402	5.1
Acetone (20.7)	415	4.8	430	3.2	441	3.7	400	4.6
Methanol (32.7)	406	4.4	426	3.3	427	3.7	398	4.9
Pyridine (12.4)	423	5.7	426	3.1	451	4.0	407	4.8
Acetonitrile (37.5)	410	5.0	422	3.7	434	4.0	403	4.9
DMF (36.7)	421	5.4	442	3.1	449	3.9	404	4.9
DMSO (46.7)	426	5.3	444	3.4	451	4.0	409	4.9
Water (80.1)	395	4.6	410	3.6	415	3.4	400	3.9

**Table 2:** Dipole moments (*μ*) and distances between the N-atoms *d(N-N)* of the functional groups of compounds DAA-DIA in solvents of different polarity, obtained by DFT calculations.

		Hexane		Dioxane		Water	
		d (N-N)	µ	d (N-N)	µ	d (N-N)	µ
**S_0_ (Ground State)**		Angstrom	Debye	Angstrom	Debye	Angstrom	Debye
	**DAA**	7.492	2.03	7.493	2.07	7.501	2.49
	**ICAA**	7.473	6.05	7.474	6.19	7.478	7.29
	**MICAA**	7.479	5.55	7.480	5.67	7.488	6.50
	**DIMICAA**	7.476	5.52	7.477	5.64	7.482	6.50
	**DIA**	7.446	0.00	7.447	0.00	7.452	0.00
**S_1_opt (Excited State)**		Angstrom	Debye	Angstrom	Debye	Angstrom	Debye
	**DAA**	7.502	1.64	7.504	1.66	7.514	1.84
	**ICAA**	7.462	12.15	7.463	12.45	7.472	14.64
	**MICAA**	7.541	12.98	7.543	13.32	7.554	15.81
	**DIMICAA**	7.510	13.02	7.511	13.36	7.519	15.78
	**DIA**	7.445	0.00	7.446	0.00	7.451	0.00

**Table 3:** The fluorescence emission maxima (λ_Em_), the quantum yields (Φ_f_) and the Stokes shifts ($\Delta {\bar{\nu }}_{SS}$ ) determined in various solvents for the 1,5-disubstituted anthracene derivatives.

	1,5-DAA			1,5-ICAA			1,5-MICAA			1,5-DIMICAA		
**Solvent**	**λ_Em_ (nm)**	**√_f_ (%)**	$\Delta {\bar{\nu }}_{SS}$ **(cm^−1^)**	**λ_Em_ (nm)**	**√_f_ (%)**	$\Delta {\bar{\nu }}_{SS}$ **(cm^−1^)**	**λ_Em_ (nm)**	**√_f_ (%)**	$\Delta {\bar{\nu }}_{SS}$ **(cm^−1^)**	**λ_Em_ (nm)**	**√_f_ (%)**	$\Delta {\bar{\nu }}_{SS}$ **(cm^−1^)**
n-Hexane	475	21	3761	527	13	5297	521	12	3954	521	14	5995
Toluene	485	38	3831	532	14	5013	536	11	4438	550	11	6756
DCM	490	40	4162	555	6.7	5963	562	5.9	5408	567	5.9	7116
2-propanol	519	23	5546	572	0.4	5992	571	1.0	5528	571	1.7	7301
THF	493	34	3754	566	2.9	5321	566	3.7	4804	567	4.5	7116
Chloroform	490	12	4717	551	7.8	6006	557	7.0	5249	562	9.7	6959
EtOAc	491	32	3964	563	3.4	5767	564	3.4	5153	568	3.8	7394
Dioxane	492	53	3771	557	6.6	5521	558	7.5	4910	563	9.0	7114
Acetone	497	25	3976	572	1.1	5773	571	1.3	5163	573	1.6	7548
Methanol	516	17	5251	571	0.3	5961	571	0.5	5906	571	0.8	7612
Pyridine	508	22	3956	571	1.3	5961	571	1.5	4660	570	3.7	7026
Acetonitrile	500	28	4390	571	0.9	6184	571	0.8	5528	575	0.9	7423
DMF	506	37	3990	576	0.4	5263	572	0.8	4820	578	0.9	7451
DMSO	511	45	3905	576	0.3	5160	573	0.9	4720	577	1.0	7119
Water	546	3.1	7001	530	0.1	-	559	0.2	6207	567	0.2	7363

**Table 4:** The dipole moment difference between the excited and the ground state (μ_E_ − μ_G_)_DFT_) calculated by DFT (in various solvents), and the dipole moment differences in the excited and ground state (μ_E_ − μ_G_)_LM_) determined by the Lippert–Mataga equation (Equation (4)).

	(μ_E_ − μ_G_)_DFT_ (D)			(μ_E_ − μ_G_)_LM_ (D)
	Hexane	Dioxane	Water	
**DAA**	−0.39	−0.41	−0.65	2.22
**ICAA**	6.10	6.26	7.35	3.07
**MICAA**	7.31	7.77	9.31	3.98
**DIMICAA**	7.49	7.72	9.28	3.88
**DIA**	0.00	0.00	0.00	0.00

## Data Availability

All data generated or analyzed during this study are included in this published article (and its Supplementary Information files) or are available from the corresponding author on reasonable request.

## Supplementary Materials

The following are available online at https://www.mdpi.com/article/10.3390/ijms23031315/s1.
