# Supplementary material for: Optical Study of Solvatochromic Isocyanoaminoanthracene Dyes and 1,5-Diaminoanthracene
Source: Int J Mol Sci. 2022 Jan 24;23(3):1315. doi: 10.3390/ijms23031315 (PMC8835764; doi:10.3390/ijms23031315)
Supplement: Supplementary file 1 [file ijms-23-01315-s001.zip › ijms-1557179-supplementary.pdf]

Optical study of solvatochromic isocyanoaminoanthracene dyes and 1,5-diaminoanthracene

Miklós Nagy [1, *], Béla Fiser[1], Milán Szőri[1*], László Vanyorek[1], Béla Viskolcz[1]

[1] *Institute of Chemistry, University of Miskolc, Miskolc-Egyetemváros, 3515-Hungary; kemfiser@uni-miskolc.hu (B.F.)* kemvanyi@uni-miskolc.hu (L.V.); *bela.viskolcz@uni-miskolc.hu (B.V.)*

* Correspondence: nagy.miklos@uni-miskolc.hu (M.N.); milan.szori@uni-miskolc.hu (M.S.)

Supporting information

Table of Contents

Chapter 1. 1,5-diaminoanthracene (DAA) 5

Figure S1. The emission spectra of 1,5-diaminoanthracene (**DAA**) recorded in solvents of different polarity. (c= 5×10^{-5} M, T=20 ºC, V=3.00 ml). 5

Figure S2. The excitation spectra of 1,5-diaminoanthracene (**DAA**) recorded in solvents of different polarity. (c= 5×10^{-5} M, T=20 ºC, V=3.00 ml). 5

Figure S3. UV-Vis absorbance spectrum of DAA recorded in hexane 6

Figure S4. UV-Vis absorbance spectrum of DAA recorded in toluene 6

Figure S5. UV-Vis absorbance spectrum of DAA recorded in methylene-chloride 7

Figure S6. UV-Vis absorbance spectrum of DAA recorded in acetone 7

Figure S7. UV-Vis absorbance spectrum of DAA recorded in methanol 8

Figure S8. UV-Vis absorbance spectrum of DAA recorded in chloroform 8

Figure S9. UV-Vis absorbance spectrum of DAA recorded in pyridine 9

Figure S10. UV-Vis absorbance spectrum of DAA recorded in acetonitrile 9

Figure S11. UV-Vis absorbance spectrum of DAA recorded in 2-propanol 10

Figure S12. UV-Vis absorbance spectrum of DAA recorded in tetrahydrofuran 10

Figure S13. UV-Vis absorbance spectrum of DAA recorded in dioxane 11

Figure S14. UV-Vis absorbance spectrum of DAA recorded in dimethylformamide 11

Figure S15. UV-Vis absorbance spectrum of DAA recorded in dimethyl sulfoxide 12

Figure S16. UV-Vis absorbance spectrum of DAA recorded in water 12

Chapter 2. 1-amino-5-isocyanoanthracene (ICAA) 13

Figure S17. ^{1}H-NMR (top) and ^{13}C-NMR (bottom) spectra of ICAA recorded at 20 °C in DMSO-d_6 13
Figure S18. UV-Vis absorbance spectrum of ICAA recorded in acetone 14
Figure S19. UV-Vis absorbance spectrum of ICAA recorded in acetonitrile 14
Figure S20. UV-Vis absorbance spectrum of ICAA recorded in dichloromethane 15
Figure S21. UV-Vis absorbance spectrum of ICAA recorded in 1,4-dioxane 15
Figure S22. UV-Vis absorbance spectrum of ICAA recorded in DMF 16
Figure S23. UV-Vis absorbance spectrum of ICAA recorded in DMSO 16
Figure S24. UV-Vis absorbance spectrum of ICAA recorded in ethyl acetate 17
Figure S25. UV-Vis absorbance spectrum of ICAA recorded in hexane 17
Figure S26. UV-Vis absorbance spectrum of ICAA recorded in 2-propanol 18
Figure S27. UV-Vis absorbance spectrum of ICAA recorded in chloroform 18
Figure S28. UV-Vis absorbance spectrum of ICAA recorded in methanol 19
Figure S29. UV-Vis absorbance spectrum of ICAA recorded in pyridine 19
Figure S30. UV-Vis absorbance spectrum of ICAA recorded in THF 20
Figure S31. UV-Vis absorbance spectrum of ICAA recorded in toluene 20
Figure S32. UV-Vis absorbance spectrum of ICAA recorded in water 21
Figure S33. The emission spectra of 1-amino-5-isocyanoanthracene (**ICAA**) recorded in solvents of different polarity. (c= 5×10^{-5} M, T=20 ºC, V=3.00 ml). 22
Figure S34. The excitation spectra of 1-amino-5-isocyanoanthracene (**ICAA**) recorded in solvents of different polarity. (c= 5×10^{-5} M, T=20 ºC, V=3.00 ml). 22
Chapter 3. 1,5-diisocyanoanthracene (DIA) 23
Figure S35. ^{1}H-NMR spectrum of DIA recorded at 20 °C in DMSO-d_6 23
Figure S36. UV-Vis absorbance spectrum of DIA recorded in acetone 23
Figure S37. UV-Vis absorbance spectrum of DIA recorded in acetonitrile 24
Figure S38. UV-Vis absorbance spectrum of DIA recorded in dichloromethane 24
Figure S39. UV-Vis absorbance spectrum of DIA recorded in 1,4-dioxane 25
Figure S40. UV-Vis absorbance spectrum of DIA recorded in DMF 25
Figure S41. UV-Vis absorbance spectrum of DIA recorded in DMSO 26
Figure S42. UV-Vis absorbance spectrum of DIA recorded in ethyl acetate 26
Figure S43. UV-Vis absorbance spectrum of DIA recorded in hexane 27
Figure S44. UV-Vis absorbance spectrum of DIA recorded in 2-propanol 27
Figure S45. UV-Vis absorbance spectrum of DIA recorded in chloroform 28
Figure S46. UV-Vis absorbance spectrum of DIA recorded in methanol 28

Figure S47. UV-Vis absorbance spectrum of DIA recorded in pyridine 29
Figure S48. UV-Vis absorbance spectrum of DIA recorded in THF 29
Figure S49. UV-Vis absorbance spectrum of DIA recorded in toluene 30
Figure S50. UV-Vis absorbance spectrum of DIA recorded in water 30
Figure S51. The emission spectra of 1,5-diisocyanoanthracene (**DIA**) recorded in solvents of different polarity. (c= 5×10^{-5} M, T=20 ºC, V=3.00 ml). 31
Figure S52. The excitation spectra of 1,5-diisocyanoanthracene (**DIA**) recorded in solvents of different polarity. (c= 5×10^{-5} M, T=20 ºC, V=3.00 ml). 31
Chapter IV. 1-N-methylamino-5-isocyanoanthracene (MICAA) 33
Figure S53. ^{1}H-NMR spectra of MICAA recorded at 20 °C in CDCl3 33
Figure S54. UV-Vis absorbance spectrum of MICAA recorded in acetone 34
Figure S55. UV-Vis absorbance spectrum of MICAA recorded in acetonitrile 34
Figure S56. UV-Vis absorbance spectrum of MICAA recorded in dichloromethane 35
Figure S57. Dioxane UV-Vis absorbance spectrum of MICAA recorded in dioxane 35
Figure S58. UV-Vis absorbance spectrum of MICAA recorded in DMF 35
Figure S59. UV-Vis absorbance spectrum of MICAA recorded in DMSO 36
Figure S60. UV-Vis absorbance spectrum of MICAA recorded in ethyl-acetate 37
Figure S61. UV-Vis absorbance spectrum of MICAA recorded in hexane 37
Figure S62. UV-Vis absorbance spectrum of MICAA recorded in isopropanole 37
Figure S63. UV-Vis absorbance spectrum of MICAA recorded in chloroform 38
Figure S64. UV-Vis absorbance spectrum of MICAA recorded in methanol 38
Figure S65. UV-Vis absorbance spectrum of MICAA recorded in pyridine 39
Figure S66. UV-Vis absorbance spectrum of MICAA recorded in tetrahydrofurane 39
Figure S67. UV-Vis absorbance spectrum of MICAA recorded in toluene 40
Figure S68. UV-Vis absorbance spectrum of MICAA recorded in water 40
Figure S69. The emission spectra of 1-N-methylamino-5-isocyanoanthracene (MICAA) recorded in solvents of different polarity. (c= 5×10^{-5} M, T=20 ºC, V=3.00 ml). 41
Figure S70. The excitation spectra of 1-N-methylamino-5-isocyanoanthracene (MICAA) recorded in solvents of different polarity. (c= 5×10^{-5} M, T=20 ºC, V=3.00 ml). 41
Chapter V. 1-N,N-dimethylamino-5-isocyanoanthracene (DIMICAA) 42
Figure S71. ^{1}H-NMR (top) and ^{13}C-NMR (bottom) spectra of 1-N,N-dimethylamino-5-isocyanoanthracene (DIMICAA) recorded at 20 °C in $CDCl_3$ 42
Figure S72. The emission spectra of 1-N,N-dimethylamino-5-isocyanoanthracene (DIMICAA) recorded in solvents of different polarity. (c= 5×10^{-5} M, T=20 ºC, V=3.00 ml). 43

Figure S73. The excitation spectra of 1-N,N-dimethylamino-5-isocyanoanthracene (DIMICAA) recorded in solvents of different polarity. (c= 5×10^{-5} M, T=20 ºC, V=3.00 ml). 43
Figure S74. The UV-vis spectrum of (DIMICAA) recorded in acetone 44
Figure S75. The UV-vis spectrum of (DIMICAA) recorded in acetonitrile 45
Figure S76. The UV-vis spectrum of (DIMICAA) recorded in methylene-chloride 45
Figure S77. Dioxane The UV-vis spectrum of (DIMICAA) recorded in dioxane 46
Figure S78. The UV-vis spectrum of (DIMICAA) recorded in DMF 46
Figure S79. The UV-vis spectrum of (DIMICAA) recorded in DMSO 46
Figure S80. The UV-vis spectrum of (DIMICAA) recorded in EtOAc 47
Figure S81. The UV-vis spectrum of (DIMICAA) recorded in hexane 47
Figure S82. The UV-vis spectrum of (DIMICAA) recorded in isopropanol 48
Figure S83. The UV-vis spectrum of (DIMICAA) recorded in choloroform 48
Figure S84. The UV-vis spectrum of (DIMICAA) recorded in methanol 49
Figure S85. The UV-vis spectrum of (DIMICAA) recorded in pyridine 49
Figure S86. The UV-vis spectrum of (DIMICAA) recorded in tetrahydrofurane 51
Figure S87. The UV-vis spectrum of (DIMICAA) recorded in toluene 51
Figure S88. The UV-vis spectrum of (DIMICAA) recorded in water 51
Figure S89. Variation of the fluorescence emission maximum with the empirical solvent polarity parameter $E_T(30)$ (**a**) and the Lippert-Mataga (LM) (**b**) plots for the 1,5-disubstituted anthracene dyes.. 52

Chapter 1. 1,5-diaminoanthracene (DAA)

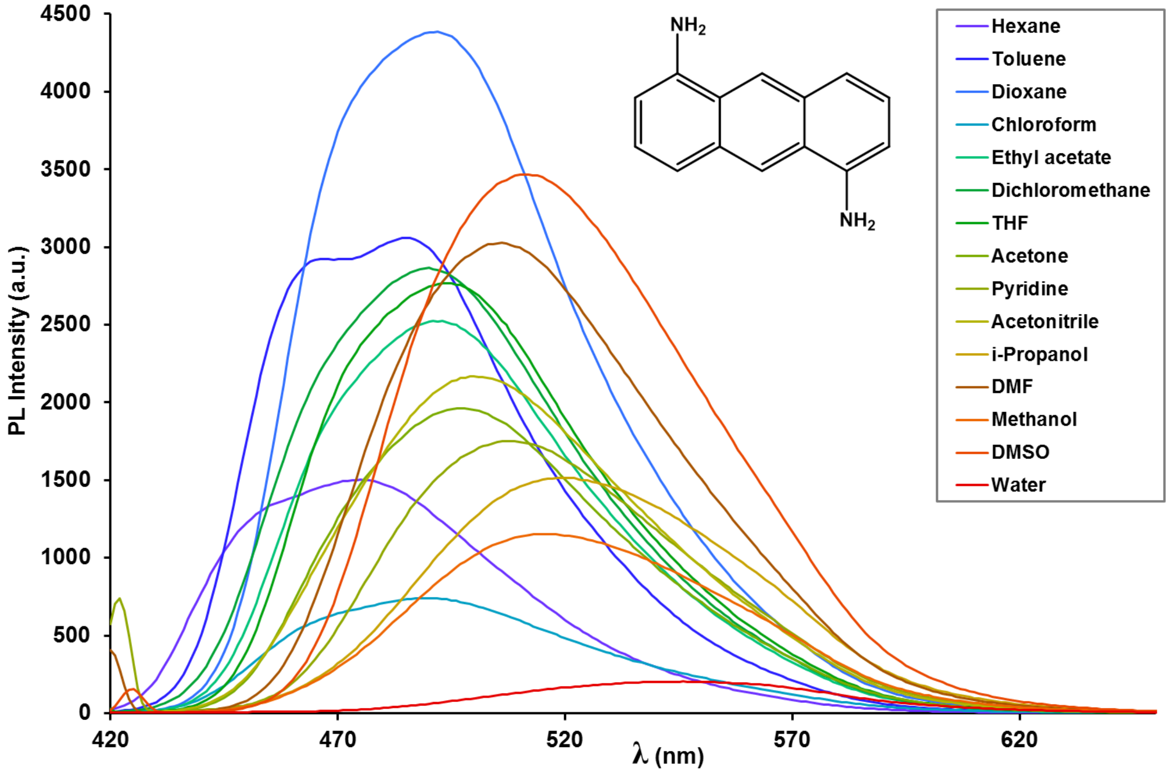

Figure S1. The emission spectra of 1,5-diaminoanthracene (**DAA**) recorded in solvents of different polarity. (c= 5×10^{-5} M, T=20 ºC, V=3.00 ml).

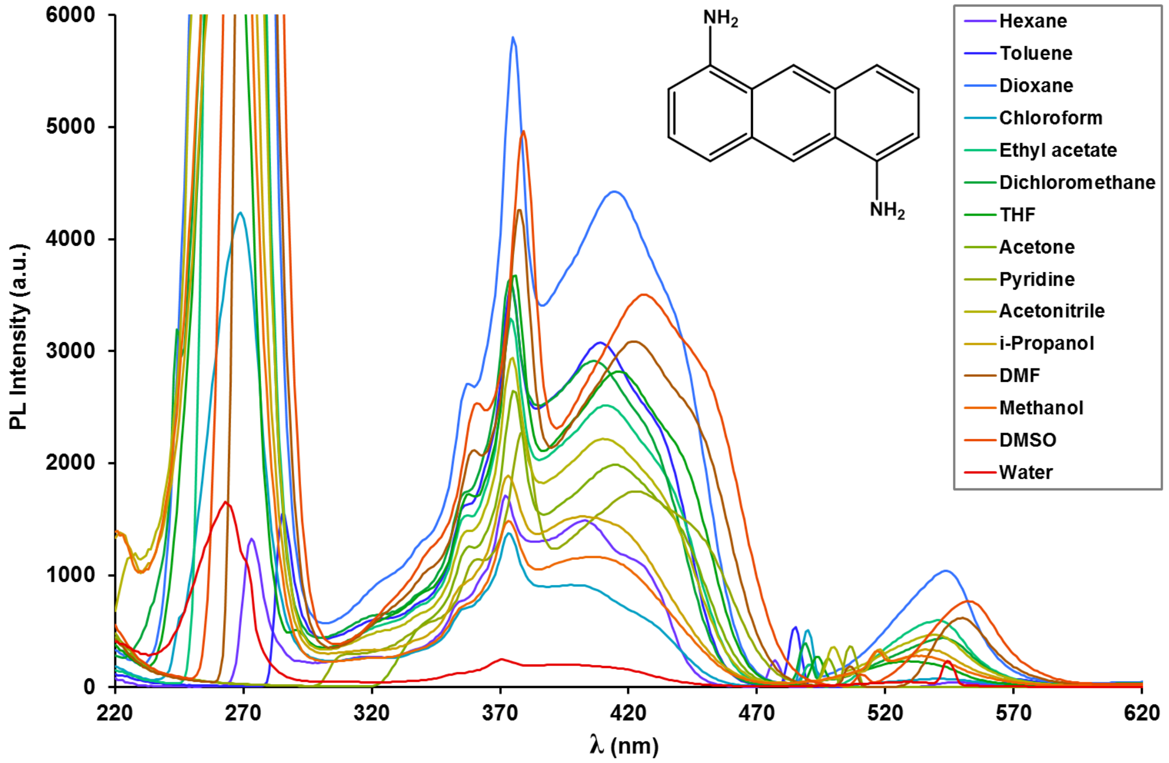

Figure S2. The excitation spectra of 1,5-diaminoanthracene (**DAA**) recorded in solvents of different polarity. (c= 5×10^{-5} M, T=20 ºC, V=3.00 ml).

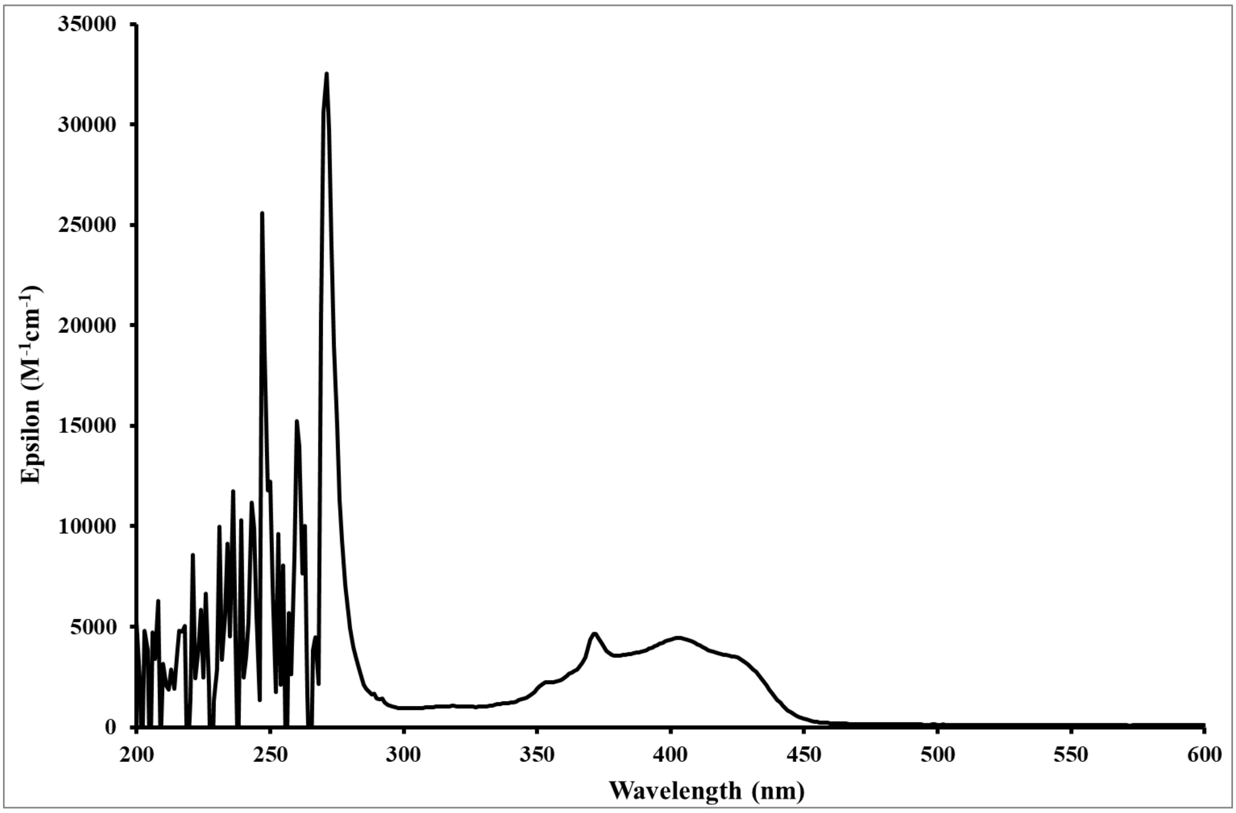

Figure S3. UV-Vis absorbance spectrum of DAA recorded in hexane

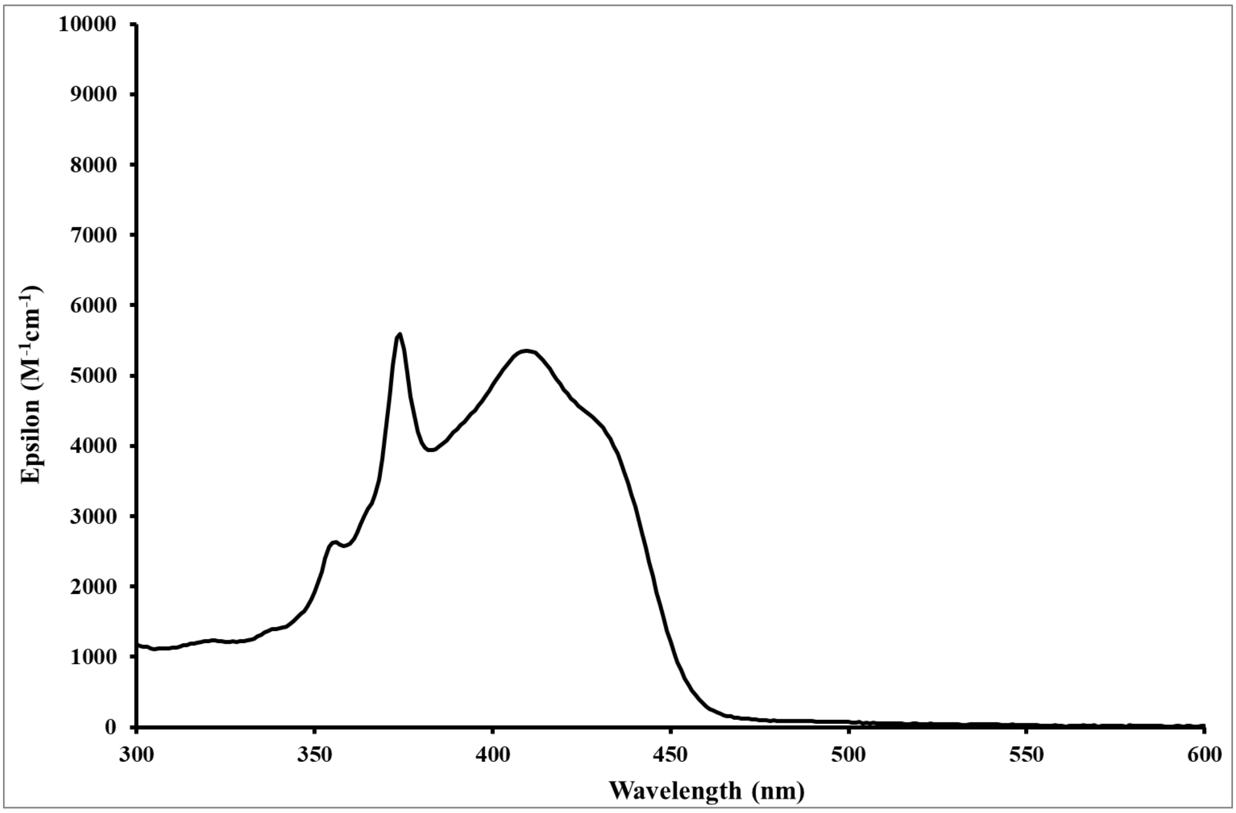

Figure S4. UV-Vis absorbance spectrum of DAA recorded in toluene

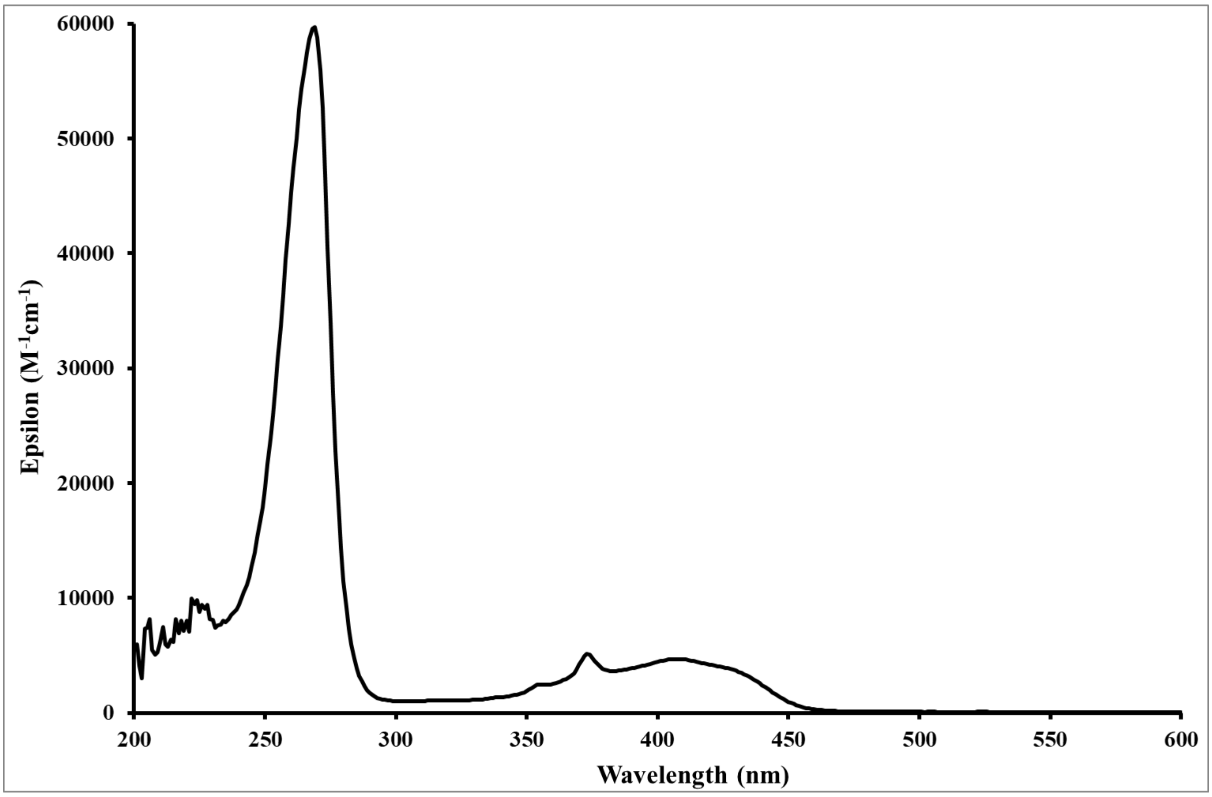

Figure S5. UV-Vis absorbance spectrum of DAA recorded in methylene-chloride

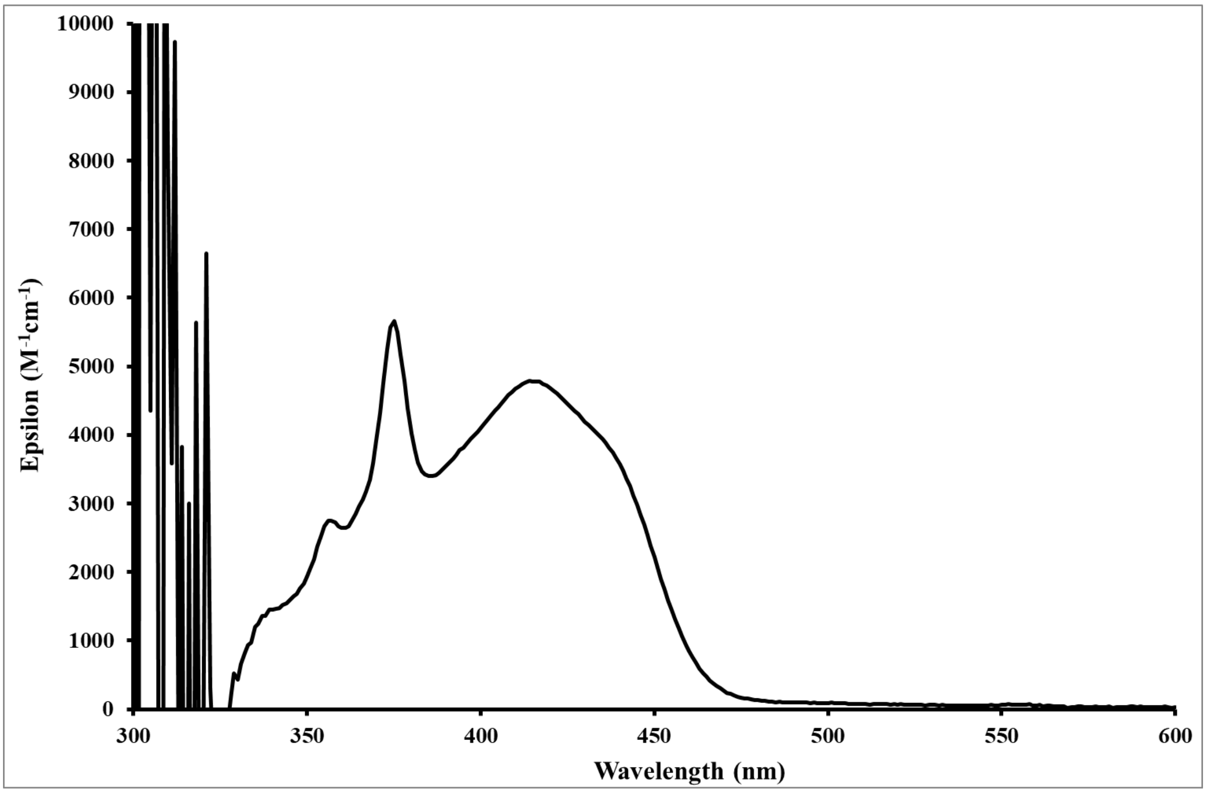

Figure S6. UV-Vis absorbance spectrum of DAA recorded in acetone

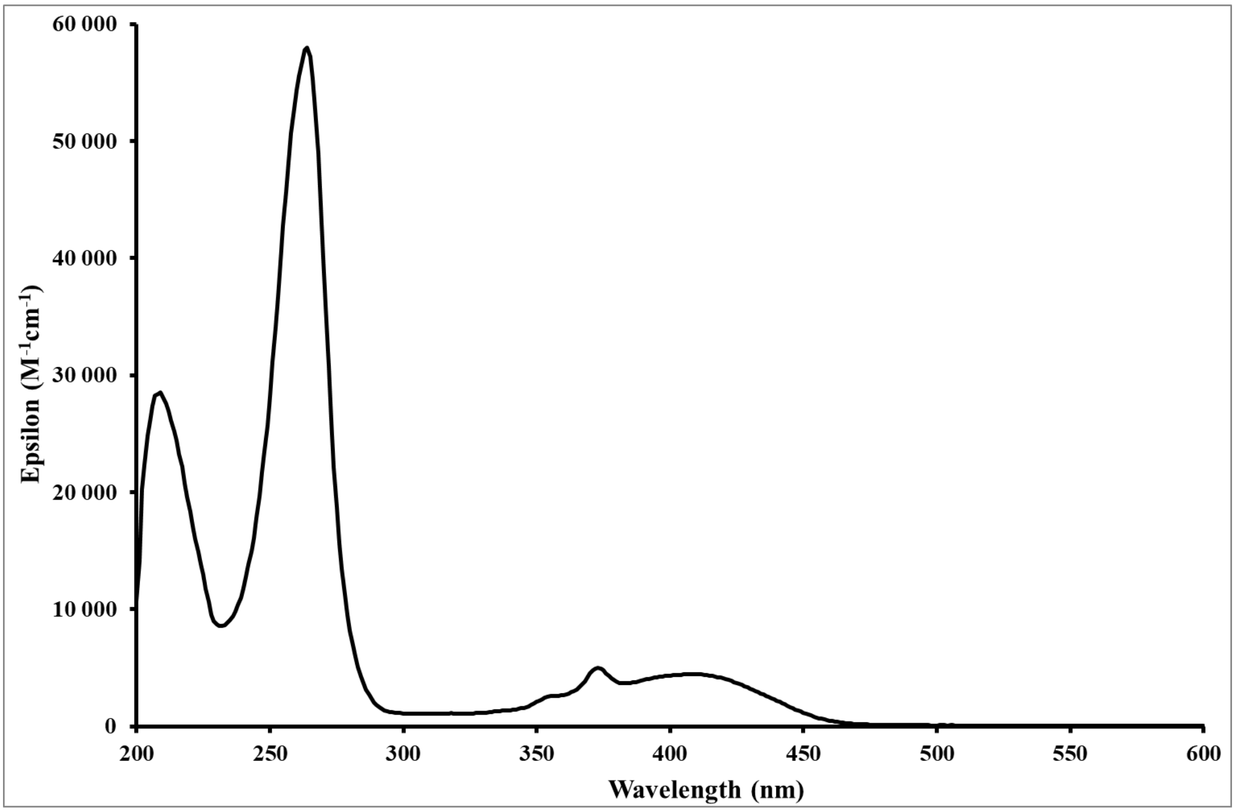

Figure S7. UV-Vis absorbance spectrum of DAA recorded in methanol

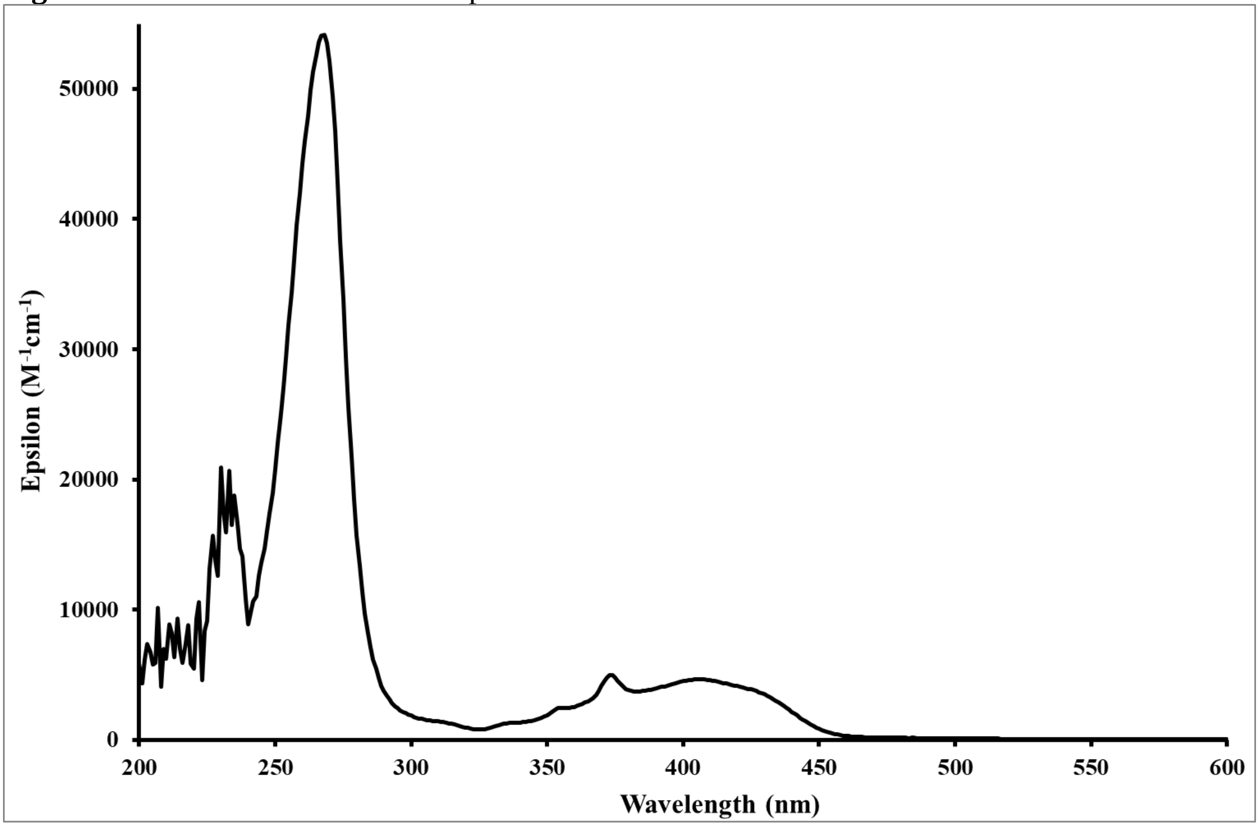

Figure S8. UV-Vis absorbance spectrum of DAA recorded in chloroform

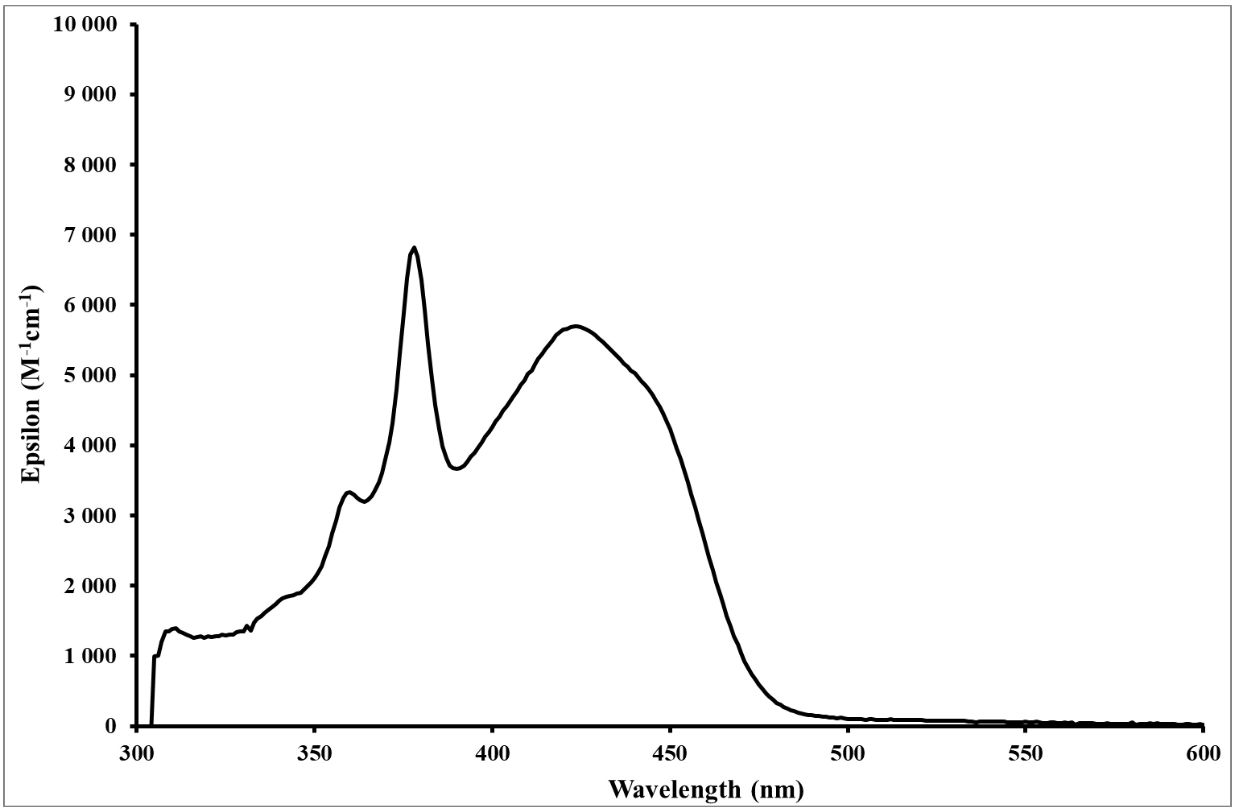

Figure S9. UV-Vis absorbance spectrum of DAA recorded in pyridine

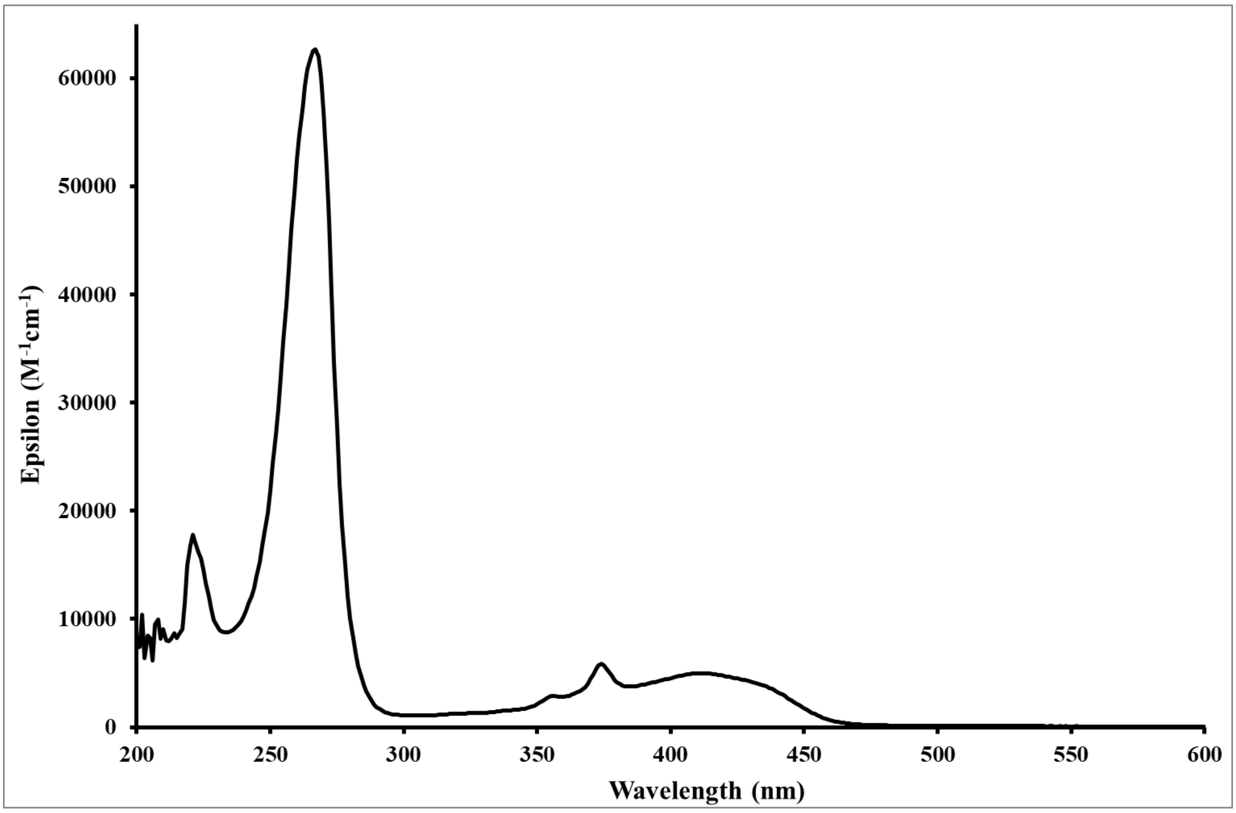

Figure S10. UV-Vis absorbance spectrum of DAA recorded in acetonitrile

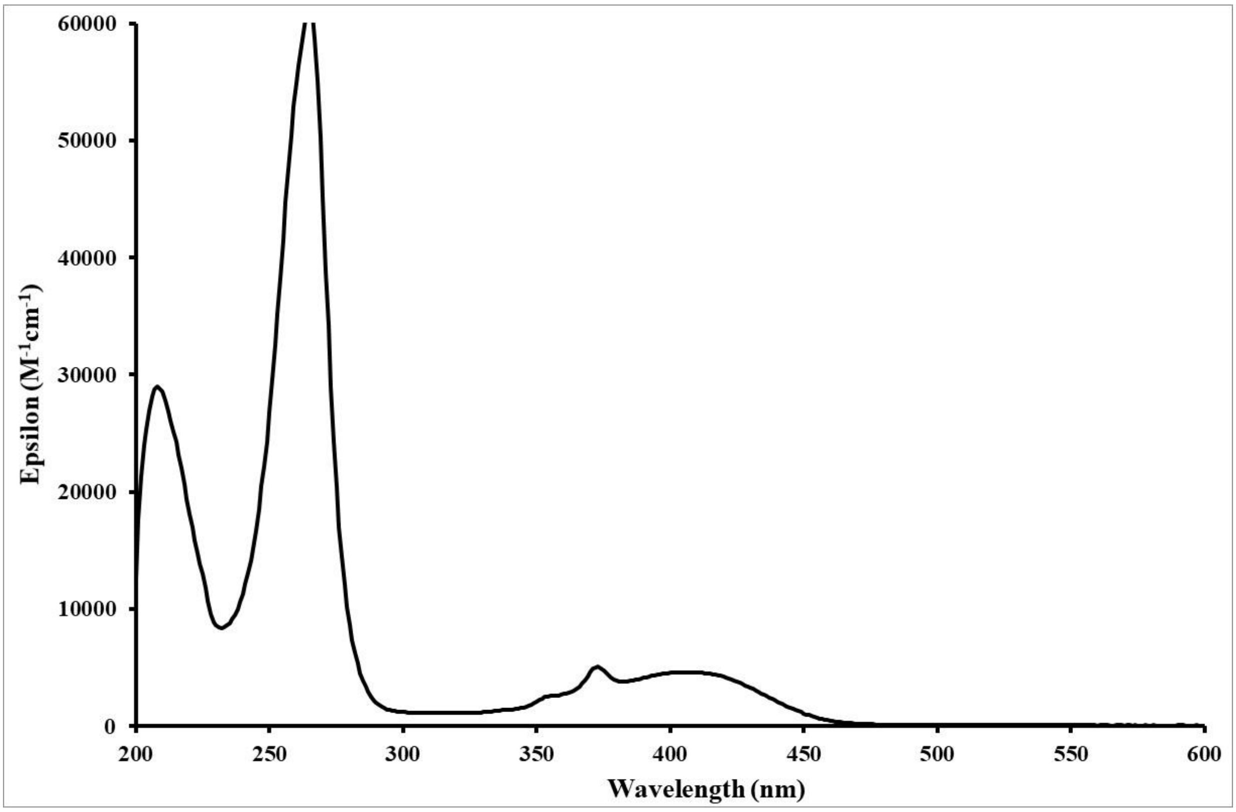

Figure S11. UV-Vis absorbance spectrum of DAA recorded in 2-propanol

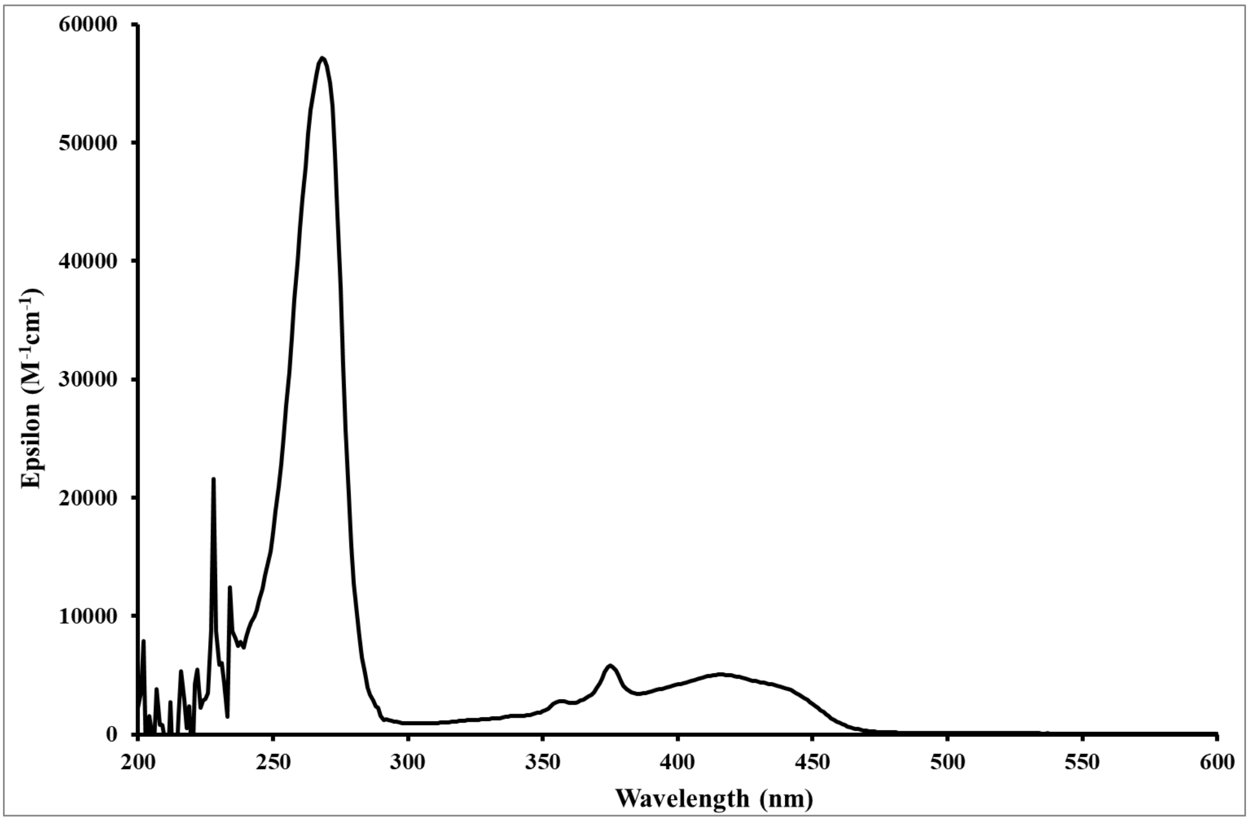

Figure S12. UV-Vis absorbance spectrum of DAA recorded in tetrahydrofuran

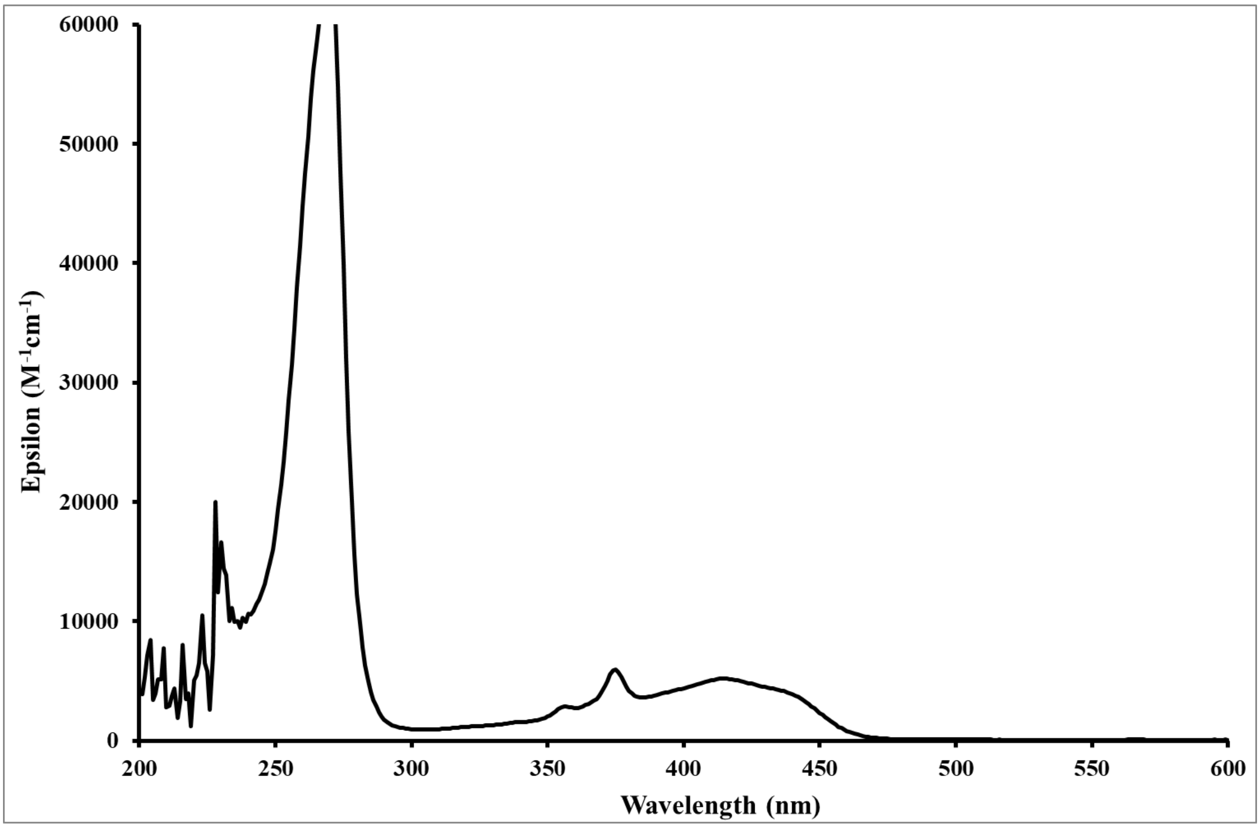

Figure S13. UV-Vis absorbance spectrum of DAA recorded in dioxane

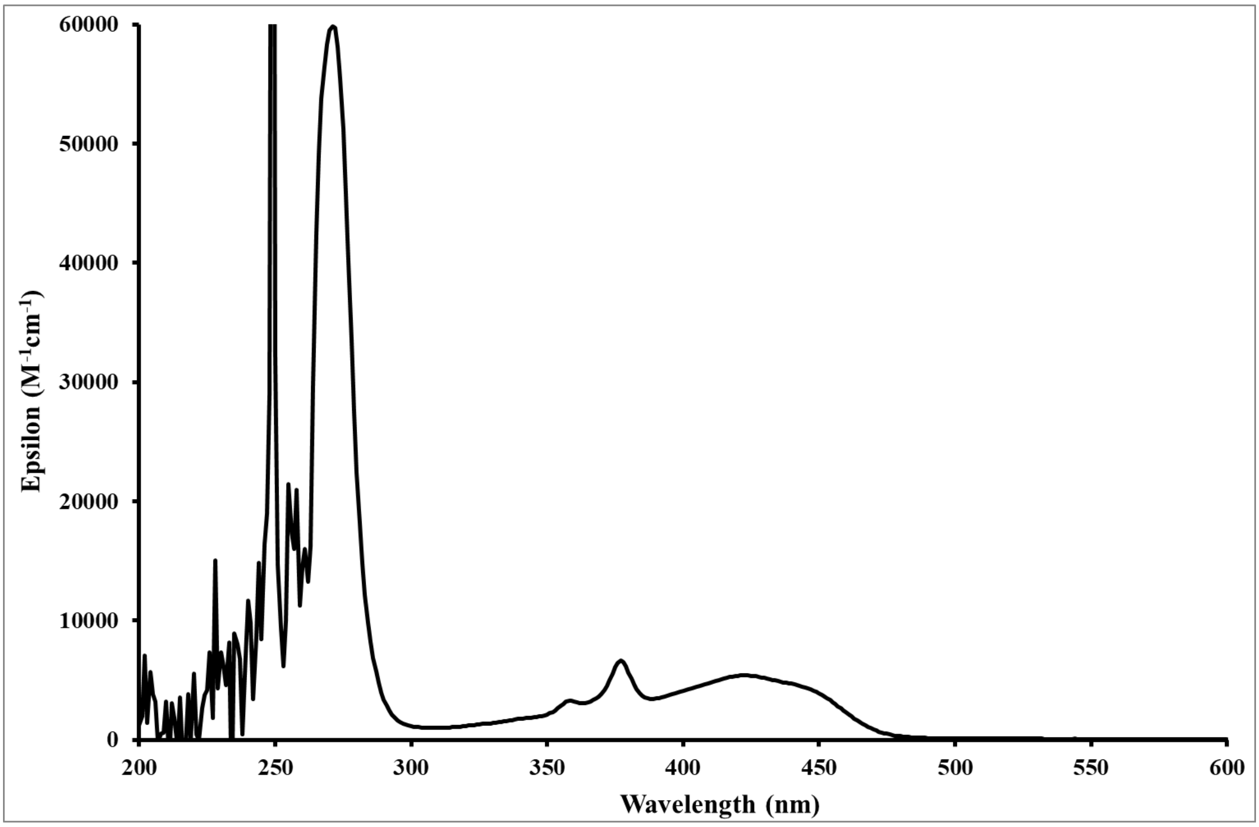

Figure S14. UV-Vis absorbance spectrum of DAA recorded in dimethylformamide

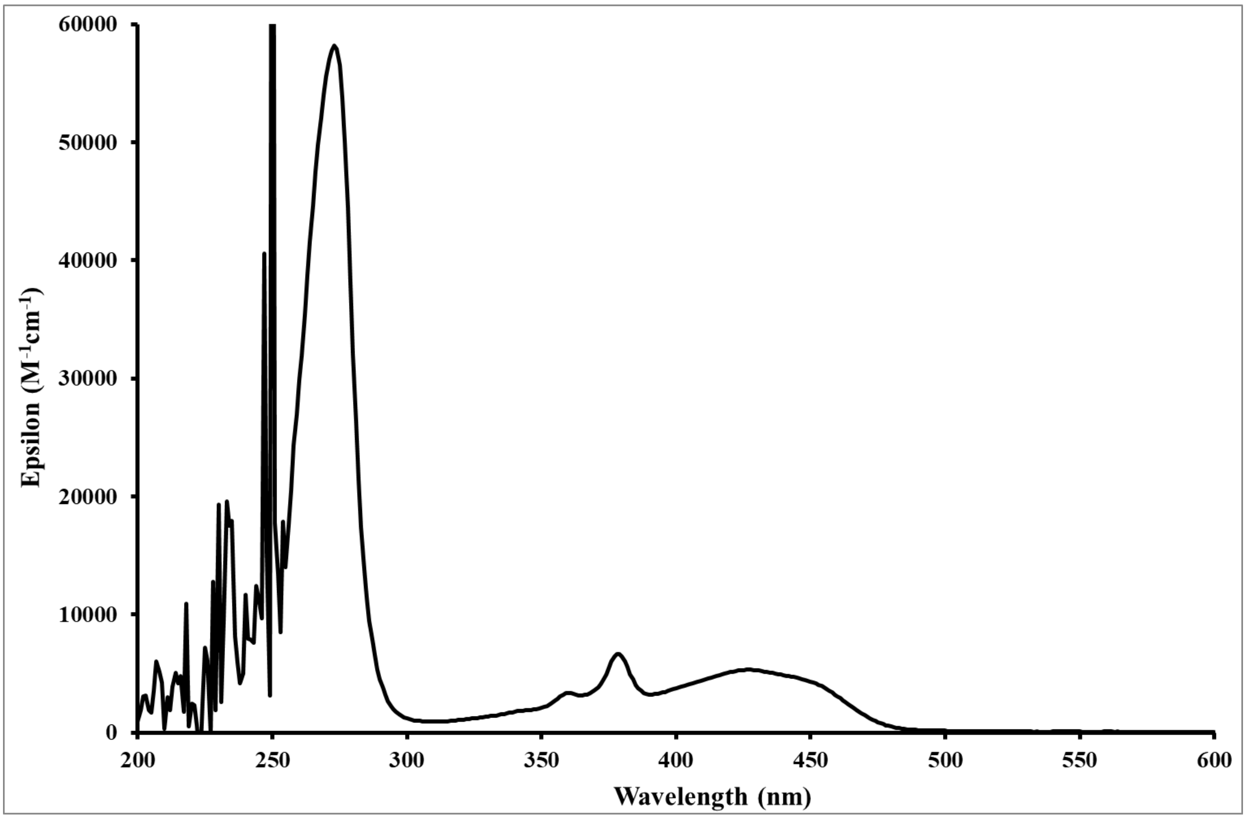

Figure S15. UV-Vis absorbance spectrum of DAA recorded in dimethyl sulfoxide

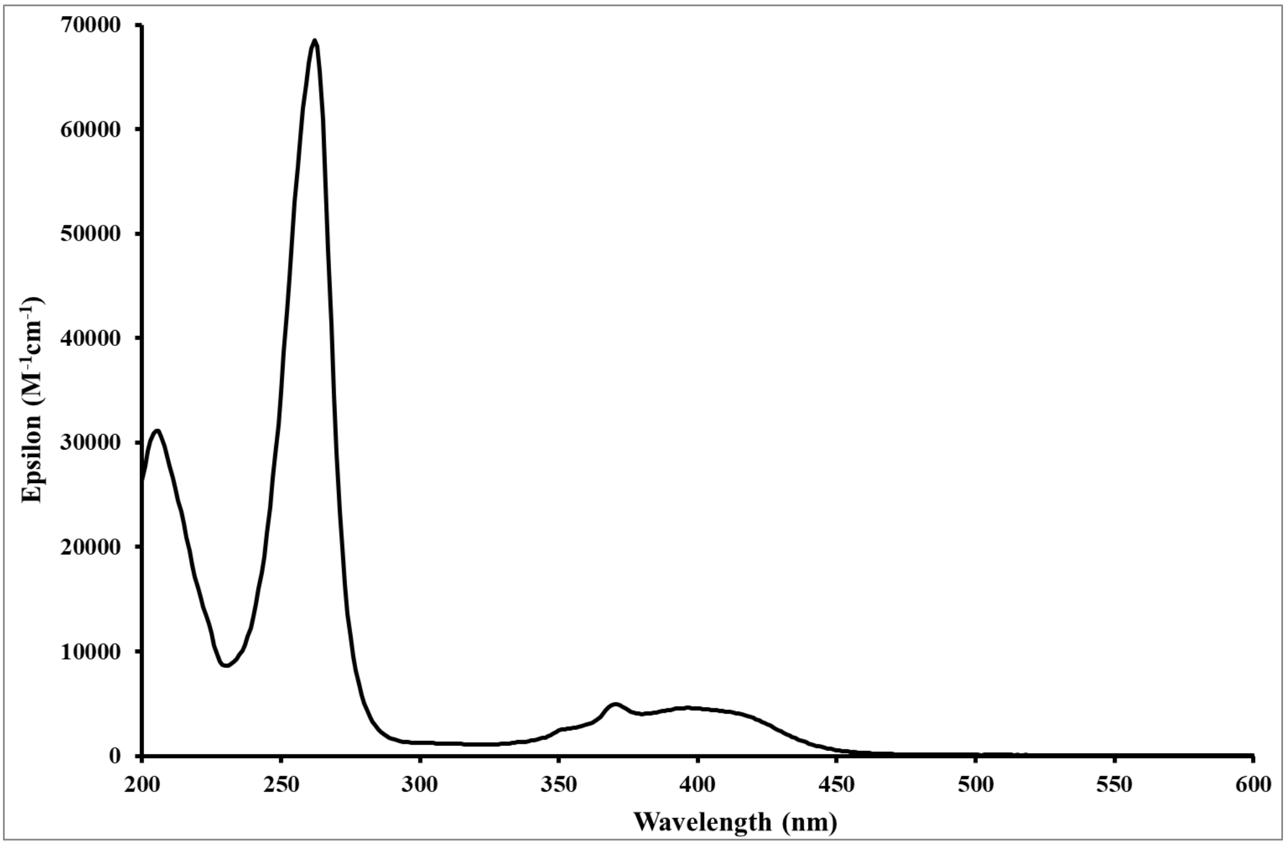

Figure S16. UV-Vis absorbance spectrum of DAA recorded in water

Chapter 2. 1-amino-5-isocyanoanthracene (ICAA)

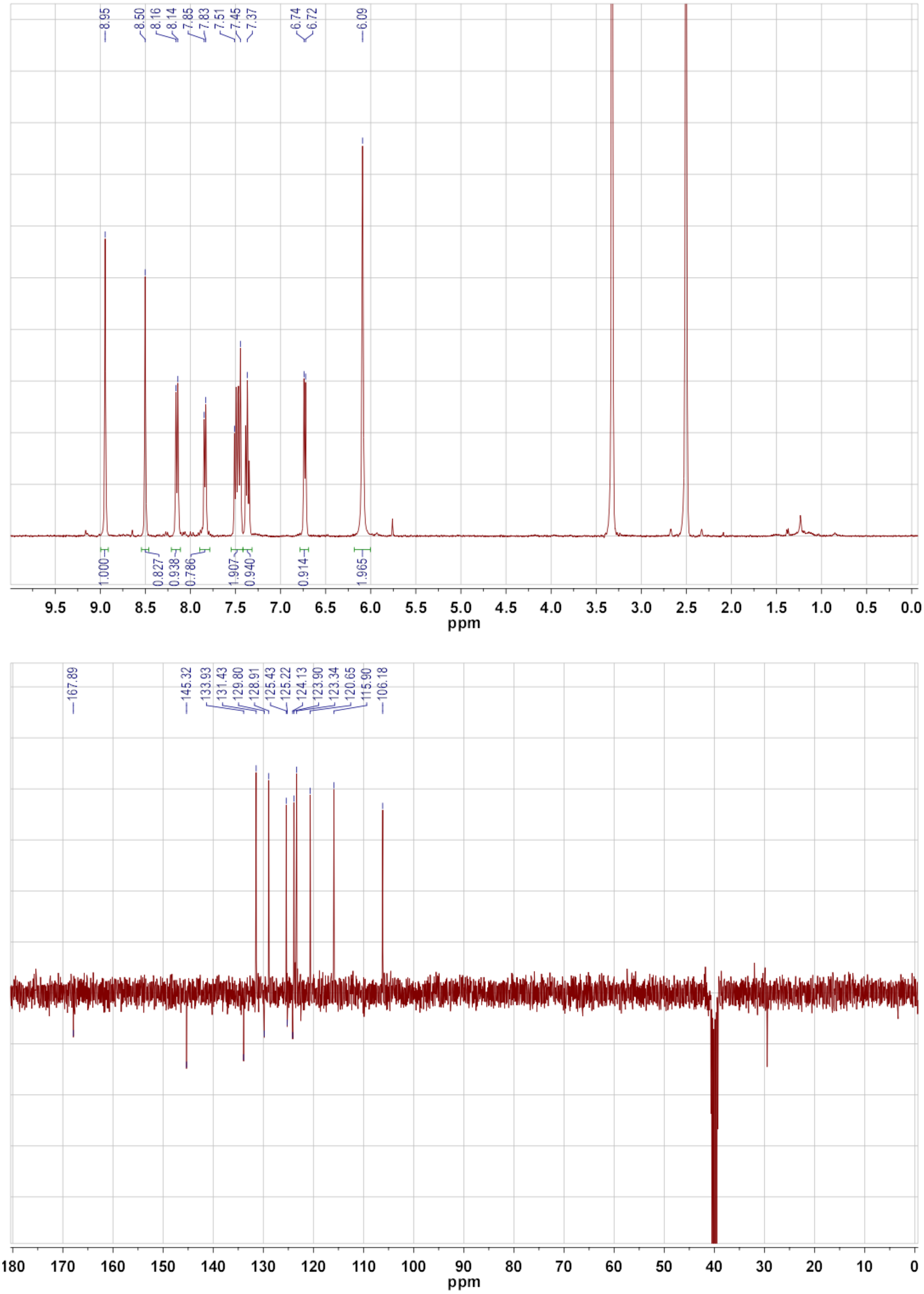

Figure S17. ^{1}H-NMR (top) and ^{13}C-NMR (bottom) spectra of ICAA recorded at 20 °C in DMSO-d_6

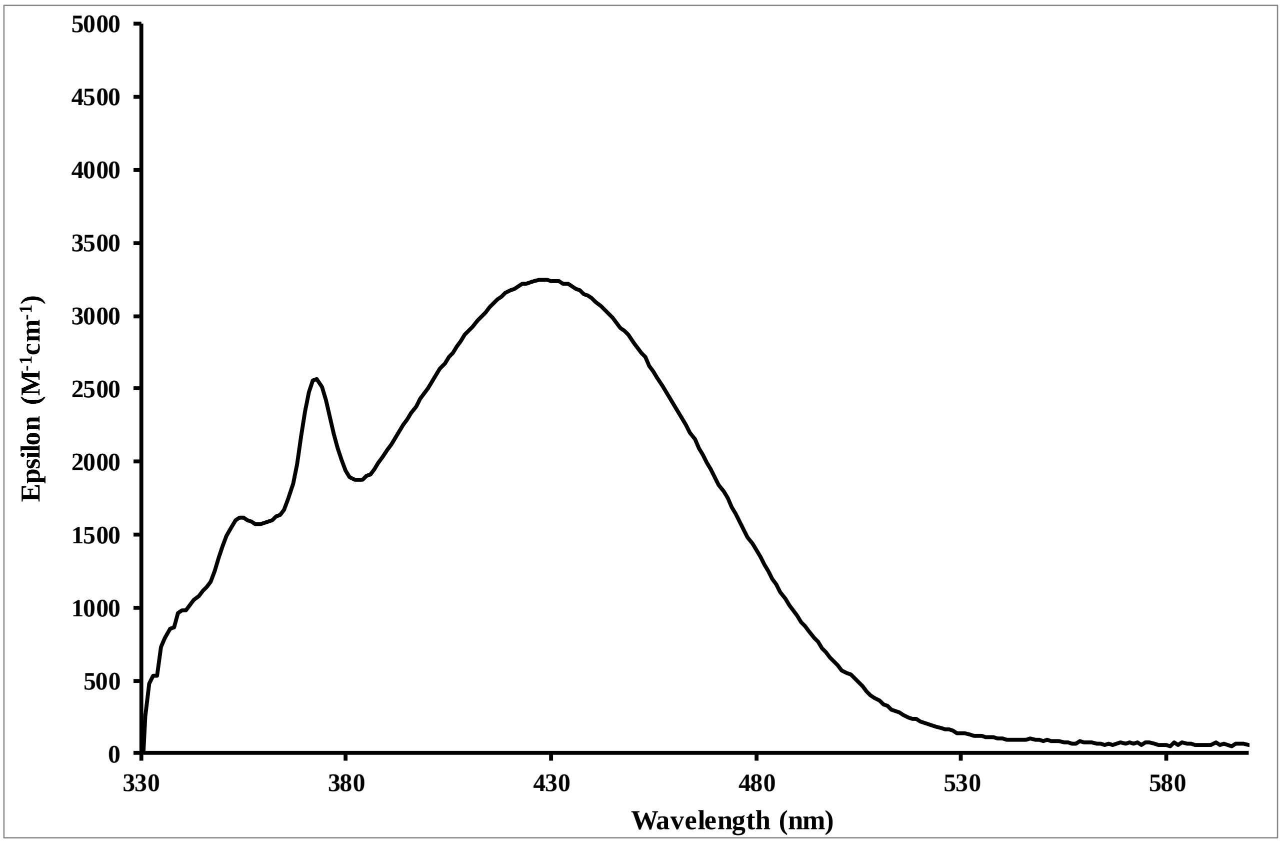

Figure S18. UV-Vis absorbance spectrum of ICAA recorded in acetone

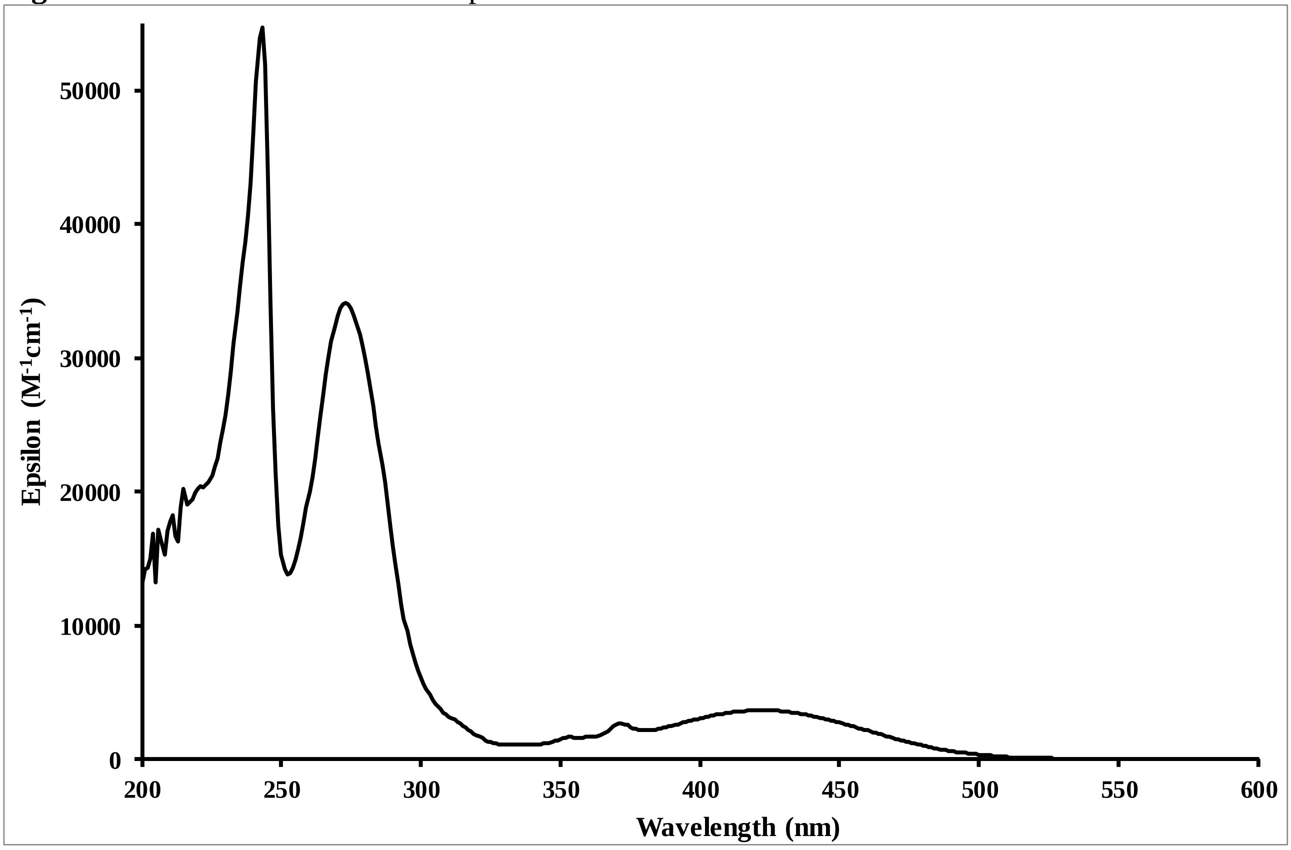

Figure S19. UV-Vis absorbance spectrum of ICAA recorded in acetonitrile

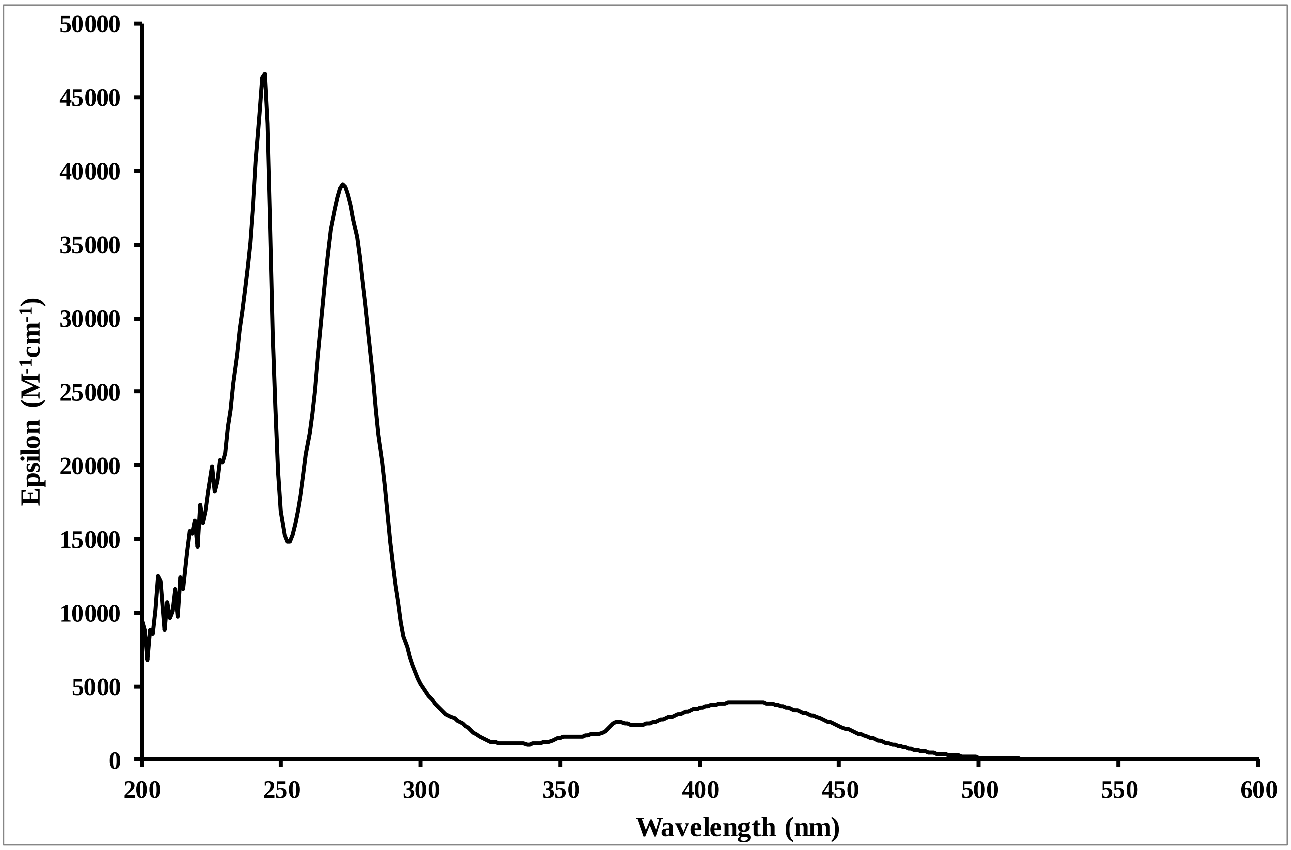

Figure S20. UV-Vis absorbance spectrum of ICAA recorded in dichloromethane

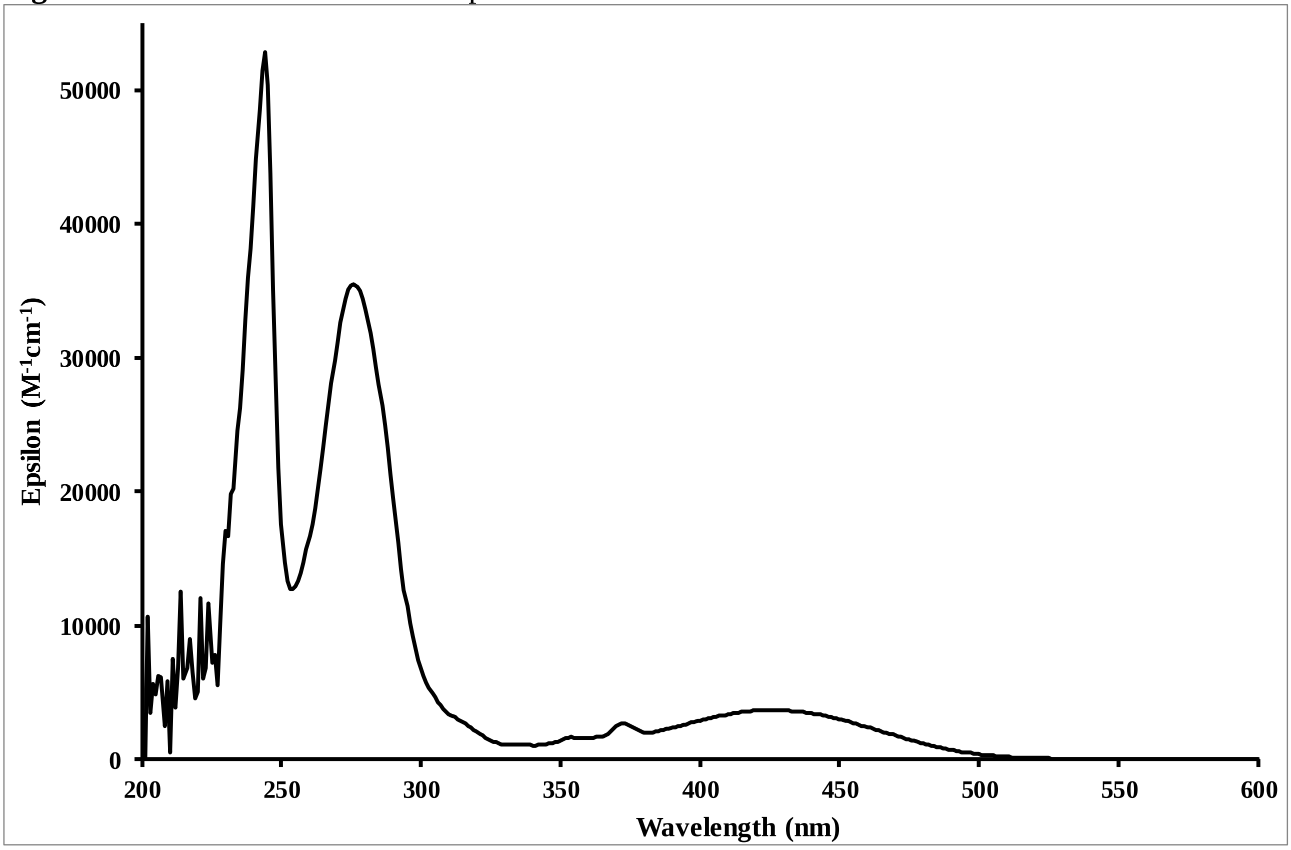

Figure S21. UV-Vis absorbance spectrum of ICAA recorded in 1,4-dioxane

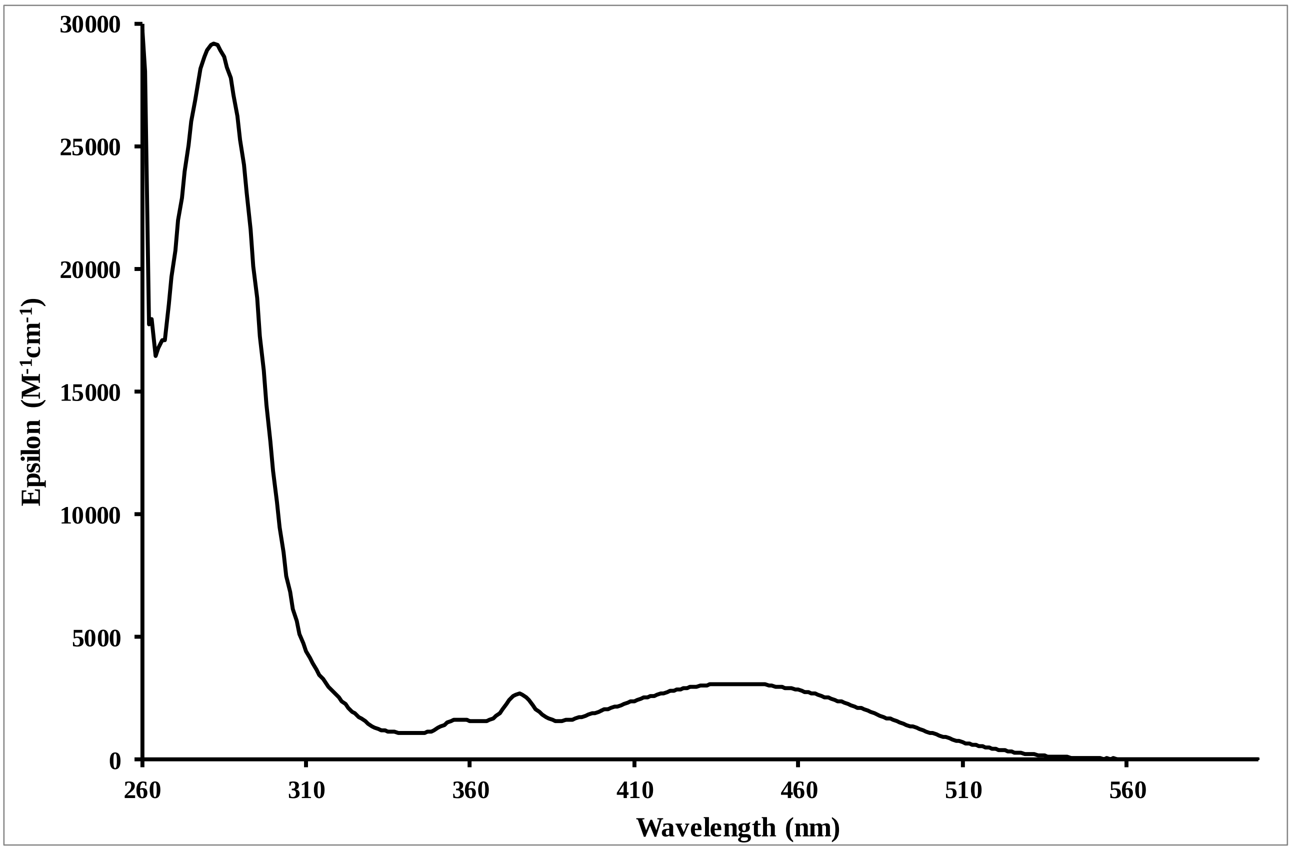

Figure S22. UV-Vis absorbance spectrum of ICAA recorded in DMF

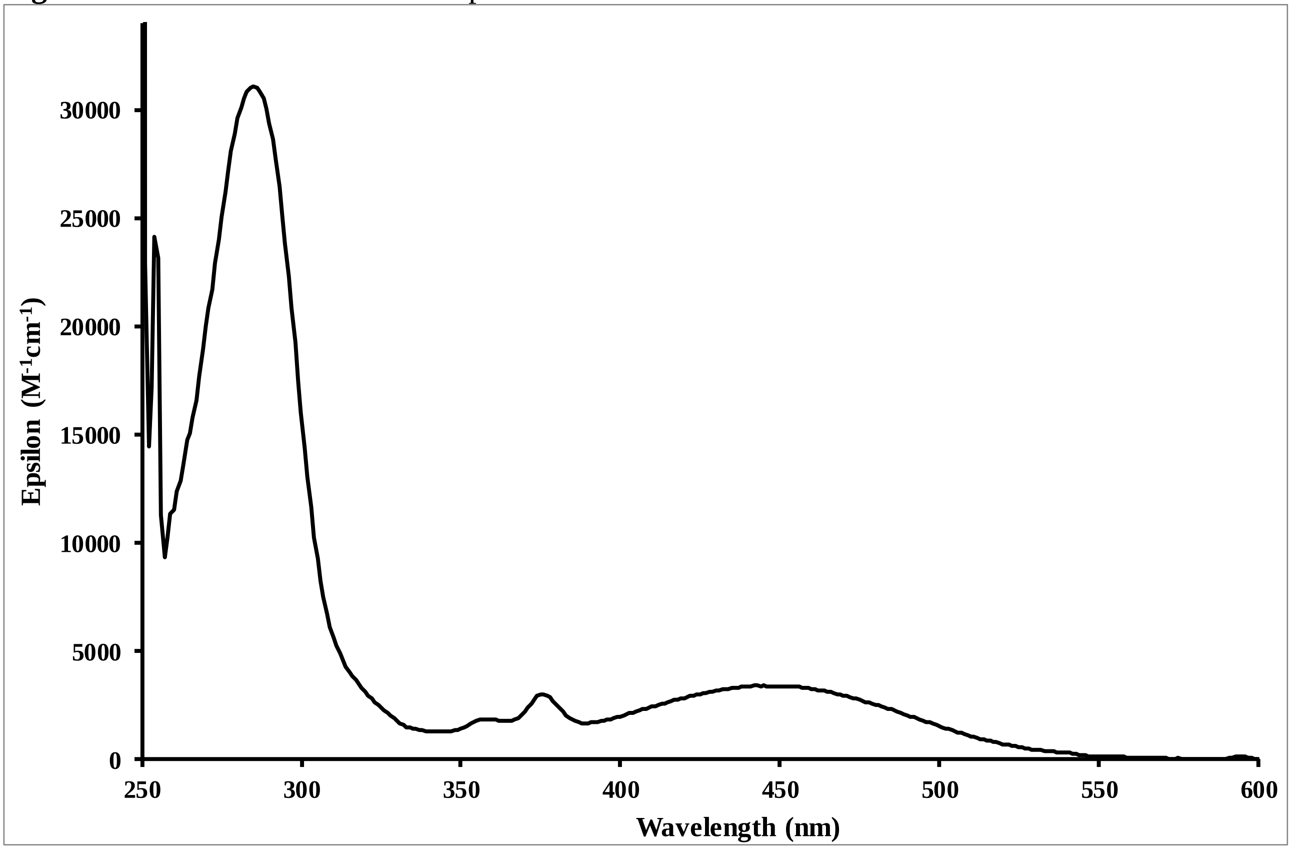

Figure S23. UV-Vis absorbance spectrum of ICAA recorded in DMSO

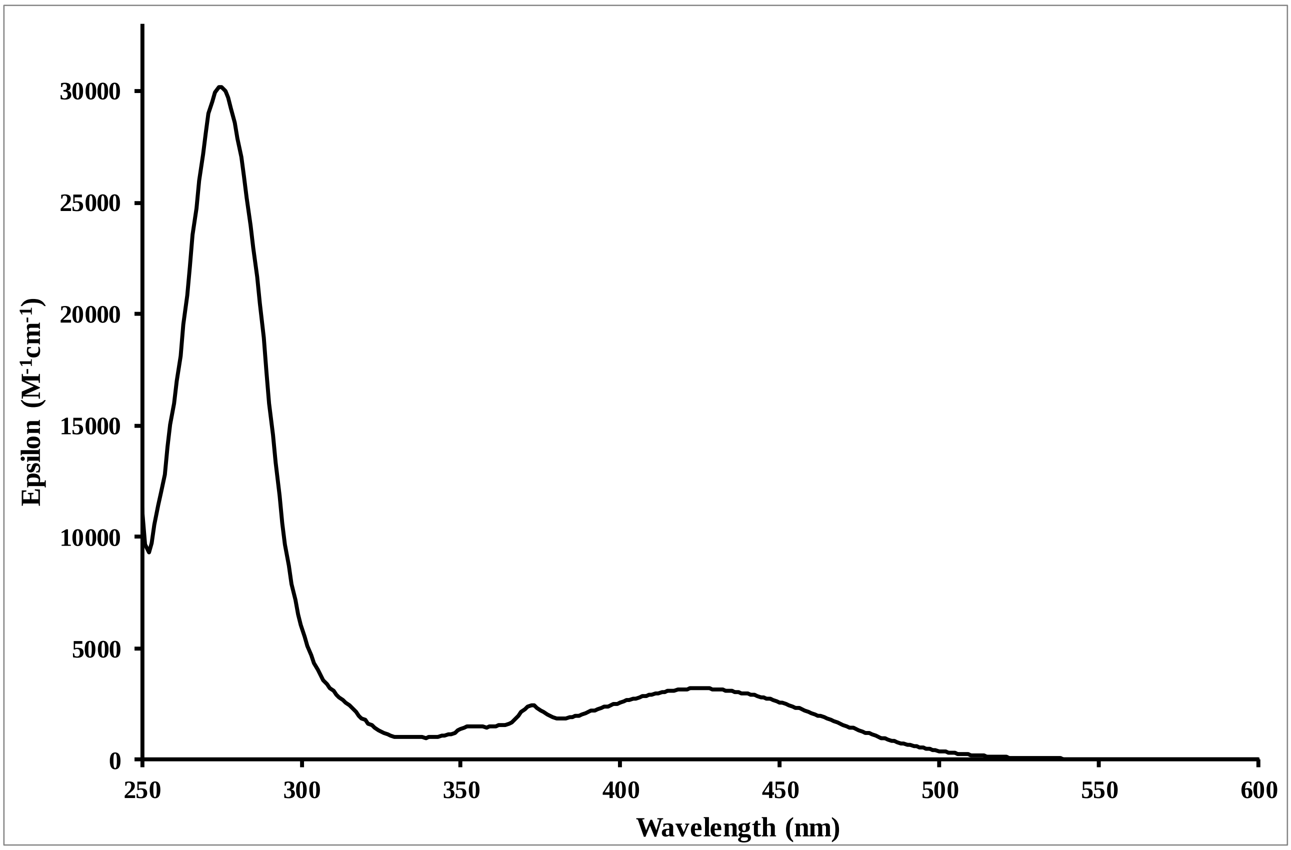

Figure S24. UV-Vis absorbance spectrum of ICAA recorded in ethyl acetate

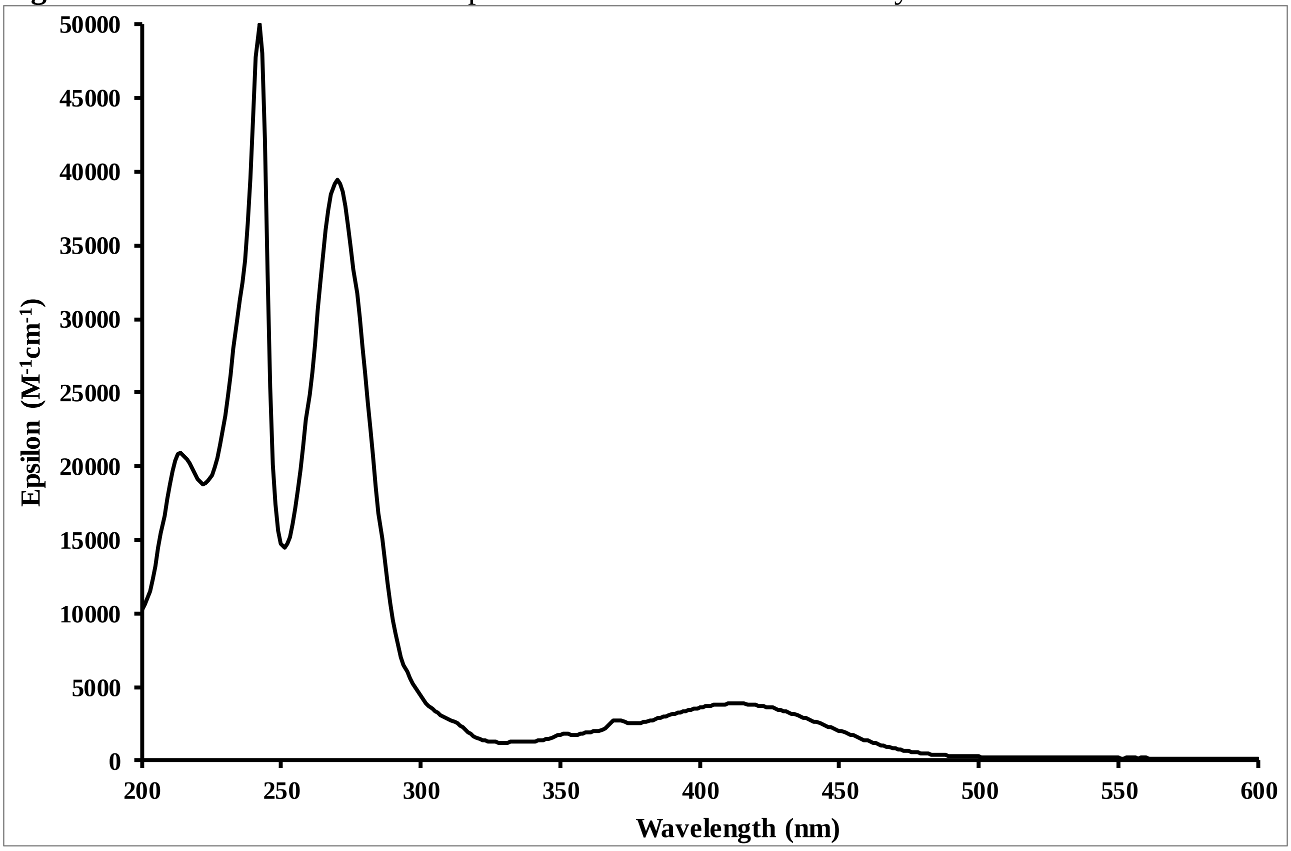

Figure S25. UV-Vis absorbance spectrum of ICAA recorded in hexane

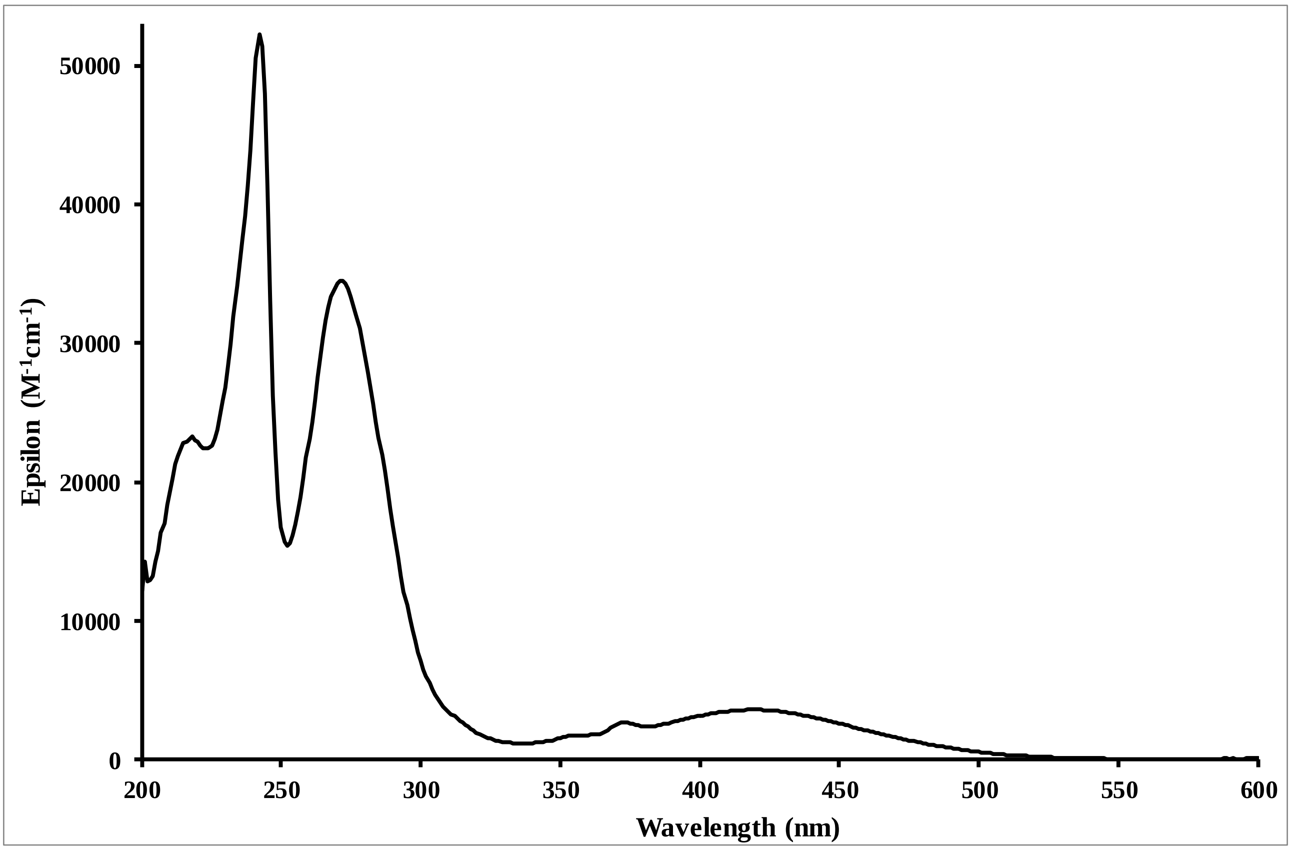

Figure S26. UV-Vis absorbance spectrum of ICAA recorded in 2-propanol

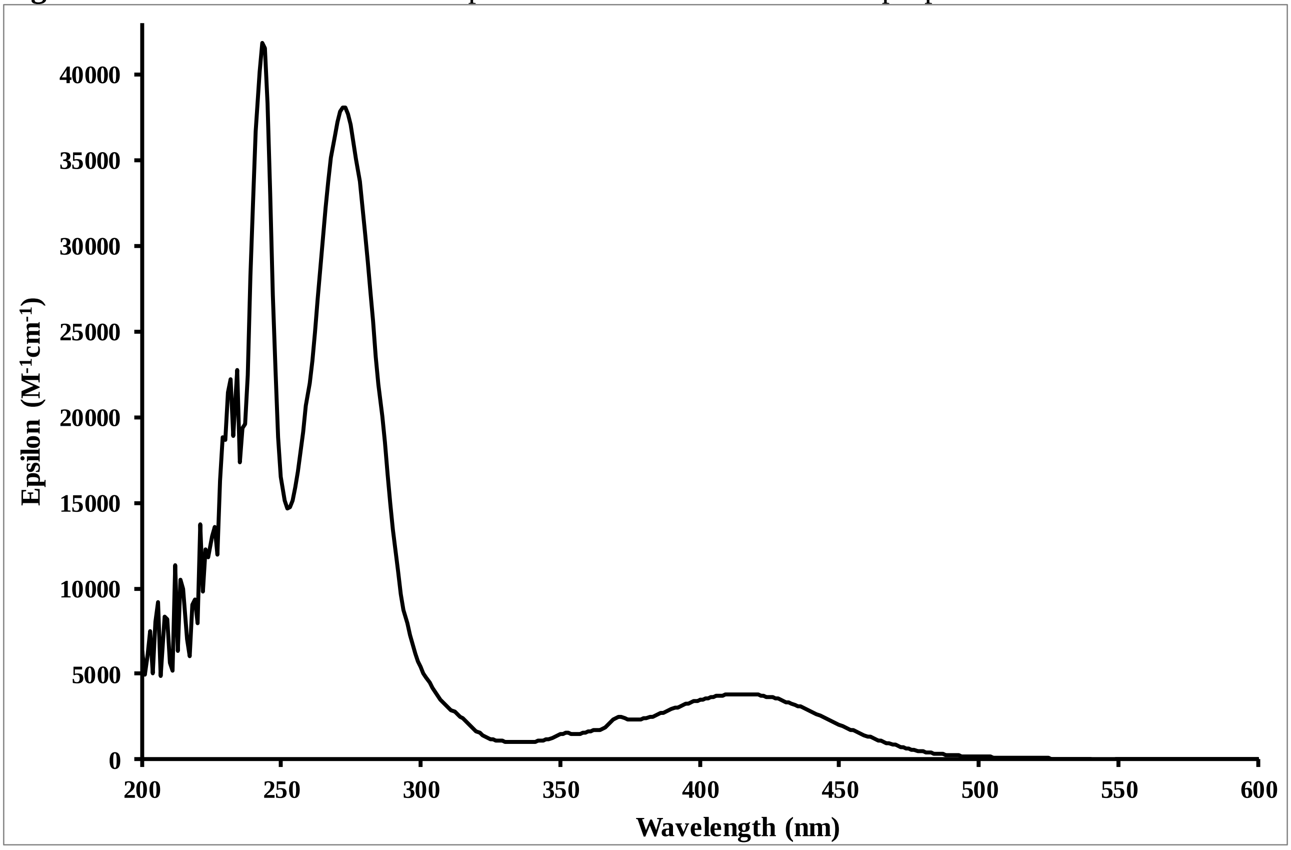

Figure S27. UV-Vis absorbance spectrum of ICAA recorded in chloroform

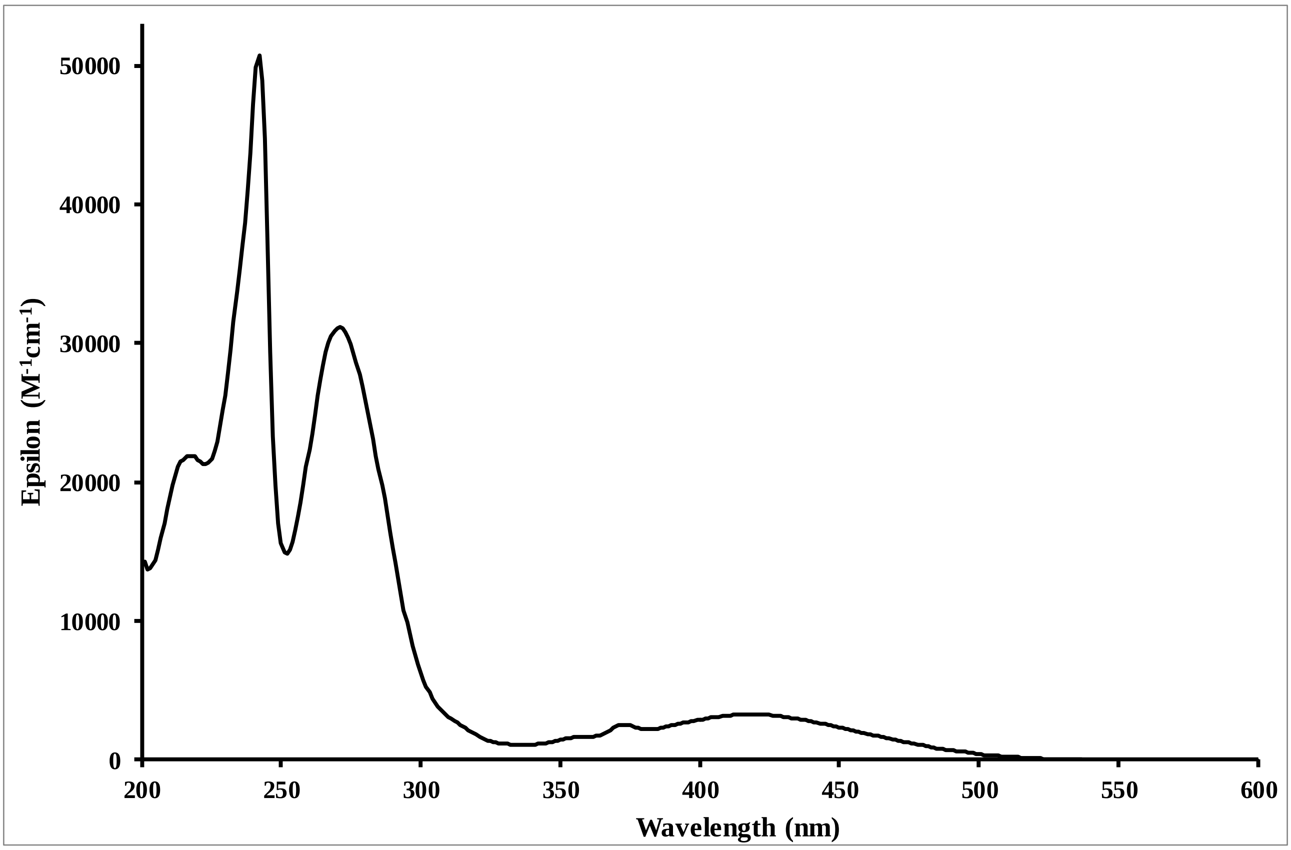

Figure S28. UV-Vis absorbance spectrum of ICAA recorded in methanol

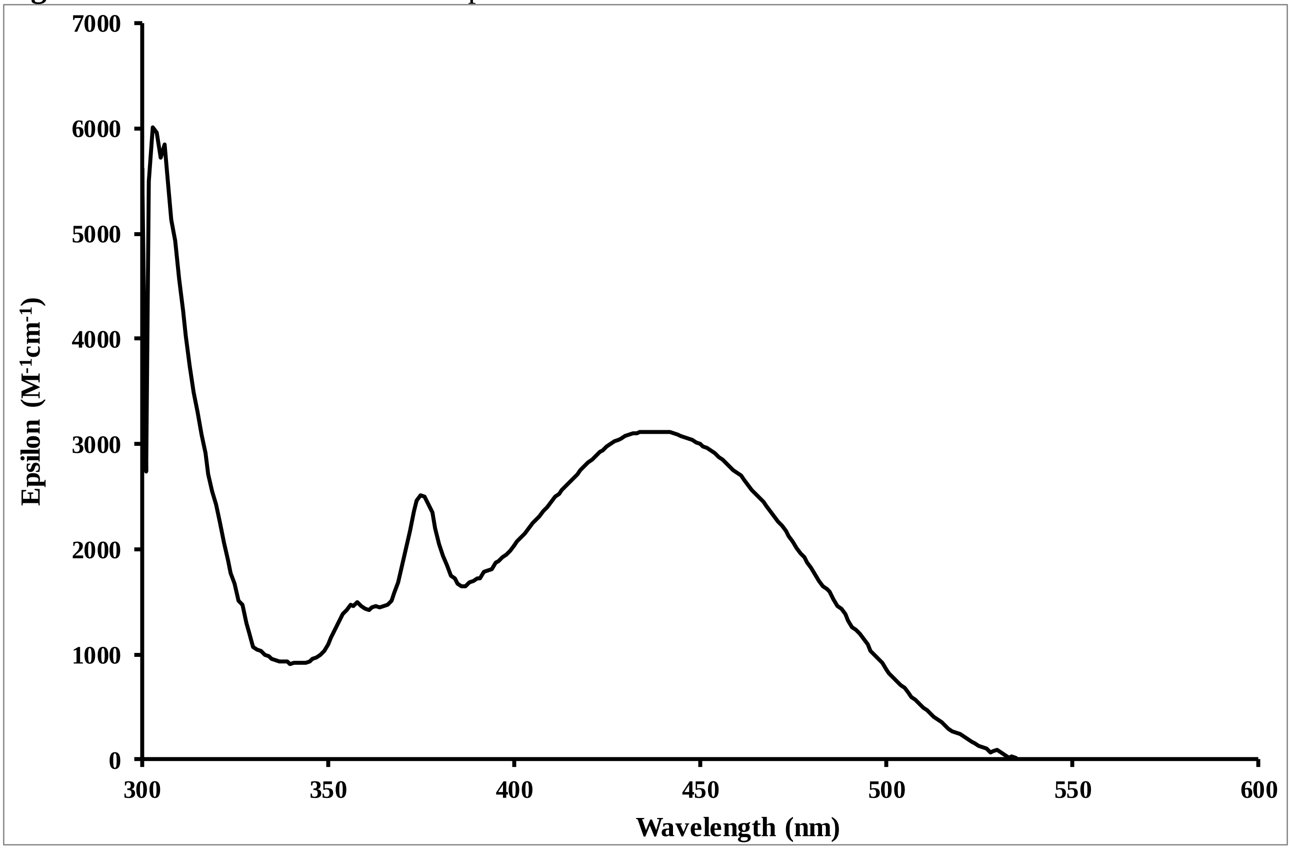

Figure S29. UV-Vis absorbance spectrum of ICAA recorded in pyridine

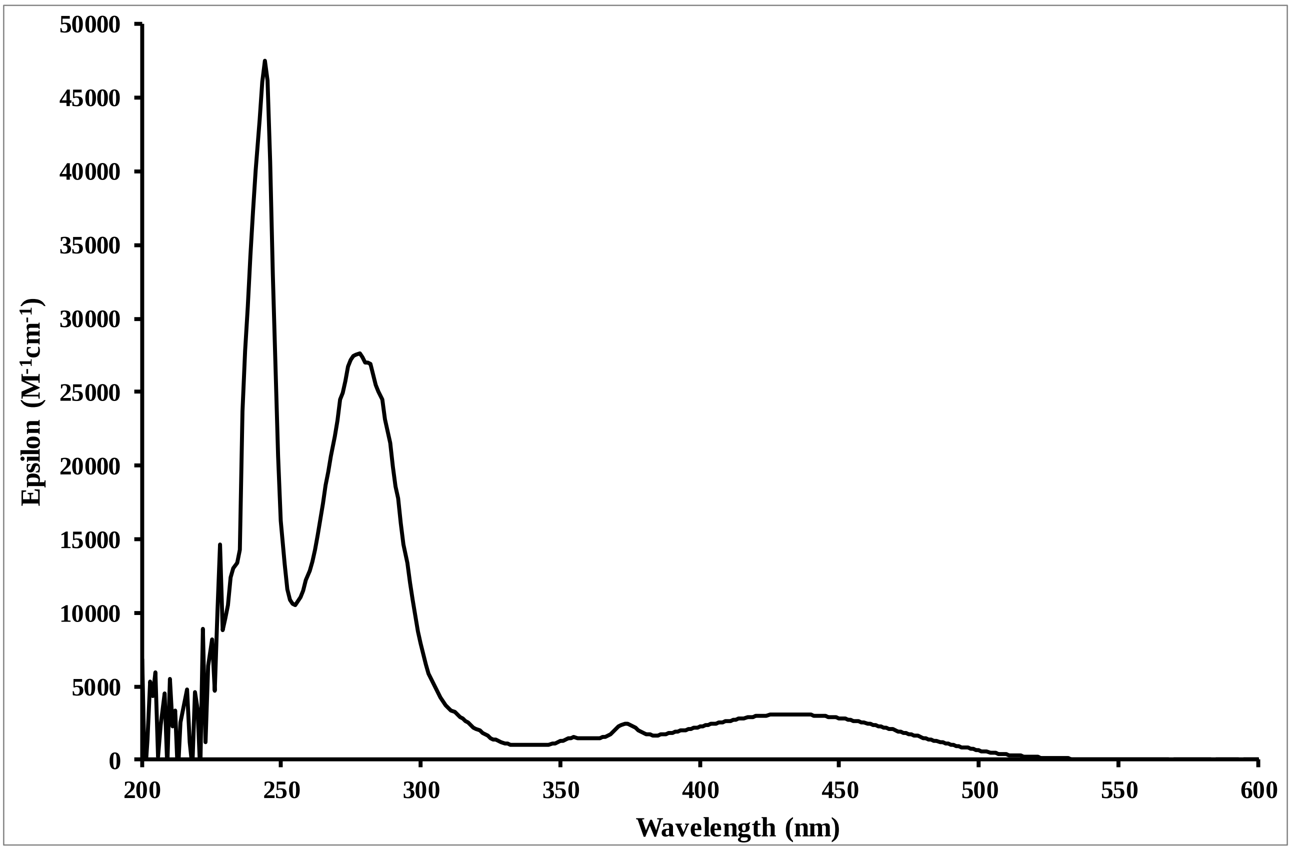

Figure S30. UV-Vis absorbance spectrum of ICAA recorded in THF

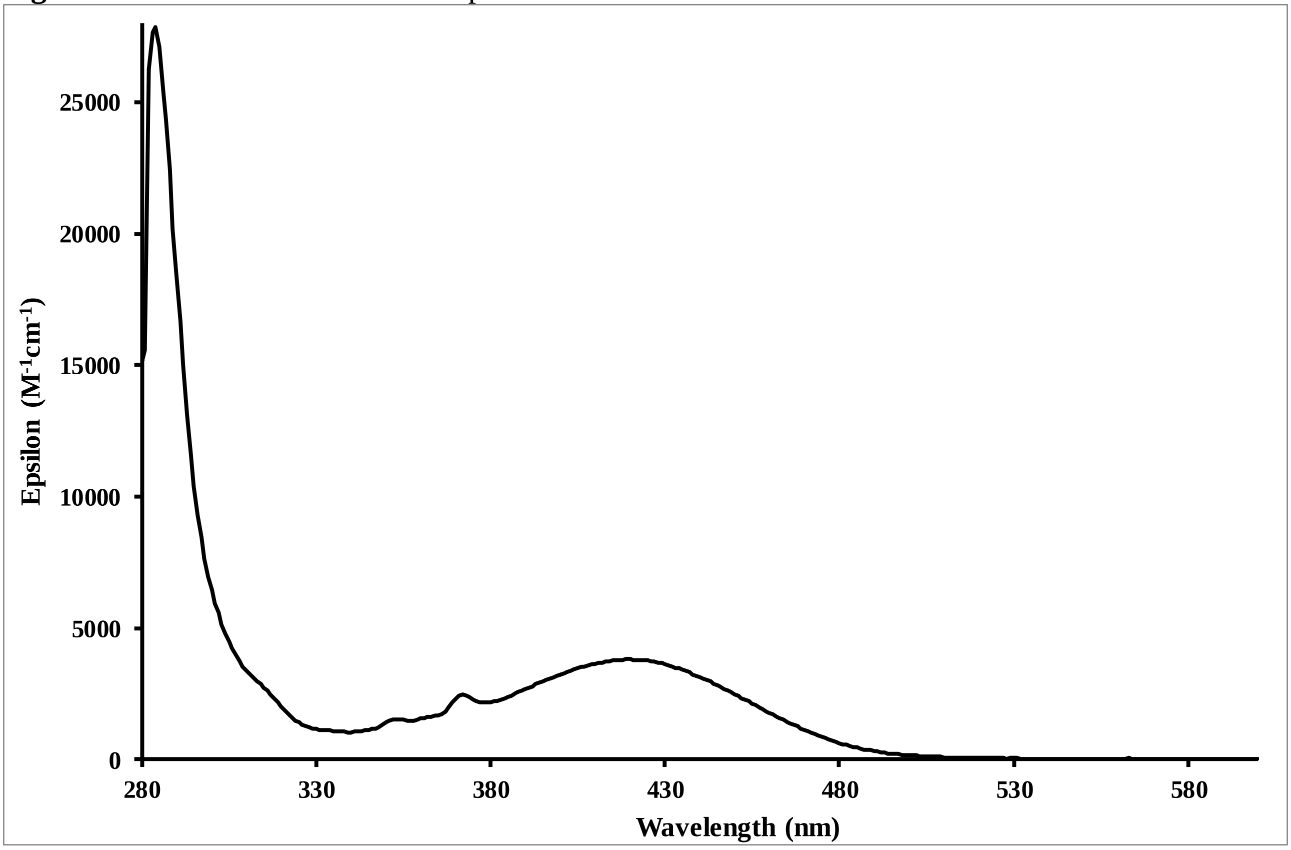

Figure S31. UV-Vis absorbance spectrum of ICAA recorded in toluene

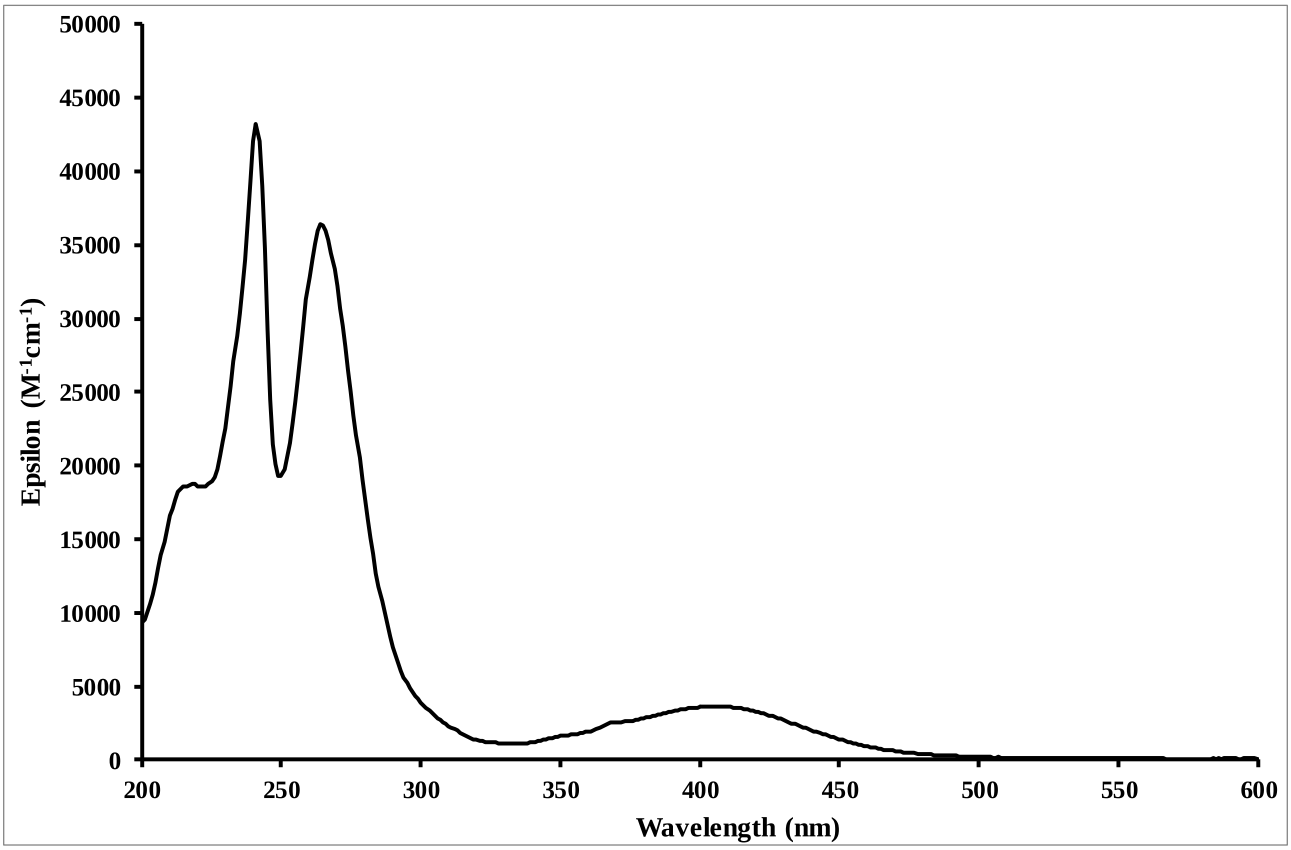

Figure S32. UV-Vis absorbance spectrum of ICAA recorded in water

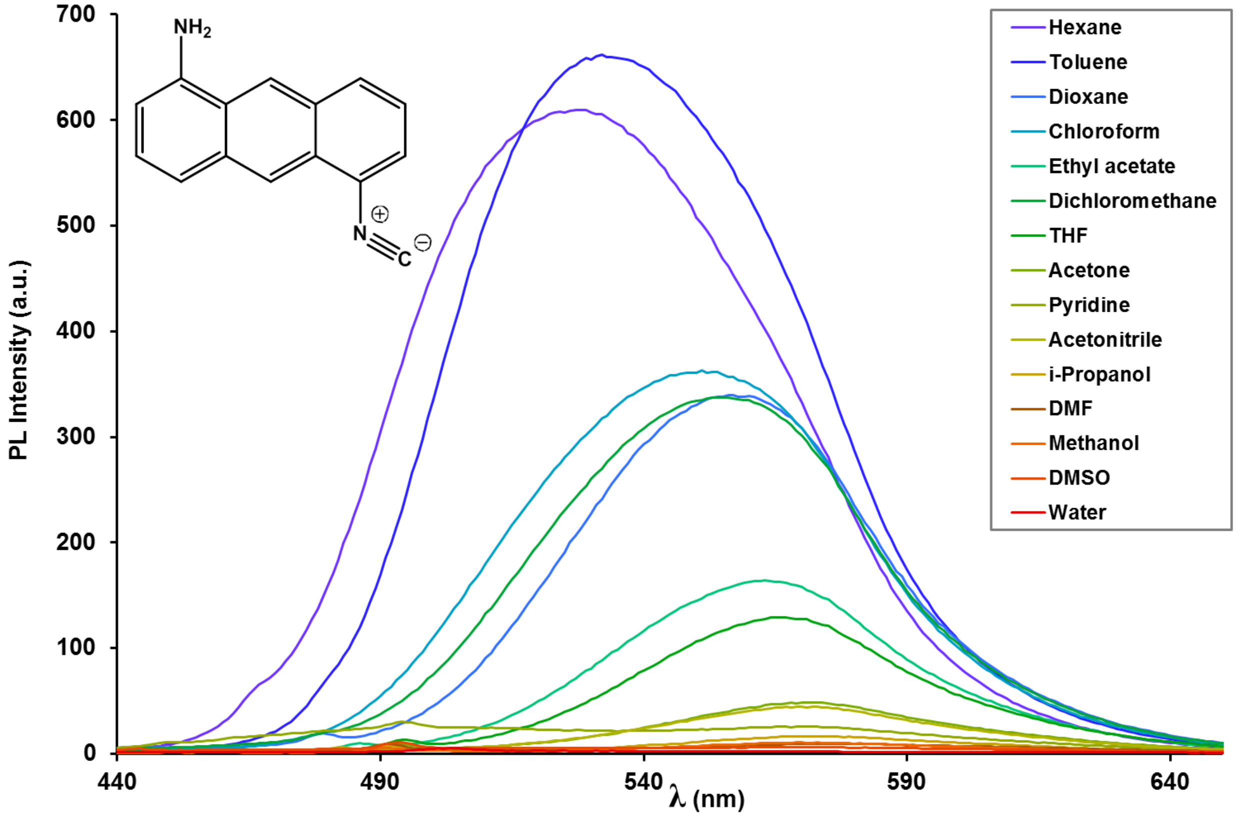

Figure S33. The emission spectra of 1-amino-5-isocyanoanthracene (**ICAA**) recorded in solvents of different polarity. (c= 5×10^{-5} M, T=20 ºC, V=3.00 ml).

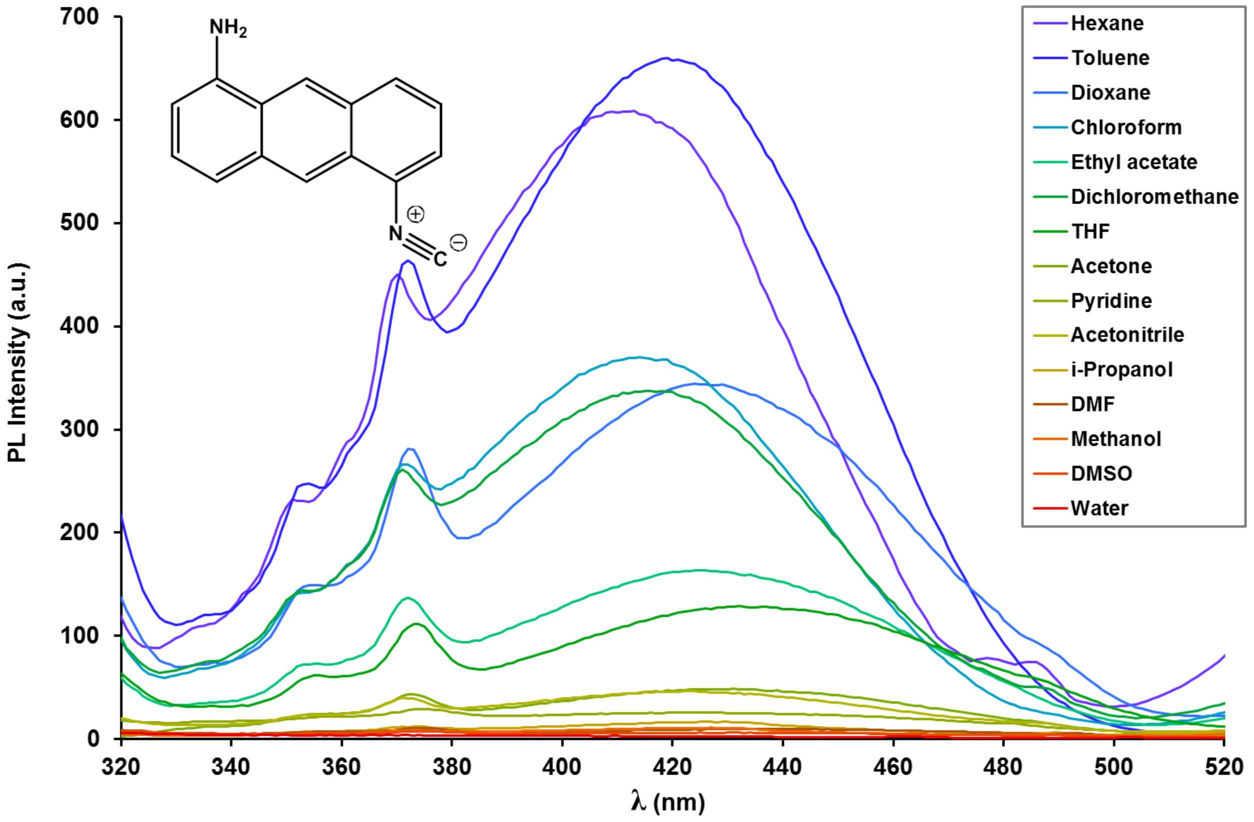

Figure S34. The excitation spectra of 1-amino-5-isocyanoanthracene (**ICAA**) recorded in solvents of different polarity. (c= 5×10^{-5} M, T=20 ºC, V=3.00 ml).

.

Chapter 3. 1,5-diisocyanoanthracene (DIA)

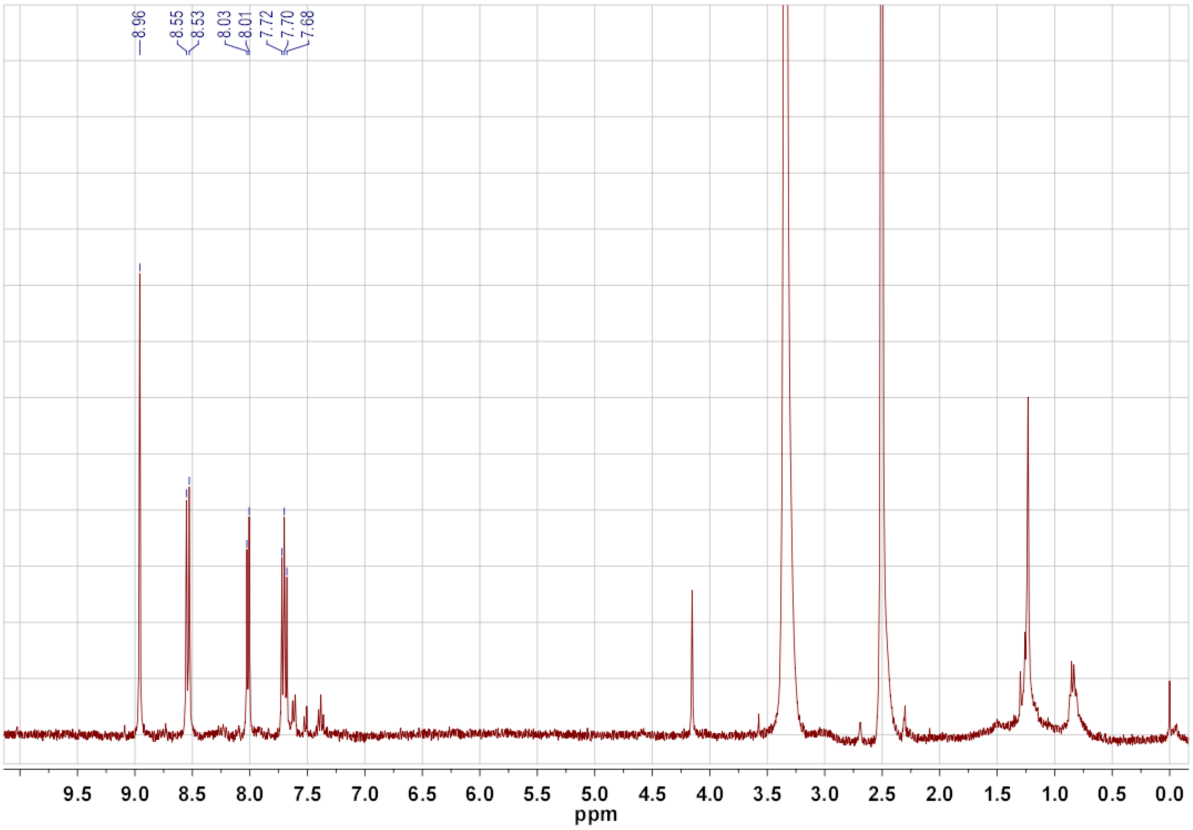

Figure S35. ^{1}H-NMR spectrum of DIA recorded at 20 °C in DMSO-d_6

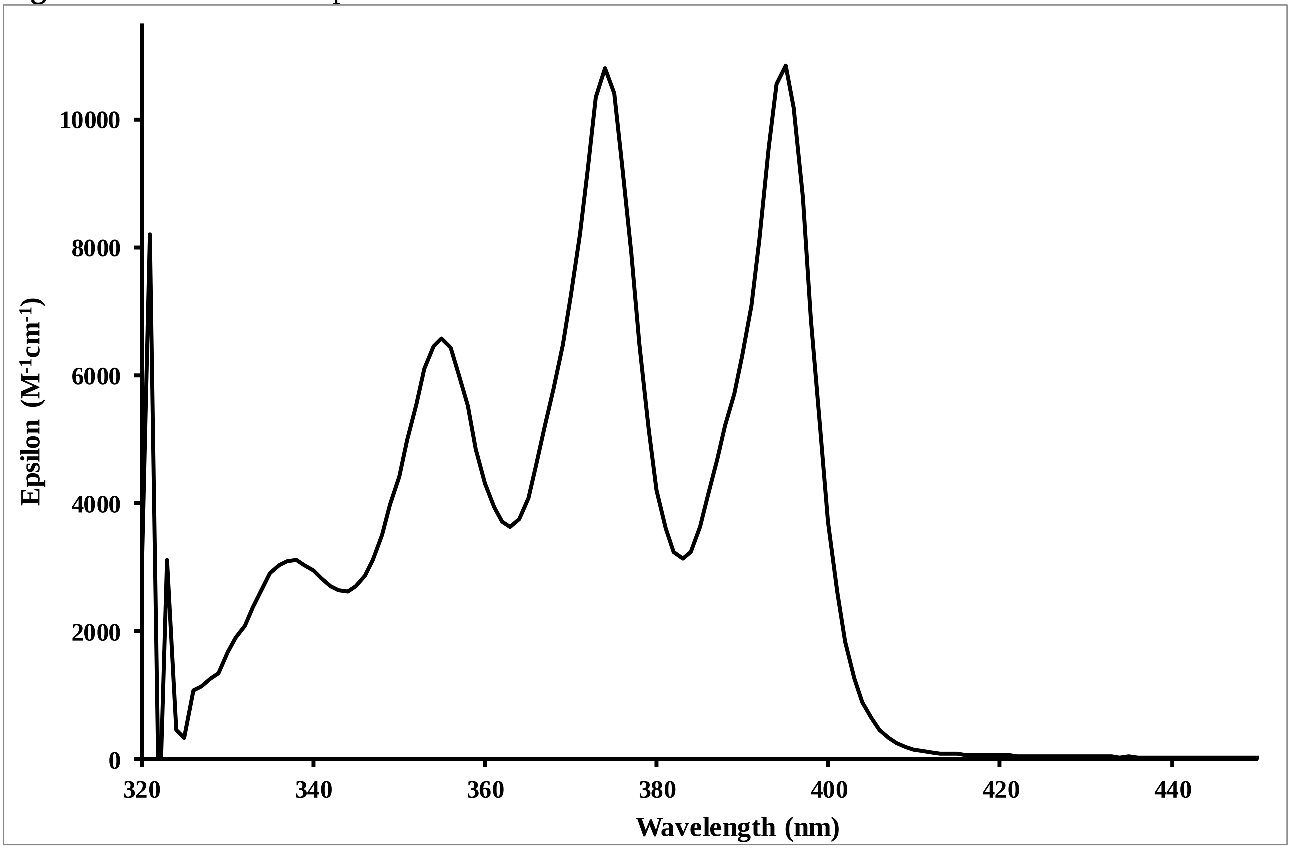

Figure S36. UV-Vis absorbance spectrum of DIA recorded in acetone

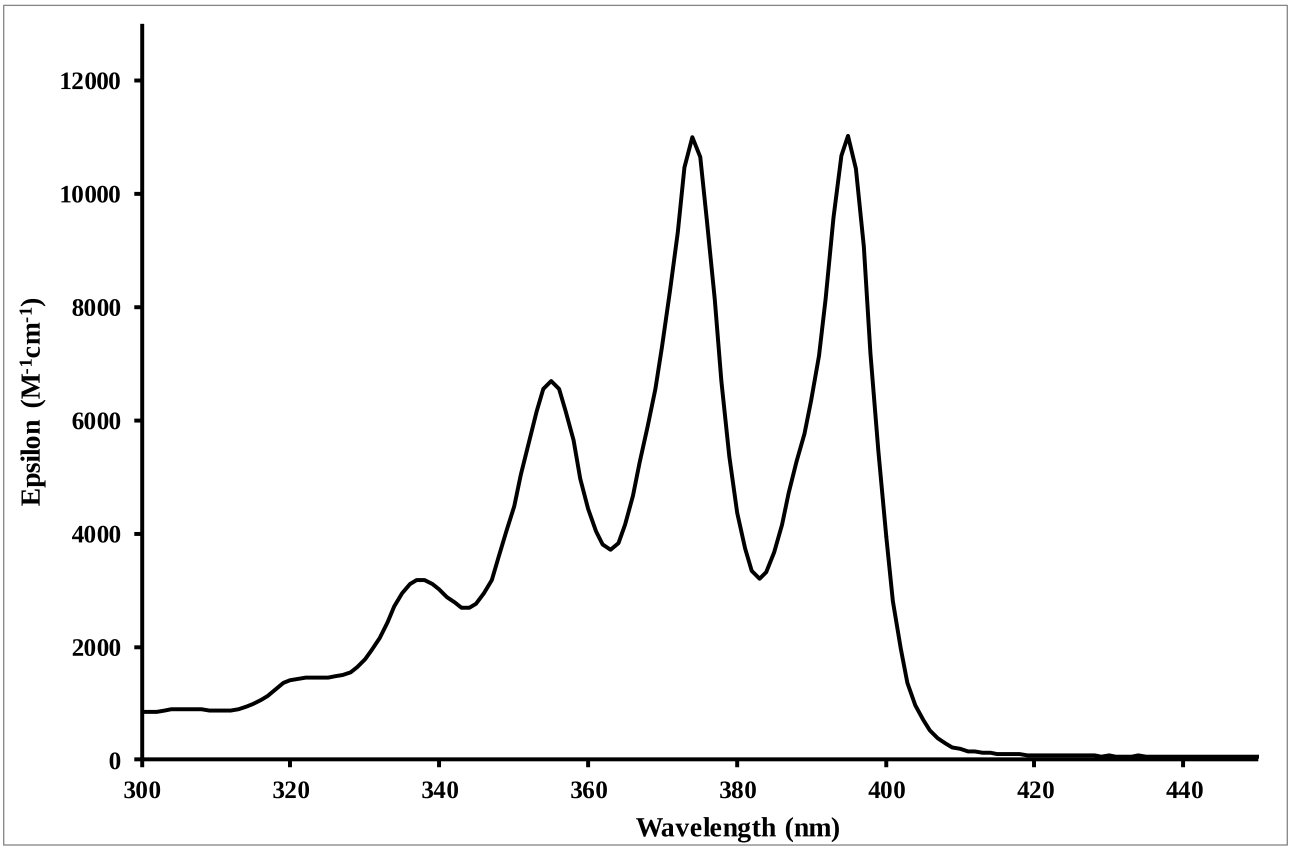

Figure S37. UV-Vis absorbance spectrum of DIA recorded in acetonitrile

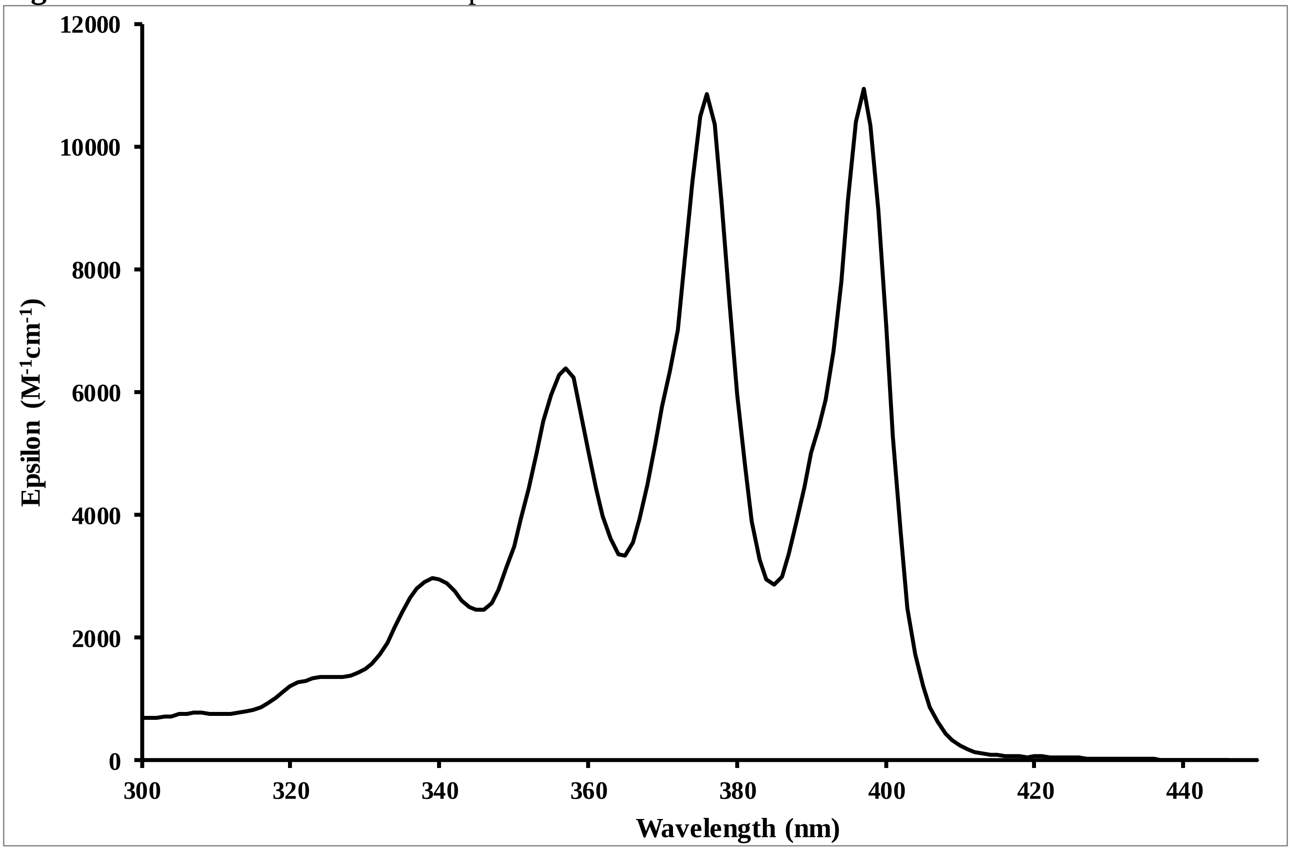

Figure S38. UV-Vis absorbance spectrum of DIA recorded in dichloromethane

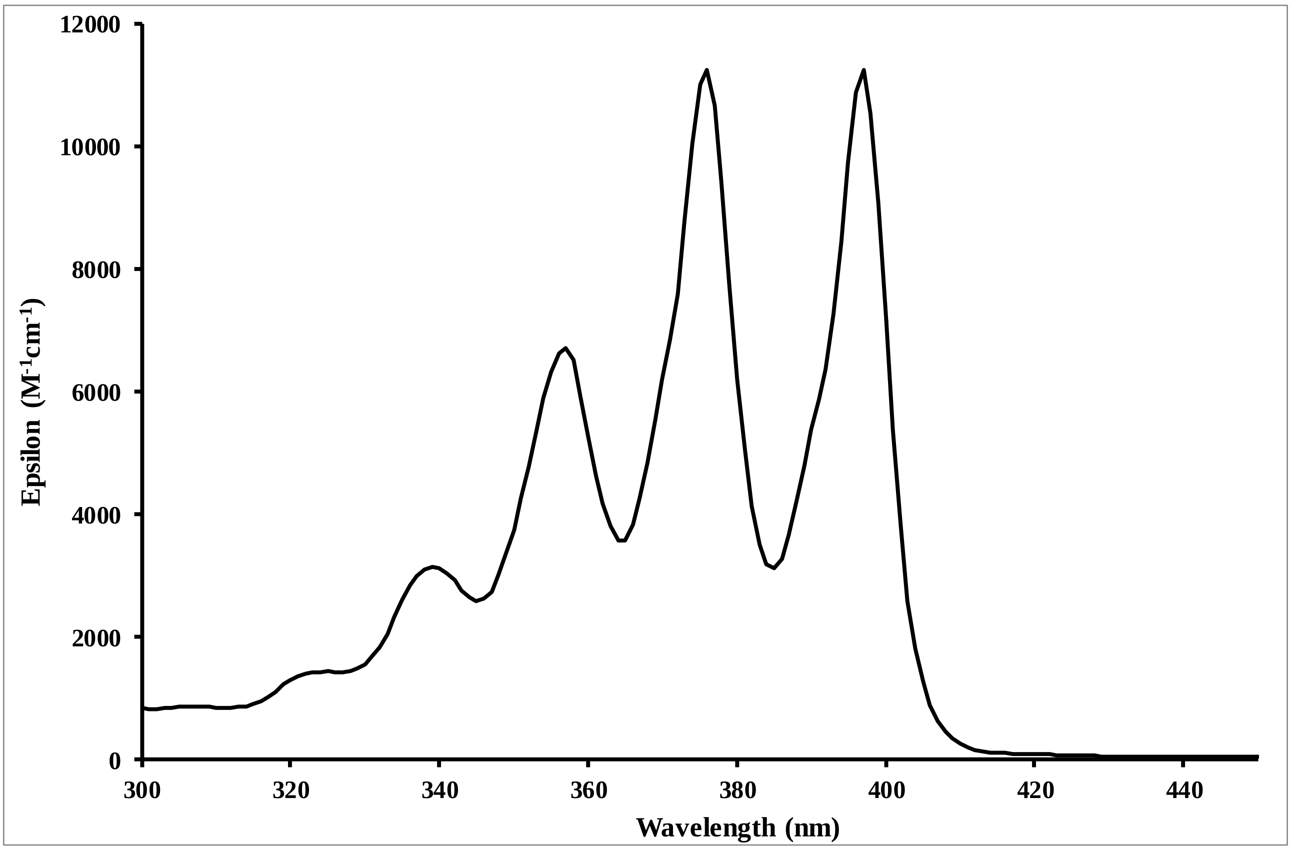

Figure S39. UV-Vis absorbance spectrum of DIA recorded in 1,4-dioxane

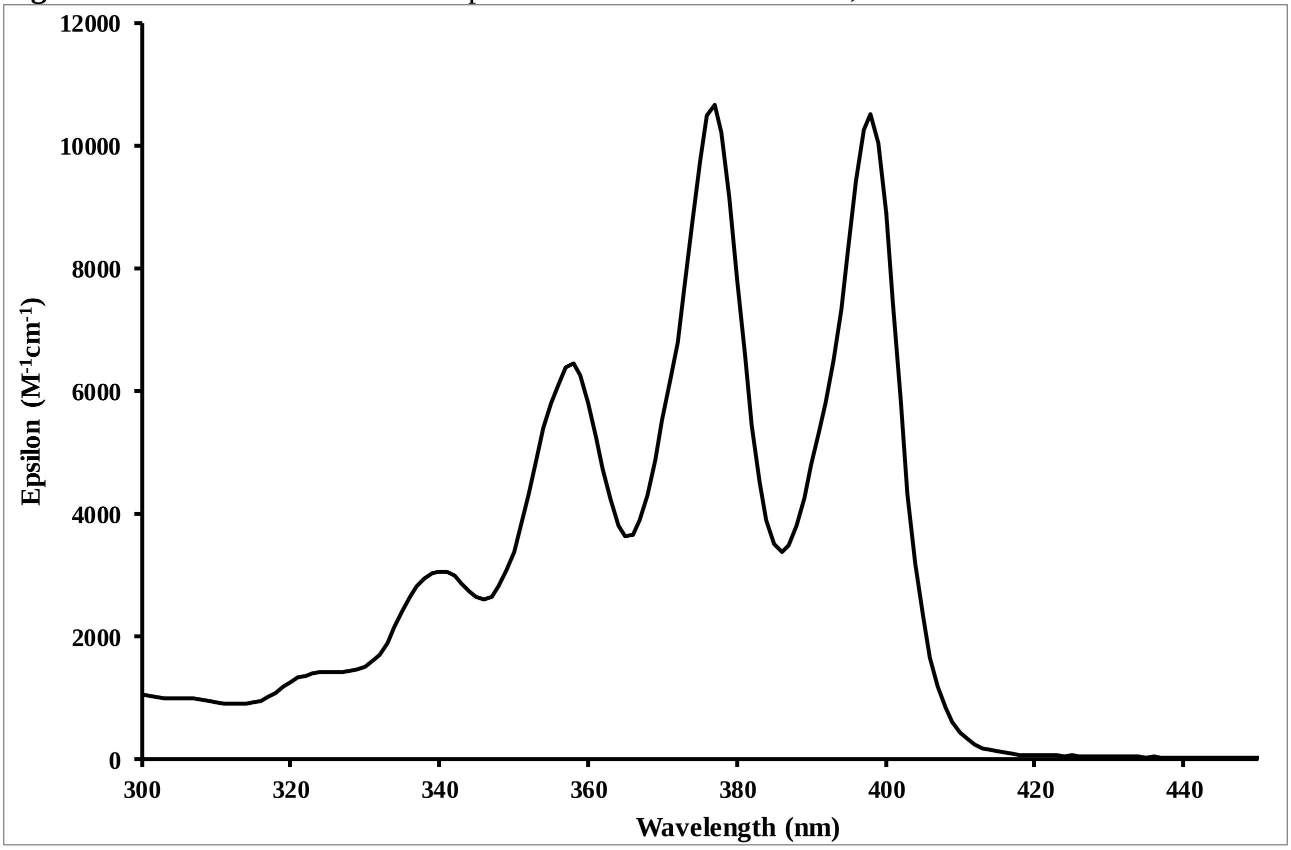

Figure S40. UV-Vis absorbance spectrum of DIA recorded in DMF

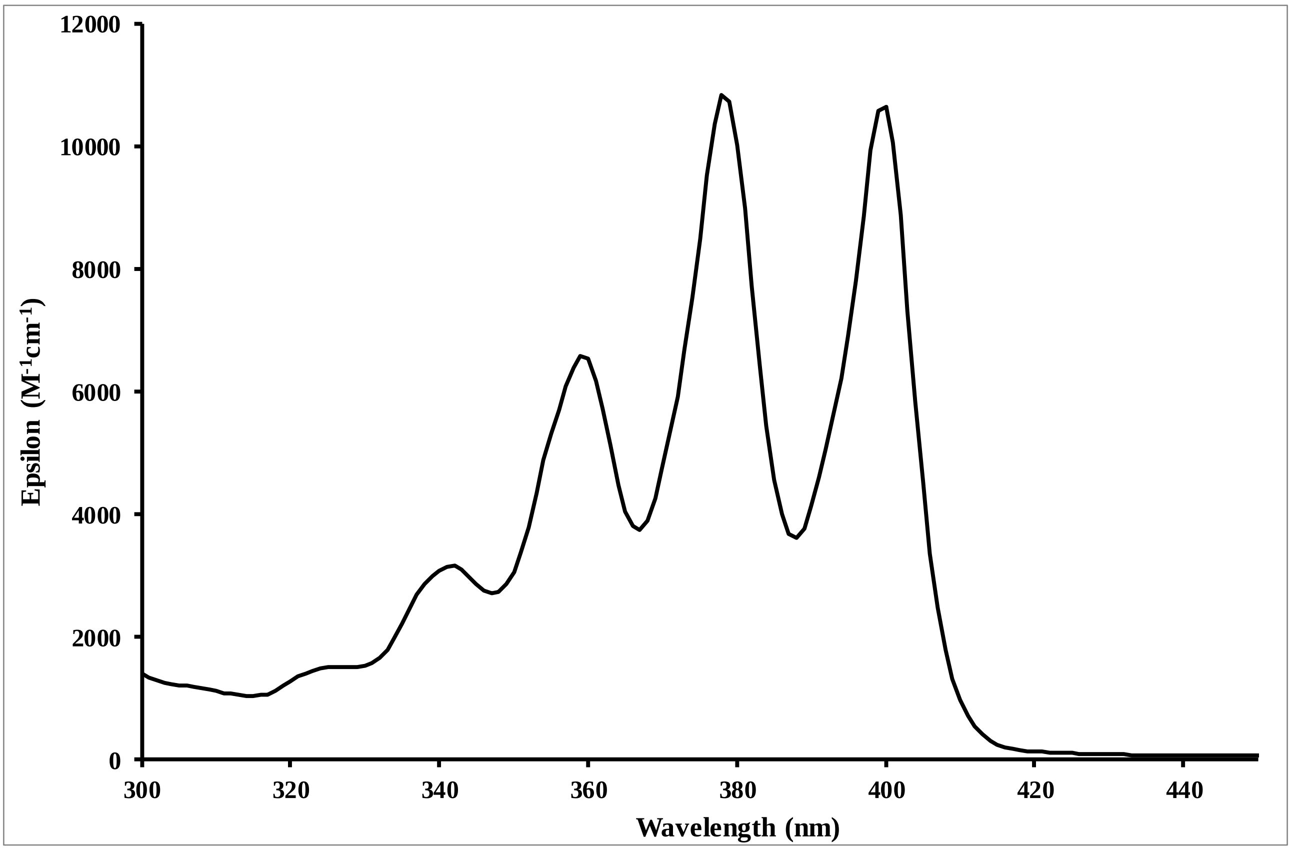

Figure S41. UV-Vis absorbance spectrum of DIA recorded in DMSO

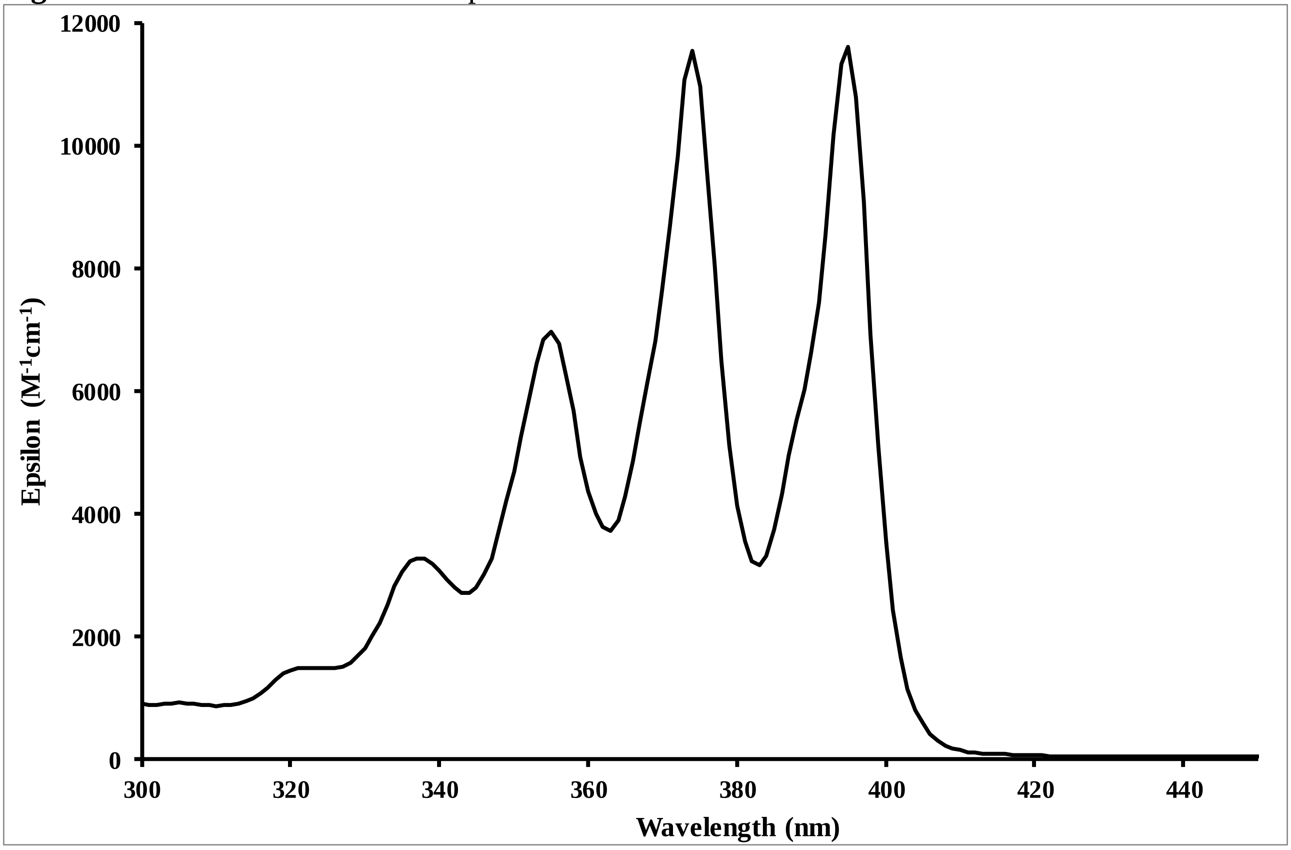

Figure S42. UV-Vis absorbance spectrum of DIA recorded in ethyl acetate

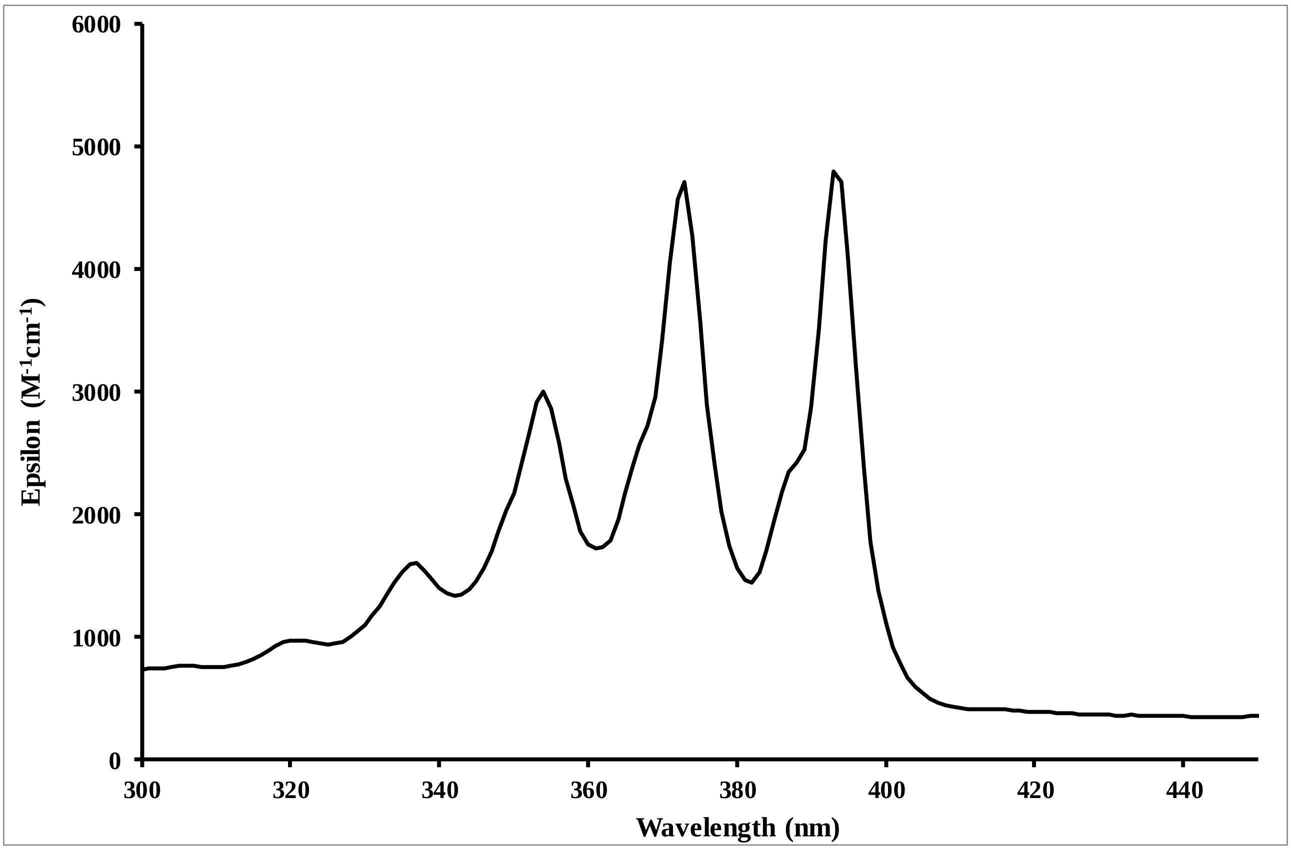

Figure S43. UV-Vis absorbance spectrum of DIA recorded in hexane

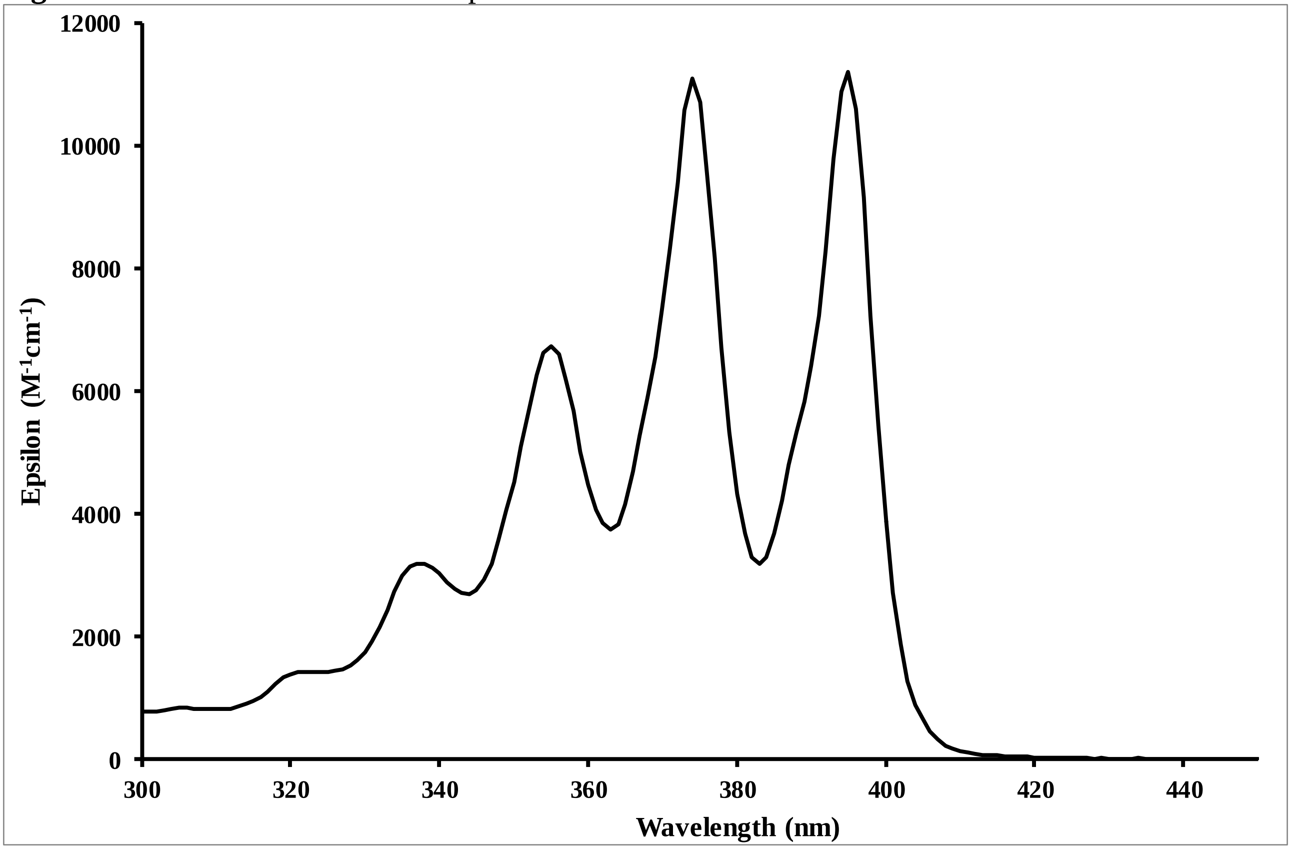

Figure S44. UV-Vis absorbance spectrum of DIA recorded in 2-propanol

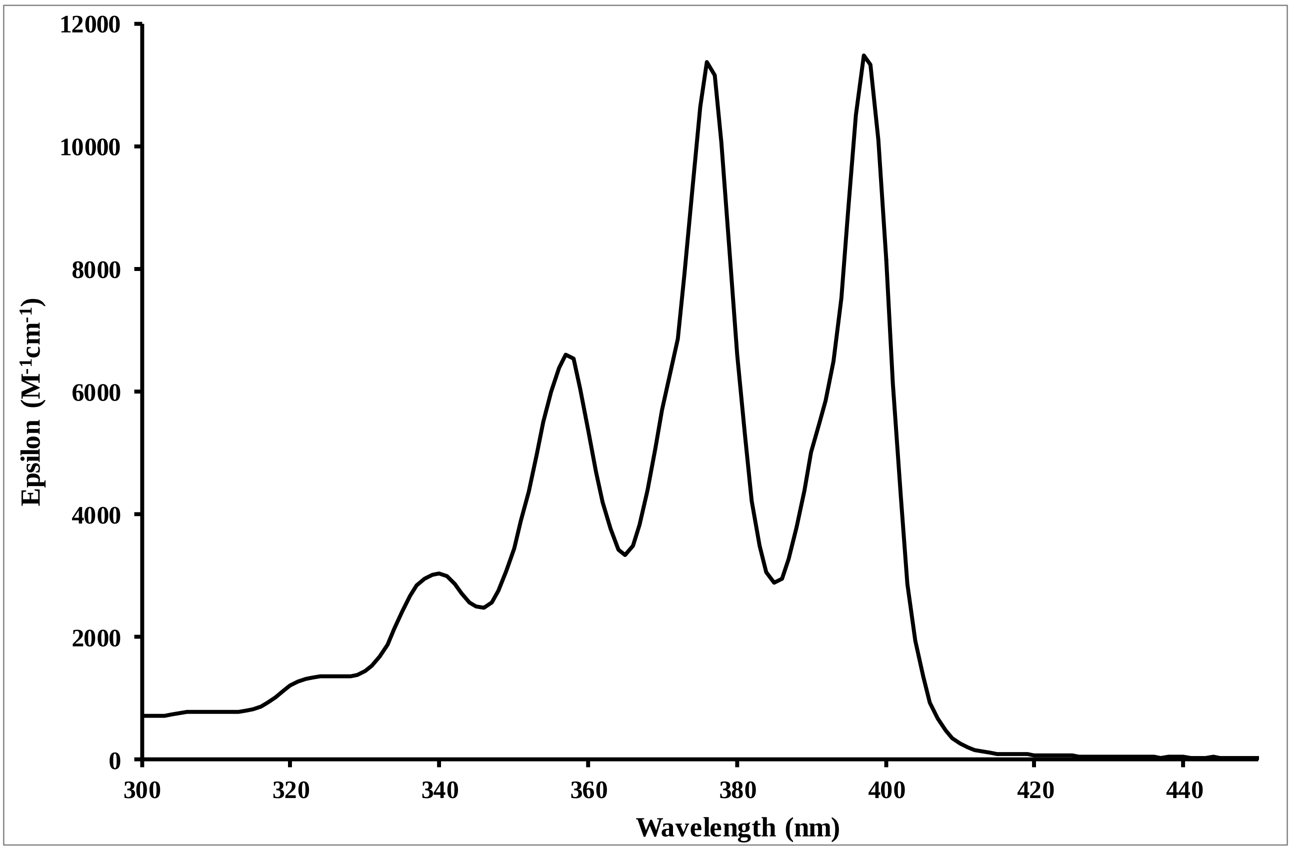

Figure S45. UV-Vis absorbance spectrum of DIA recorded in chloroform

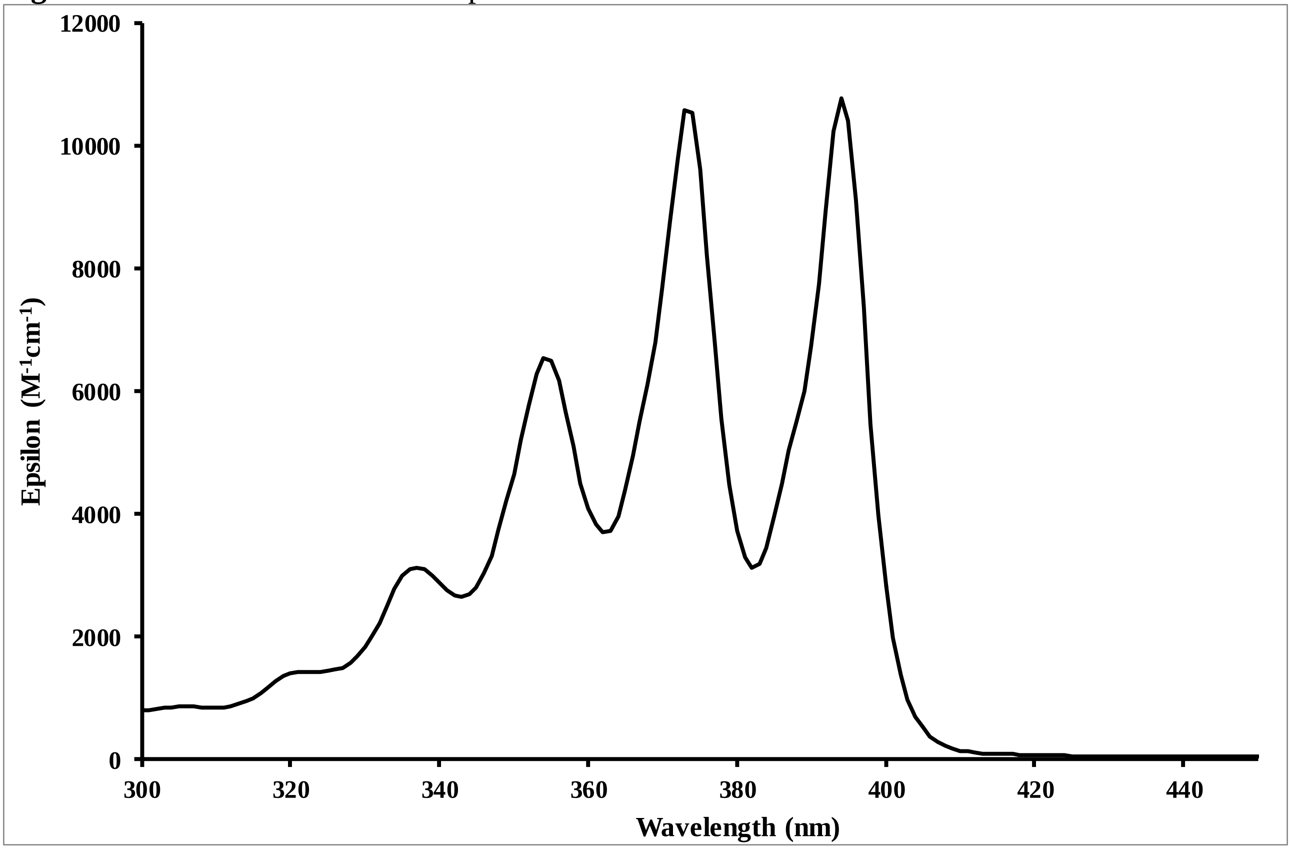

Figure S46. UV-Vis absorbance spectrum of DIA recorded in methanol

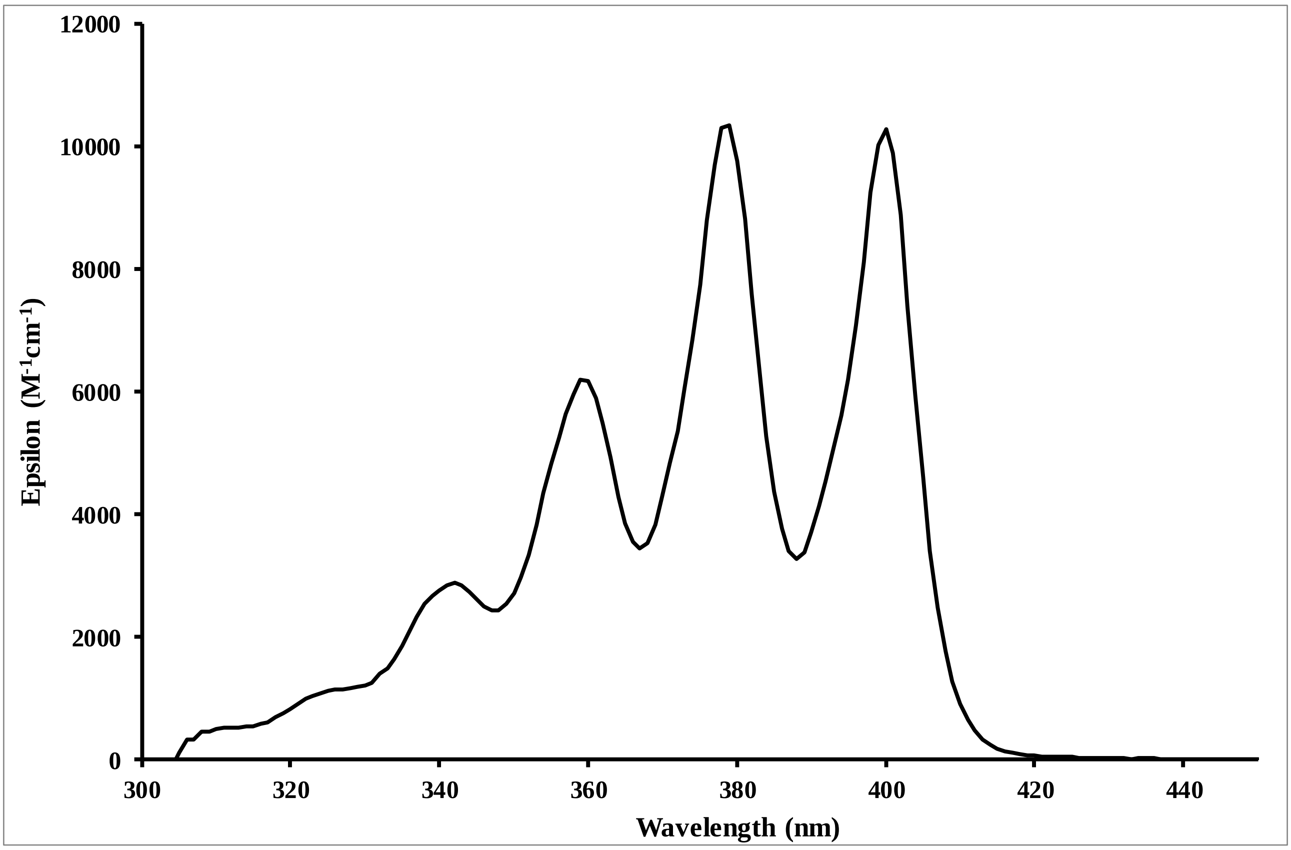

Figure S47. UV-Vis absorbance spectrum of DIA recorded in pyridine

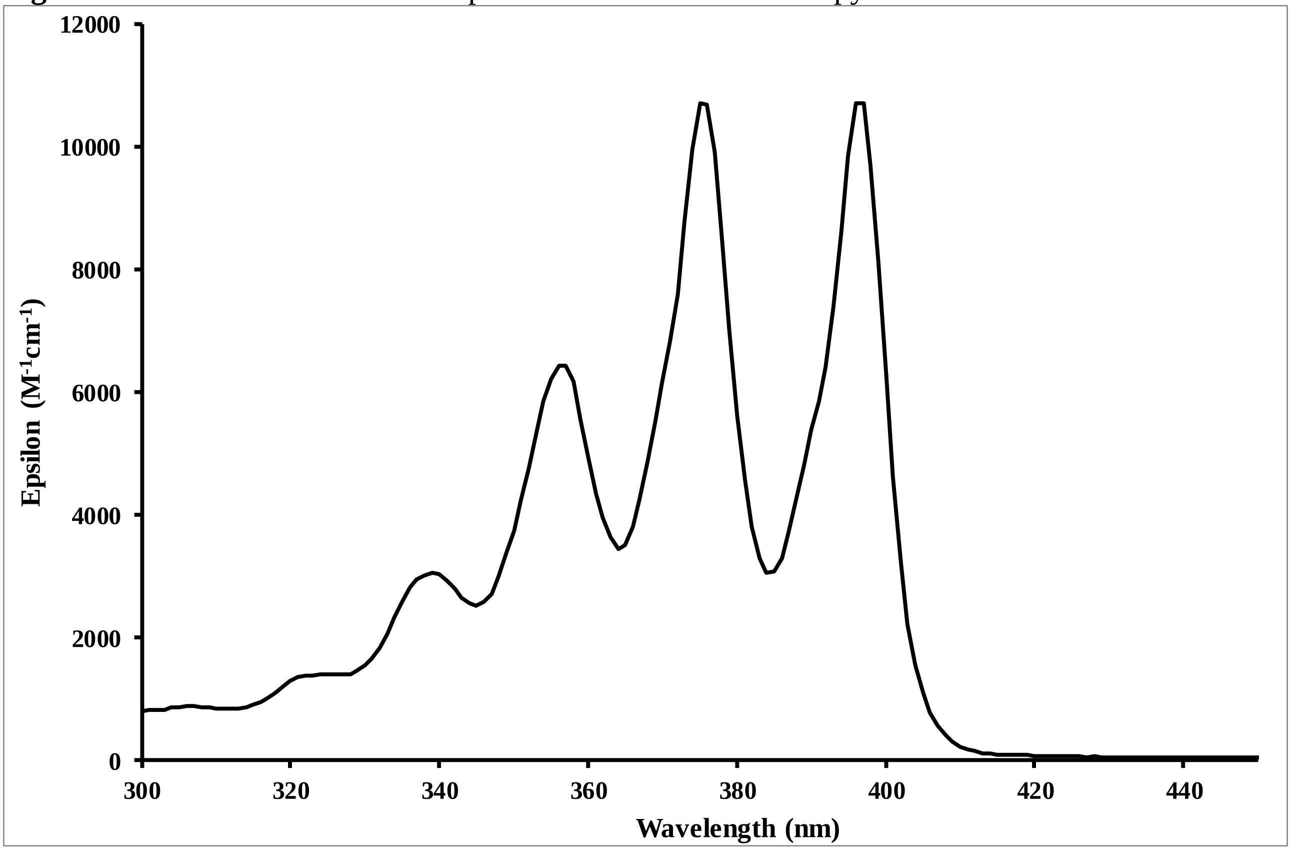

Figure S48. UV-Vis absorbance spectrum of DIA recorded in THF

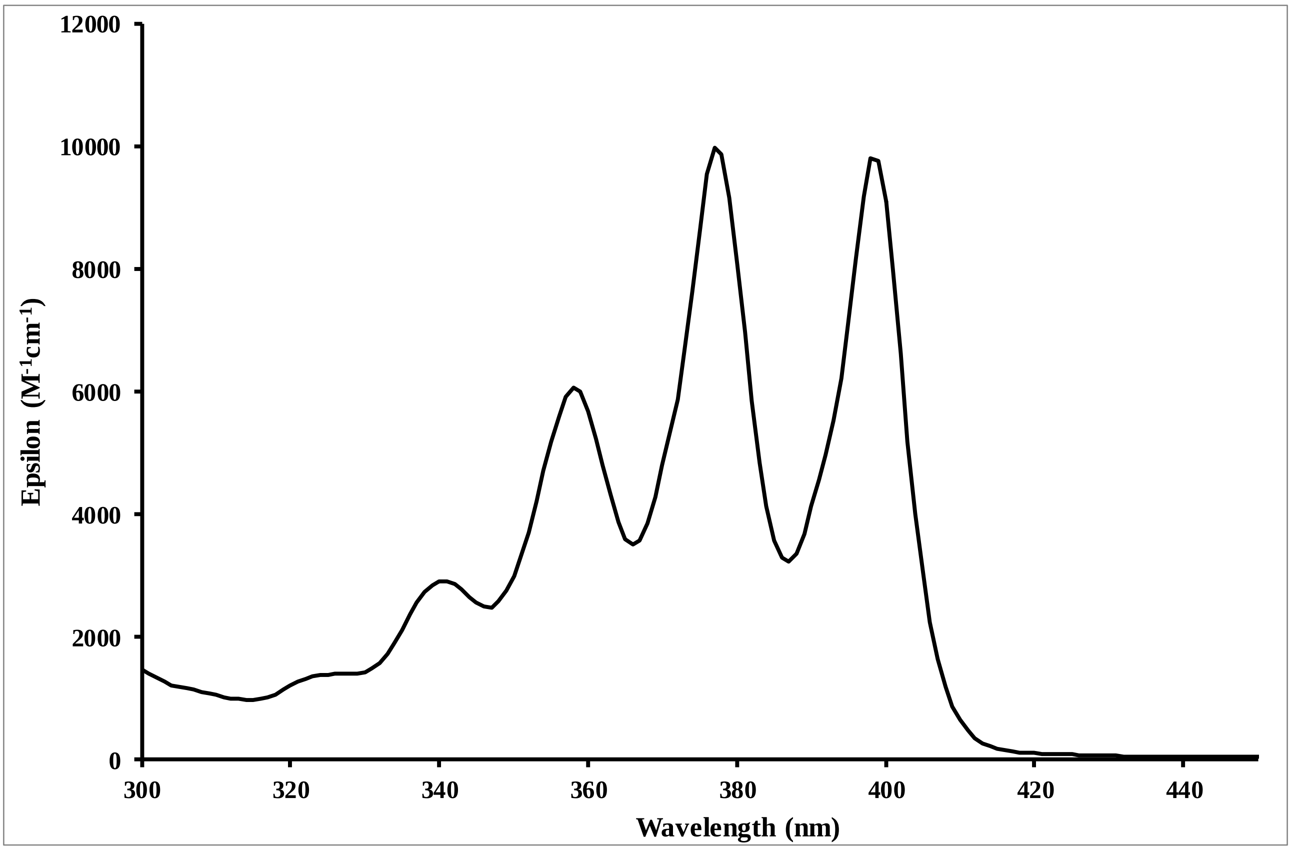

Figure S49. UV-Vis absorbance spectrum of DIA recorded in toluene

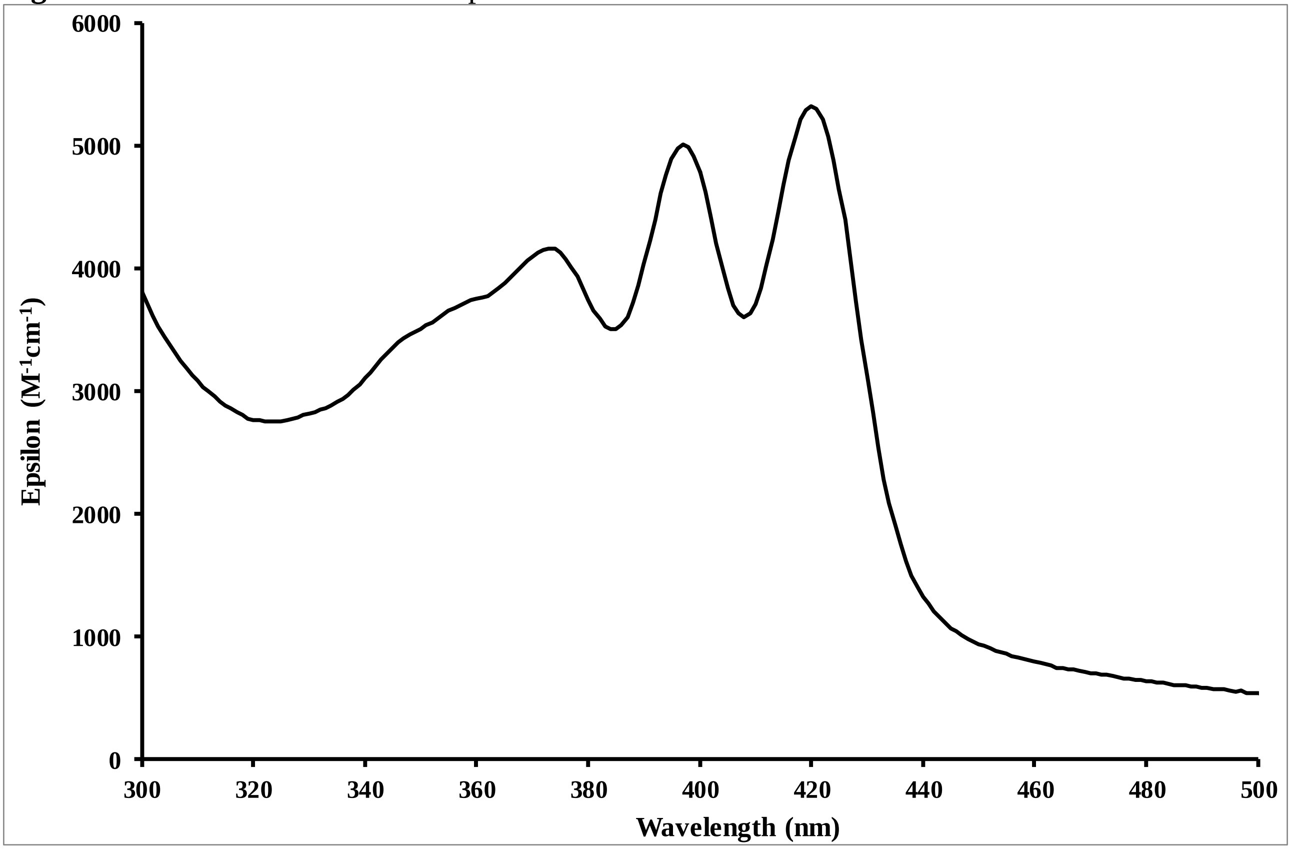

Figure S50. UV-Vis absorbance spectrum of DIA recorded in water

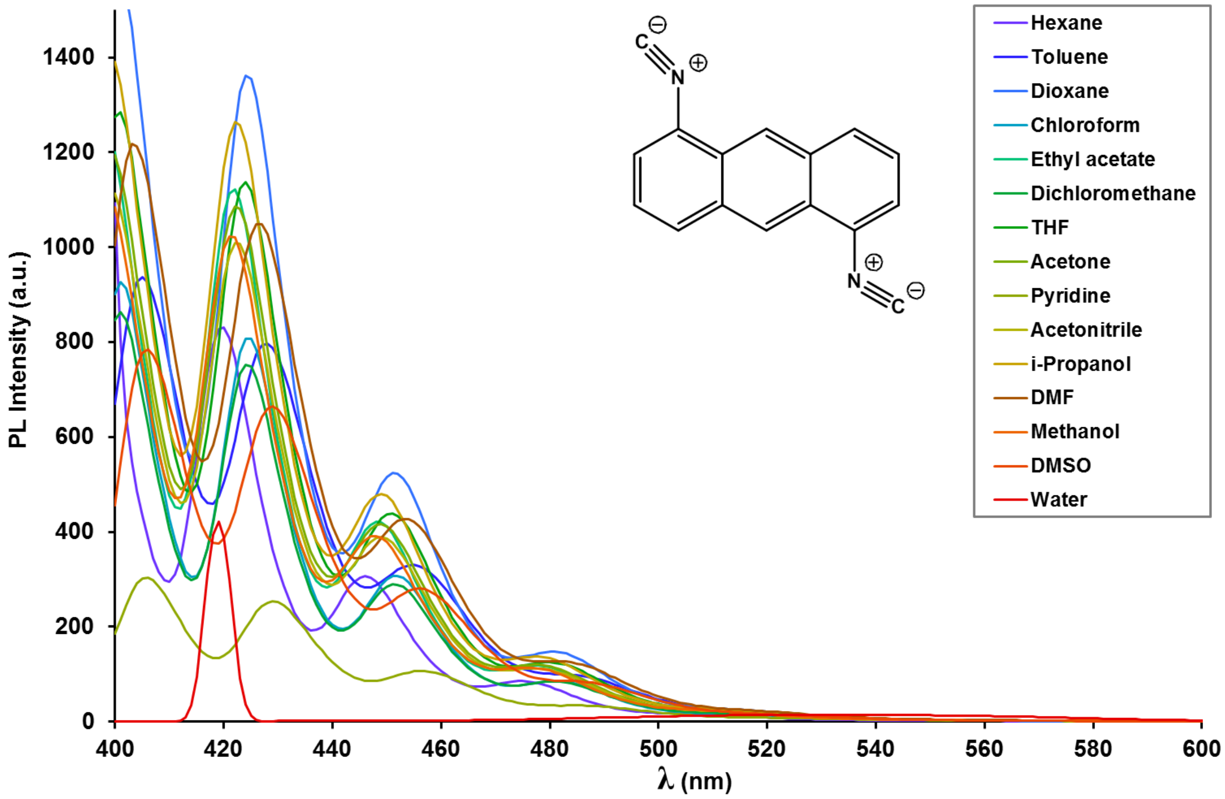

Figure S51. The emission spectra of 1,5-diisocyanoanthracene (**DIA**) recorded in solvents of different polarity. (c= 5×10^{-5} M, T=20 ºC, V=3.00 ml).

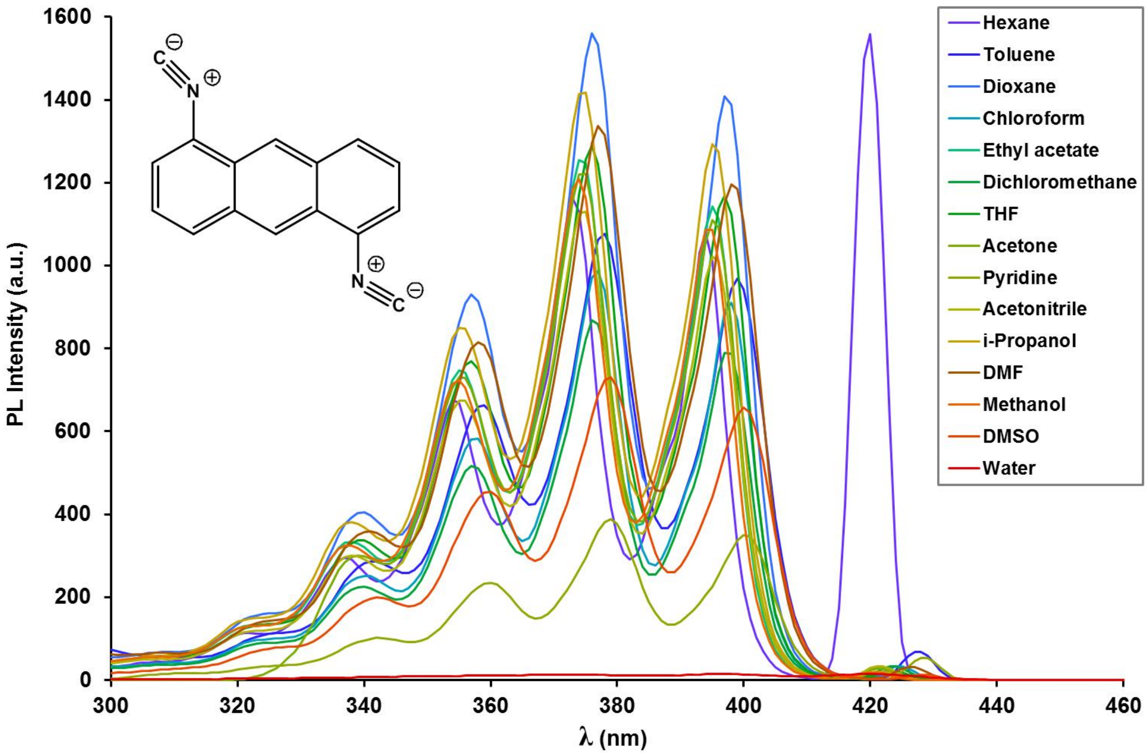

Figure S52. The excitation spectra of 1,5-diisocyanoanthracene (**DIA**) recorded in solvents of different polarity. (c= 5×10^{-5} M, T=20 ºC, V=3.00 ml).

Solvent	Polarity Index	$\lambda_{em,max,1}$ (nm)	$\lambda_{em,max,2}$ (nm)	$\lambda_{em,max,3}$ (nm)	$\lambda_{ex,max,1}$ (nm)	$\lambda_{ex,max,2}$ (nm)	$\lambda_{ex,max,3}$ (nm)	$\lambda_{ex,max,4}$ (nm)	Stokes shift (cm^{-1})	ε (M^{-1})	φ_F (%)
Hexane	0.0	397	420	446	337	354	373	394	3000	-	-
Toluene	2.4	405	428	455	341	359	378	399	3091	9989	38
Dichloromethane	3.1	401	424	451	340	357	376	397	3011	10942	25
i-propanol	3.9	398	422	449	338	355	375	395	2970	11217	38
THF	4.0	401	424	451	339	357	376	397	3011	10720	38
Chloroform	4.1	401	425	452	340	358	377	398	2996	11488	26
Ethyl acetate	4.4	397	422	448	338	355	374	395	3041	11618	30
Dioxane	4.8	401	424	451	340	357	376	397	3011	11258	44
Acetone	5.1	397	422	449	339	355	375	395	2970	10852	33
Methanol	5.1	397	421	448	337	355	374	395	2985	10784	30
Pyridine	5.3	406	429	456	342	360	379	400	3075	10360	12
Acetonitrile	5.8	397	422	449	338	356	375	395	2970	11034	31
Dimethyl formamide	6.4	403	427	453	341	358	377	398	3106	10667	42
Dimethyl sulfoxide	7.2	406	429	456	342	360	379	400	3075	10836	30
Water	9.0	-	-	-	-	-	-	-	-	-	-

Chapter IV. 1-N-methylamino-5-isocyanoanthracene (MICAA)

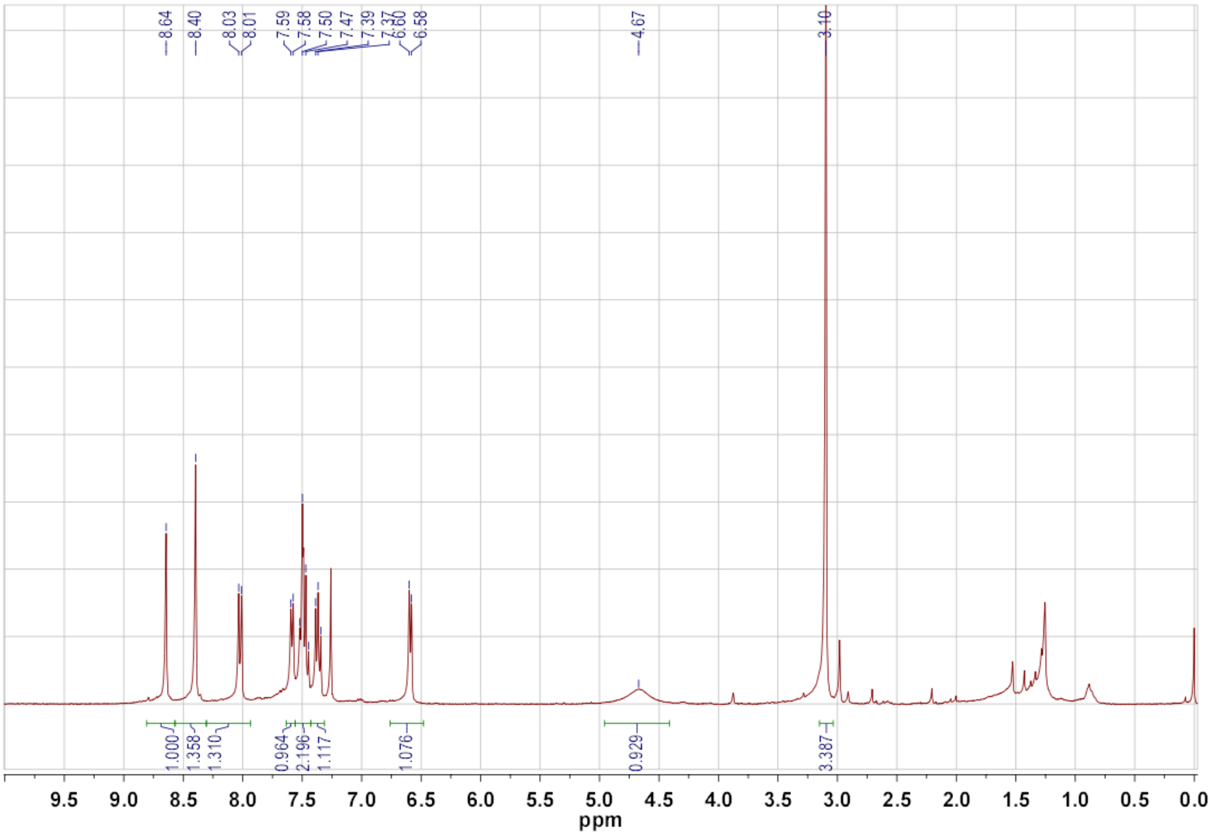

Figure S53. ^{1}H-NMR spectra of MICAA recorded at 20 °C in $CDCl_3$

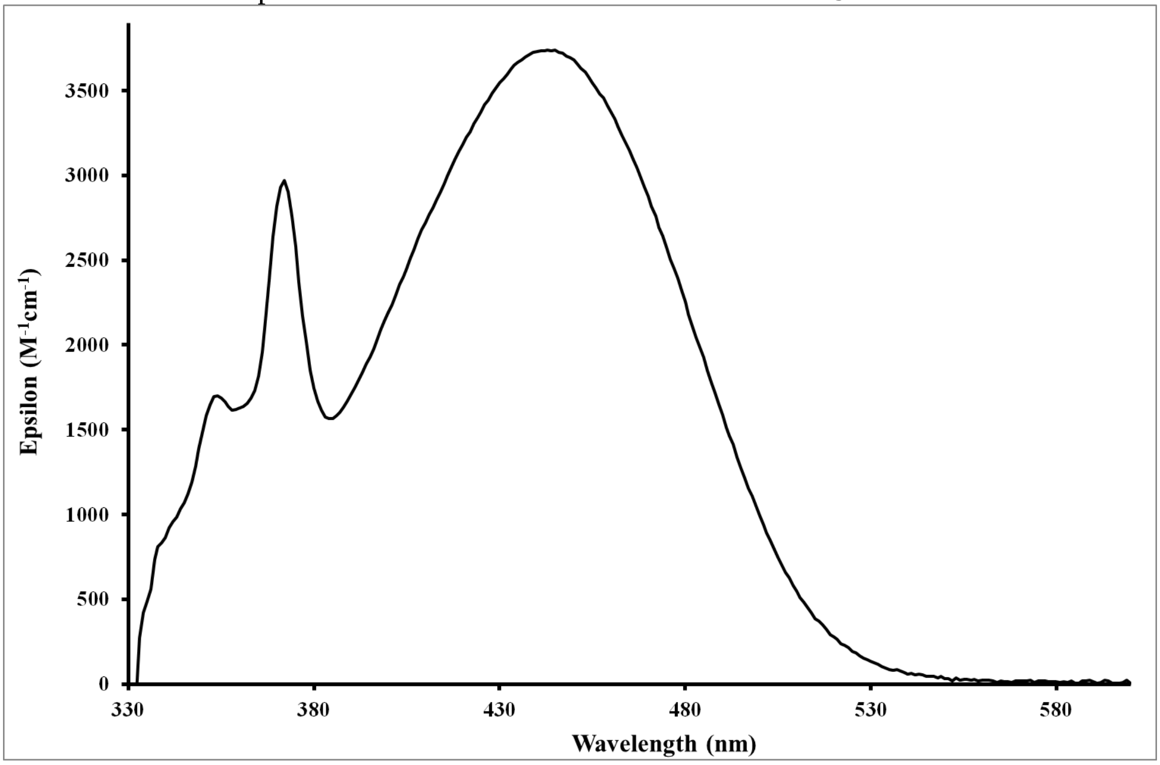

Figure S54. UV-Vis absorbance spectrum of MICAA recorded in acetone

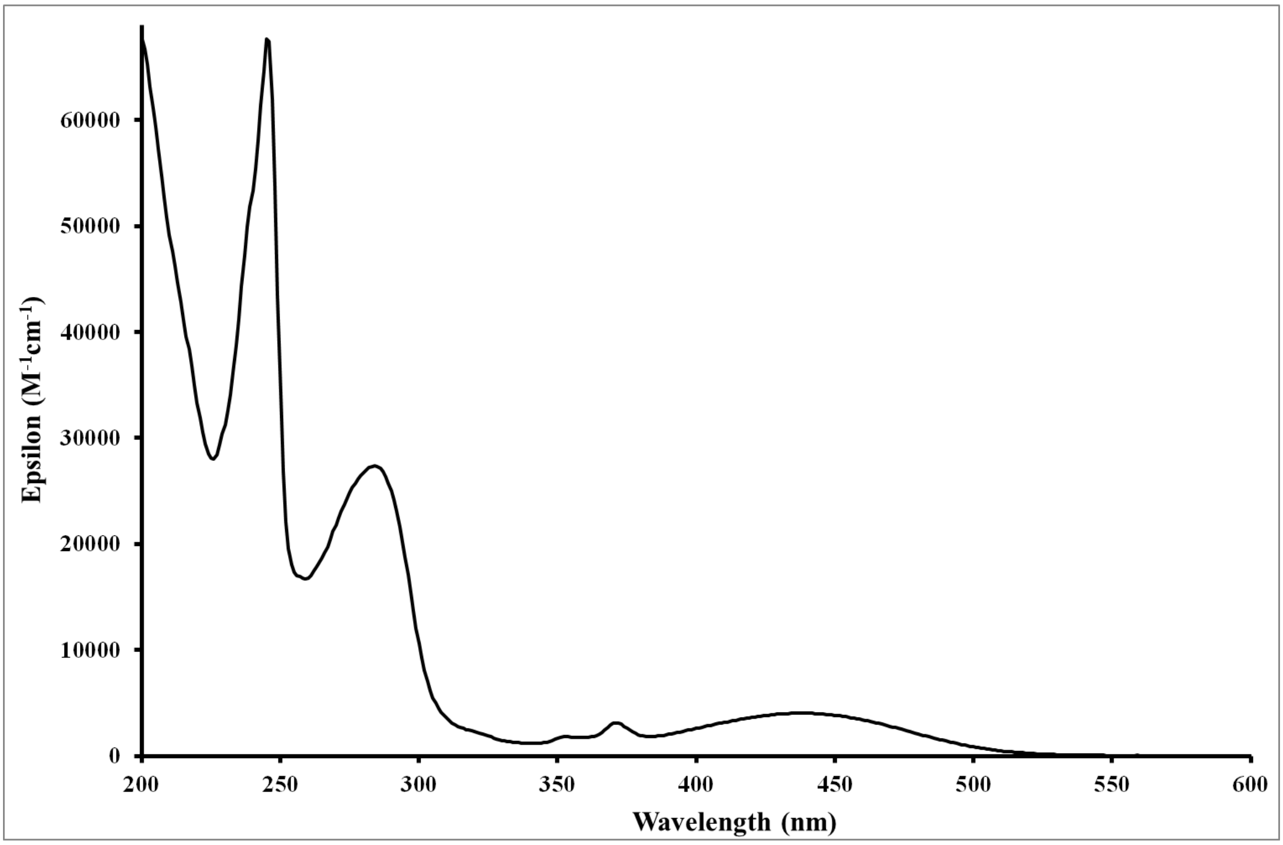

Figure S55. UV-Vis absorbance spectrum of MICAA recorded in acetonitrile

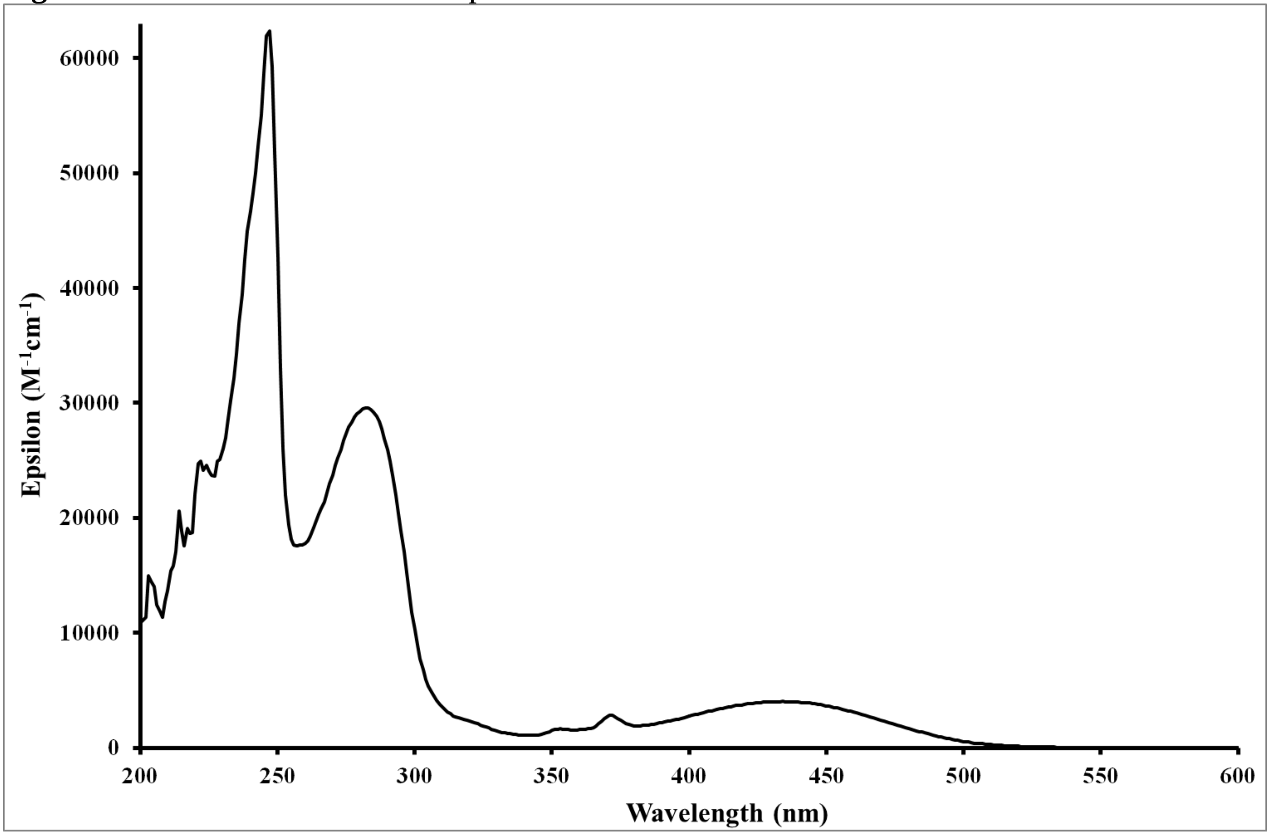

Figure S56. UV-Vis absorbance spectrum of MICAA recorded in dichloromethane

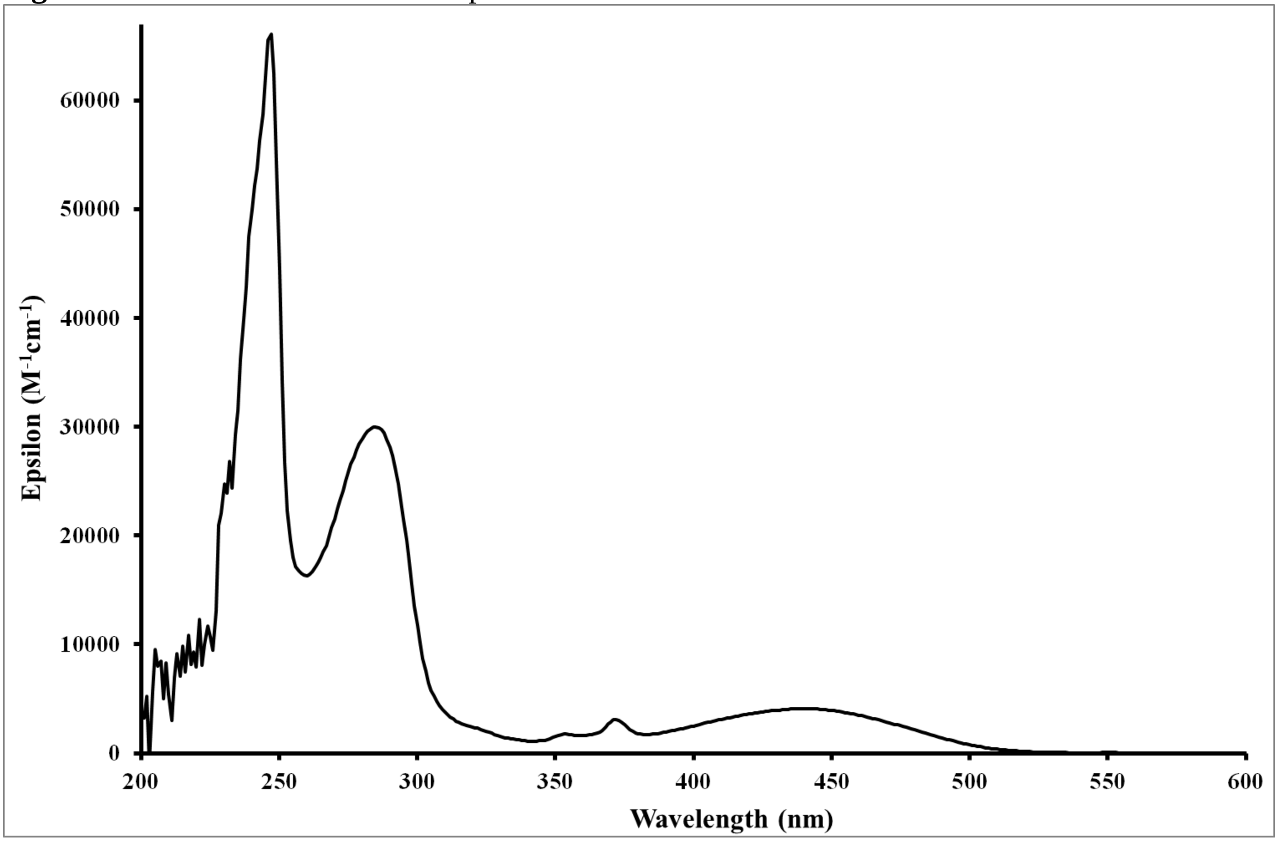

Figure S57. Dioxane UV-Vis absorbance spectrum of MICAA recorded in dioxane

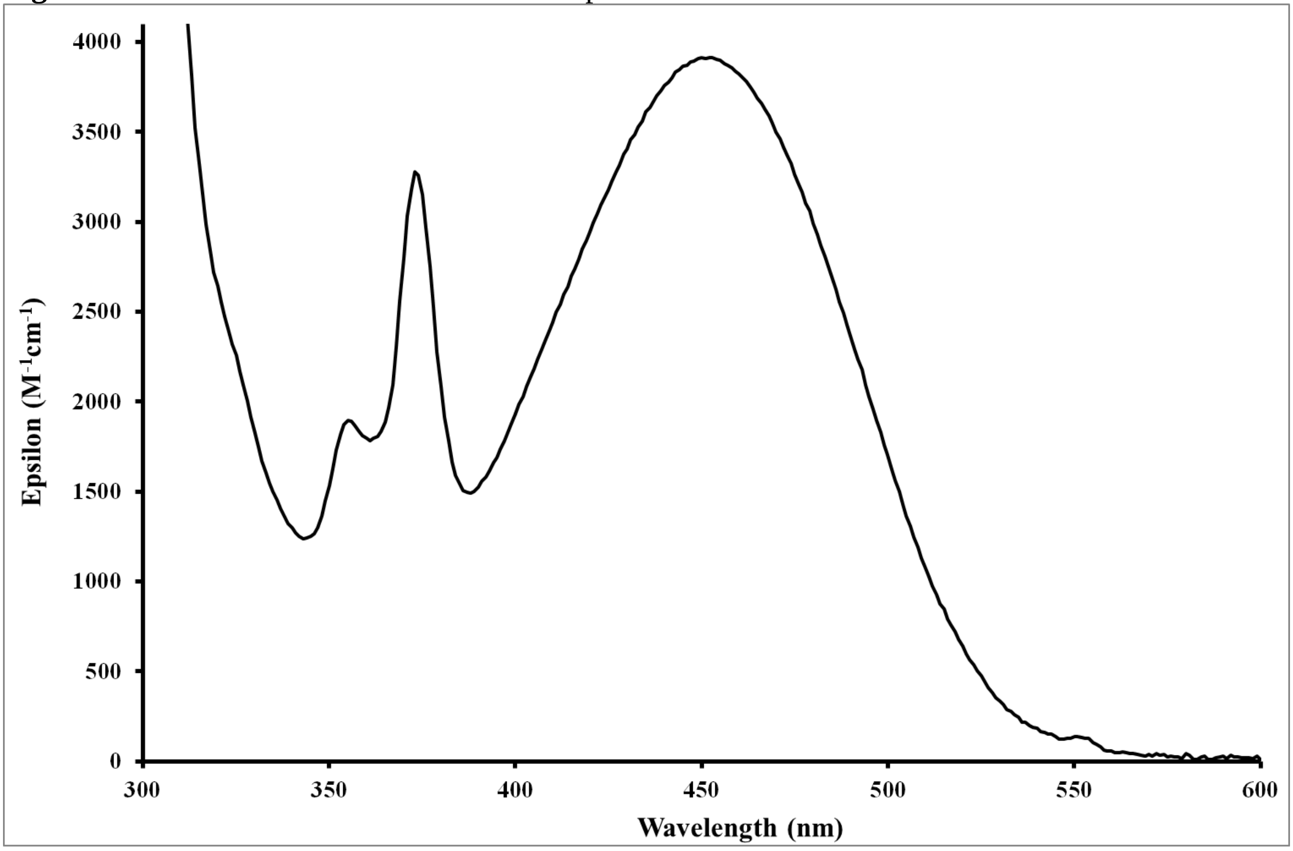

Figure S58. UV-Vis absorbance spectrum of MICAA recorded in DMF

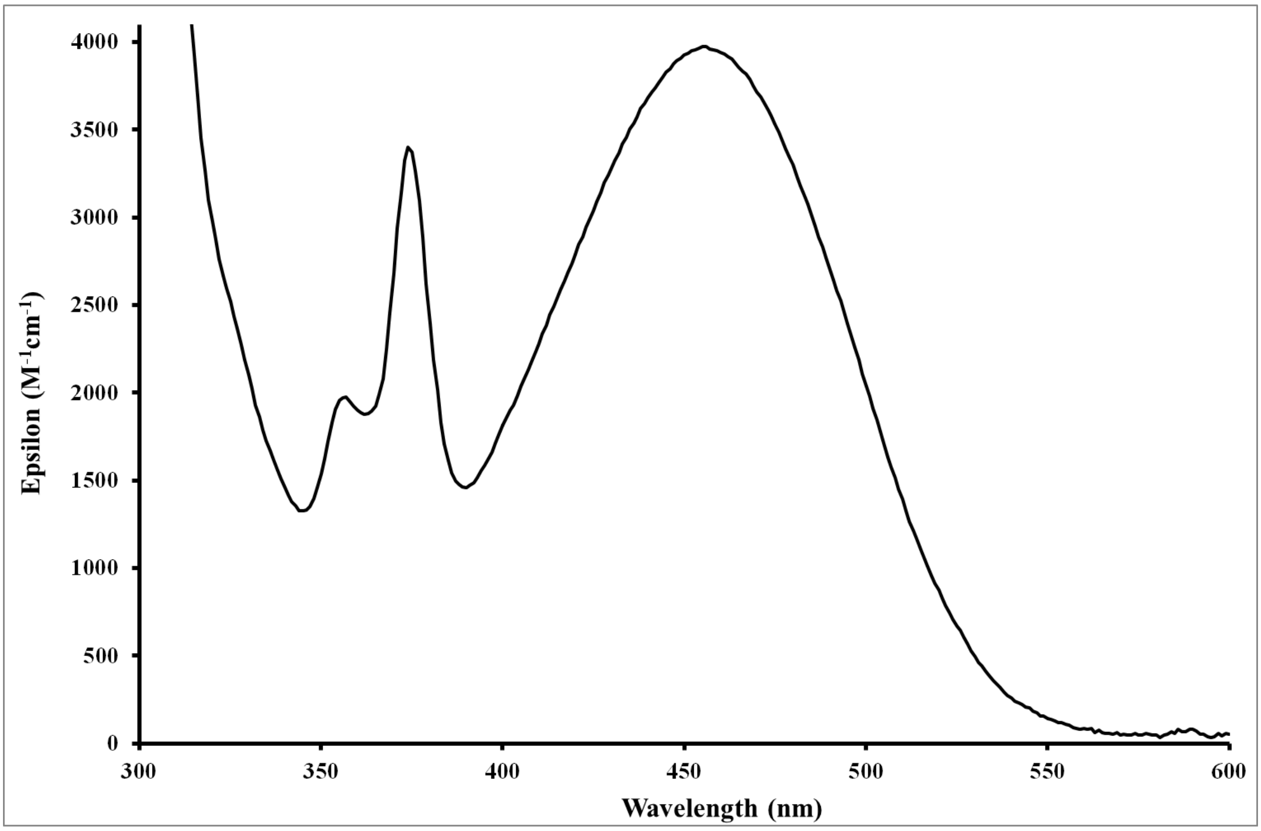

Figure S59. UV-Vis absorbance spectrum of MICAA recorded in DMSO

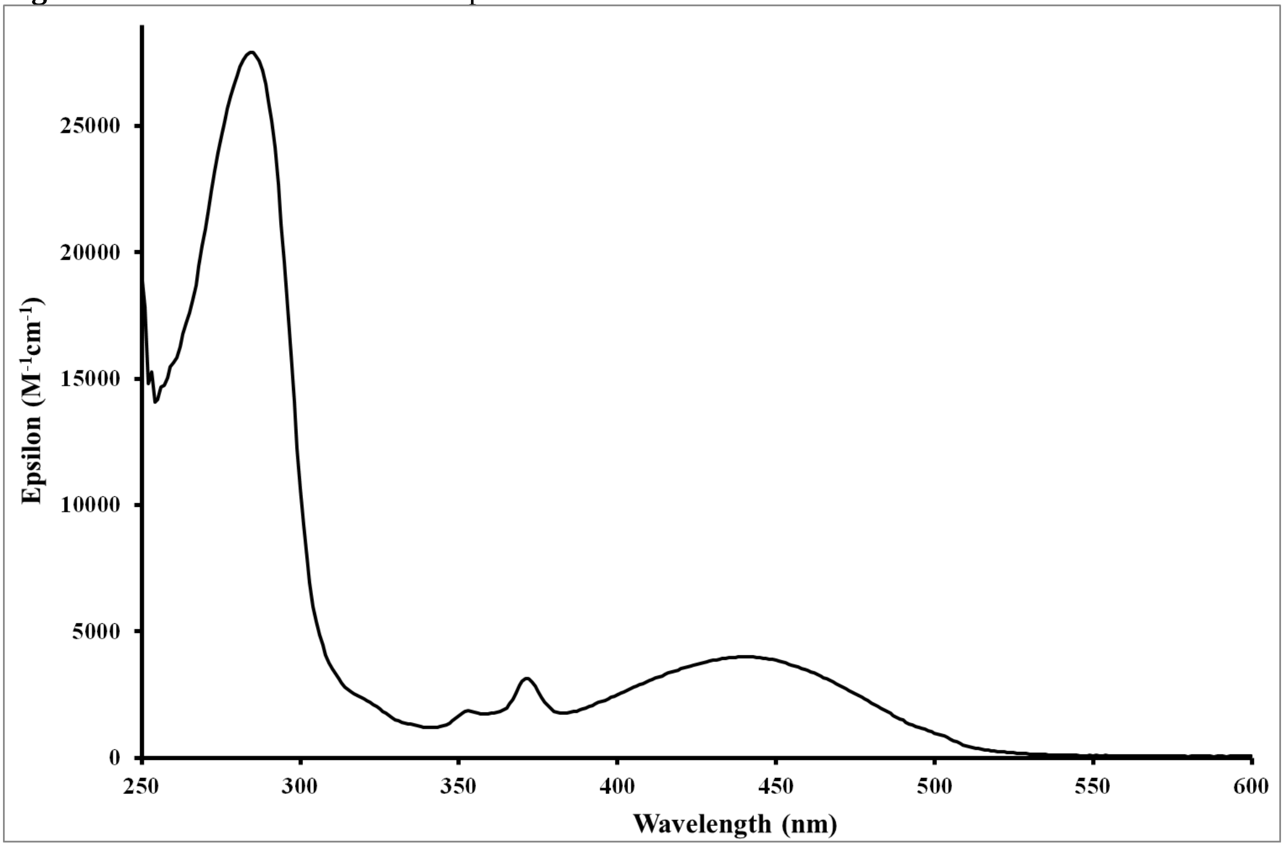

Figure S60. UV-Vis absorbance spectrum of MICAA recorded in ethyl-acetate

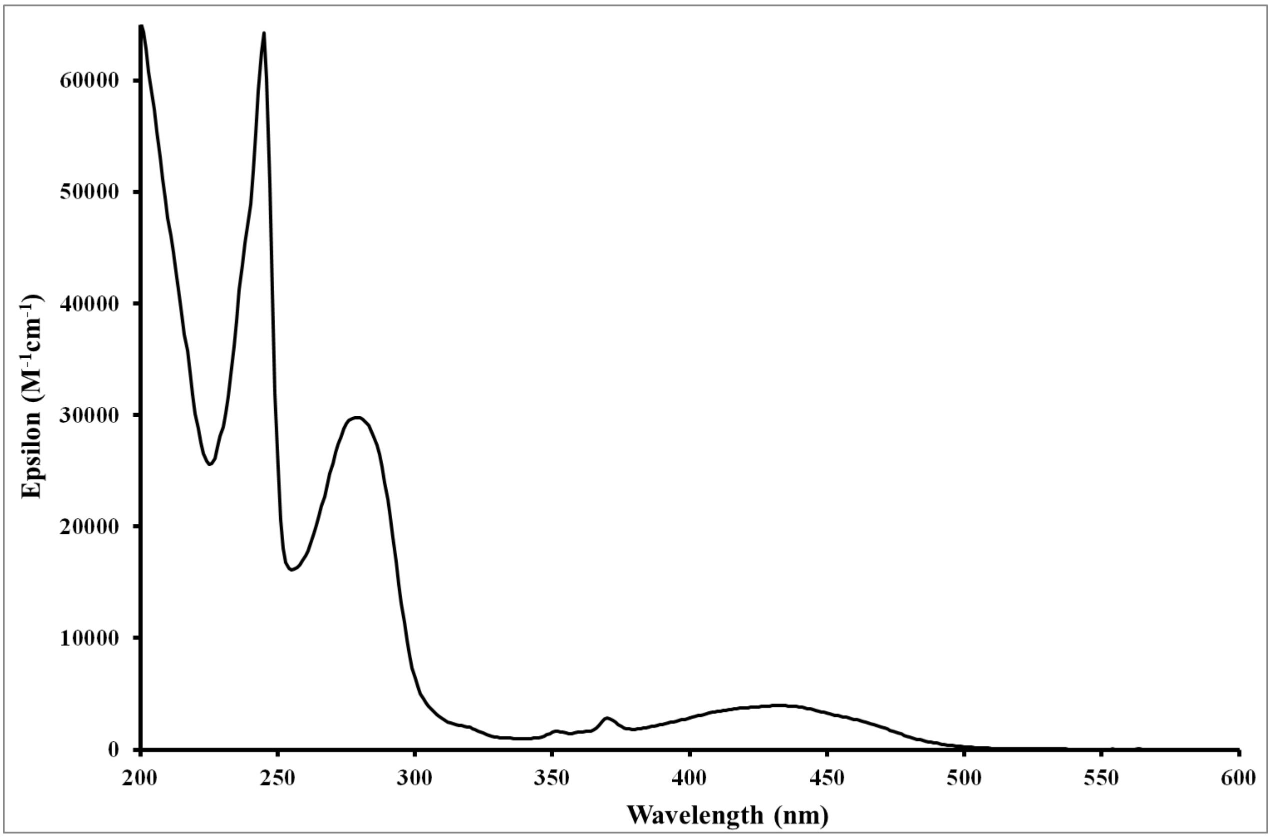

Figure S61. UV-Vis absorbance spectrum of MICAA recorded in hexane

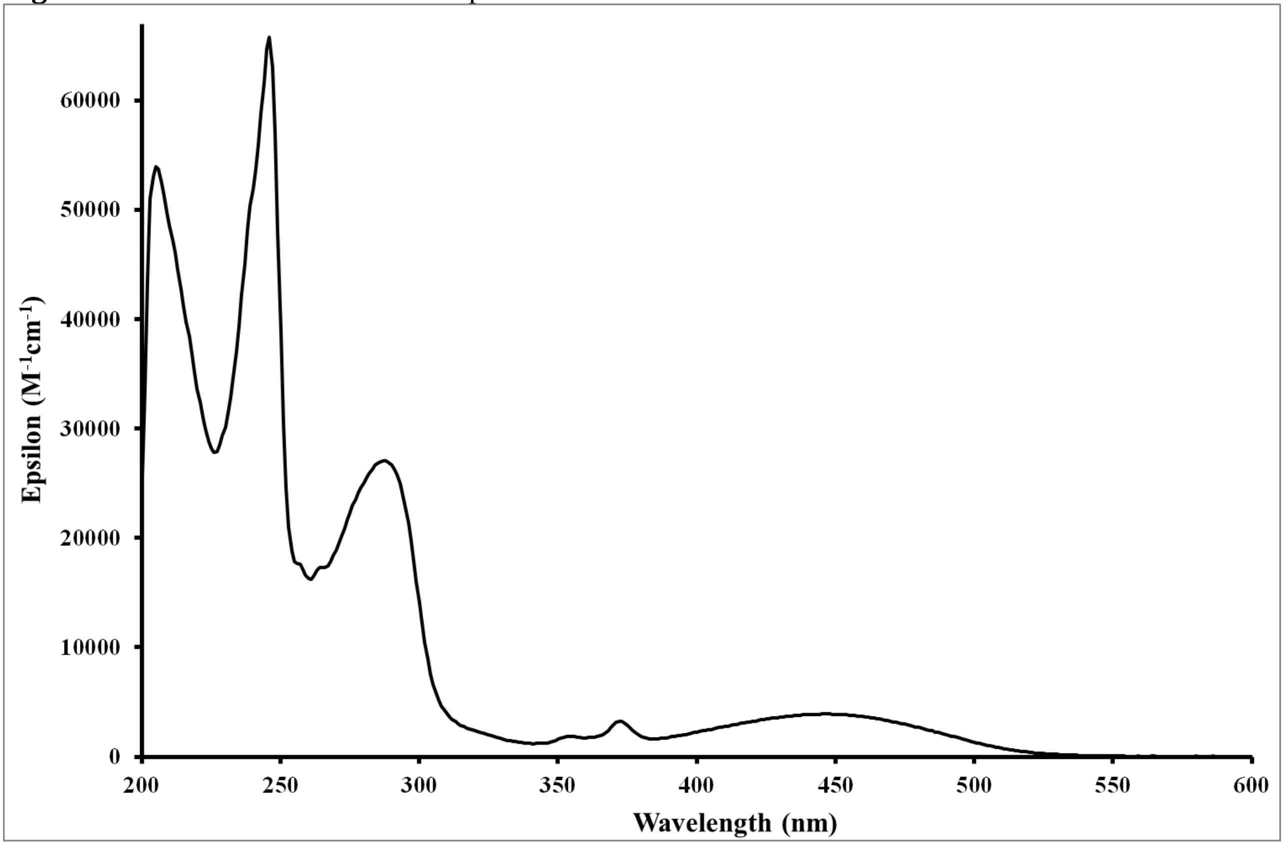

Figure S62. UV-Vis absorbance spectrum of MICAA recorded in isopropanole

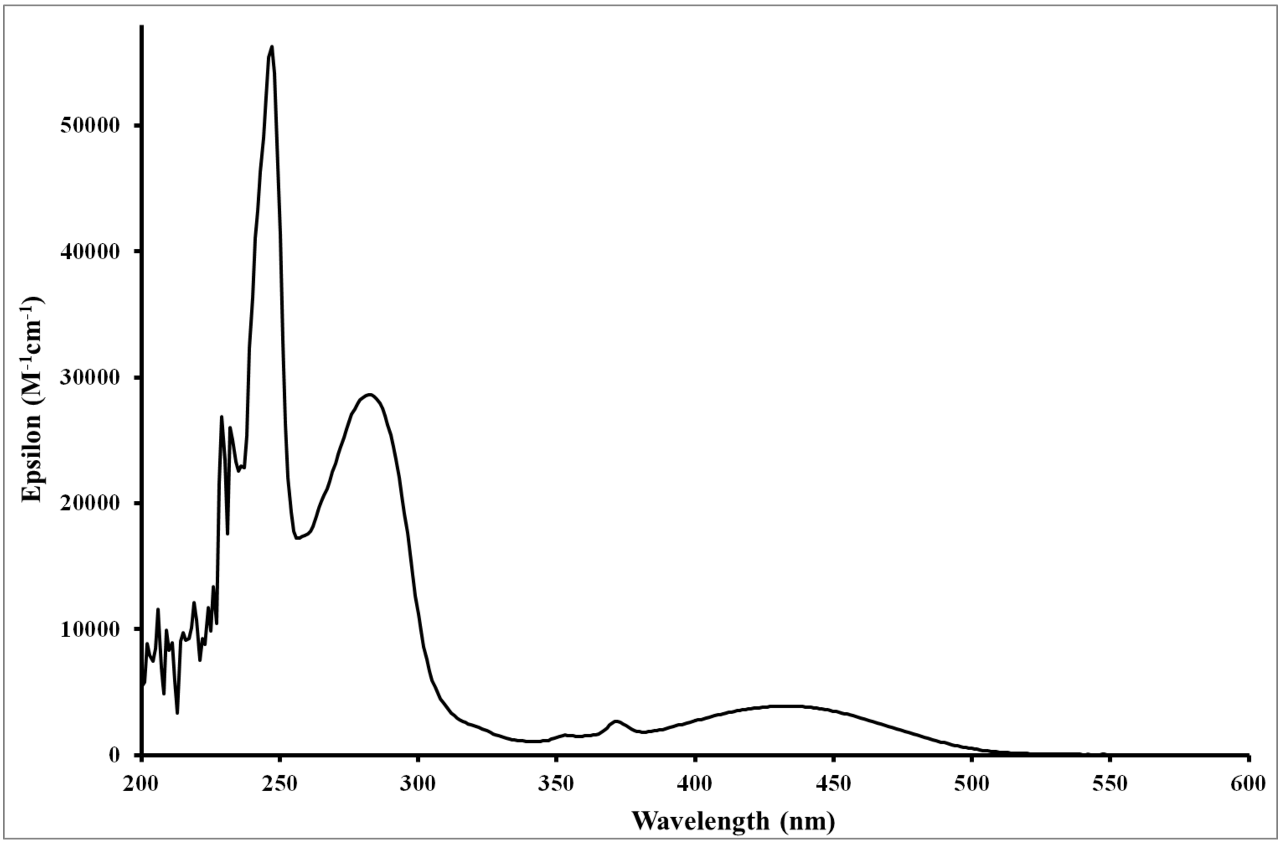

Figure S63. UV-Vis absorbance spectrum of MICAA recorded in chloroform

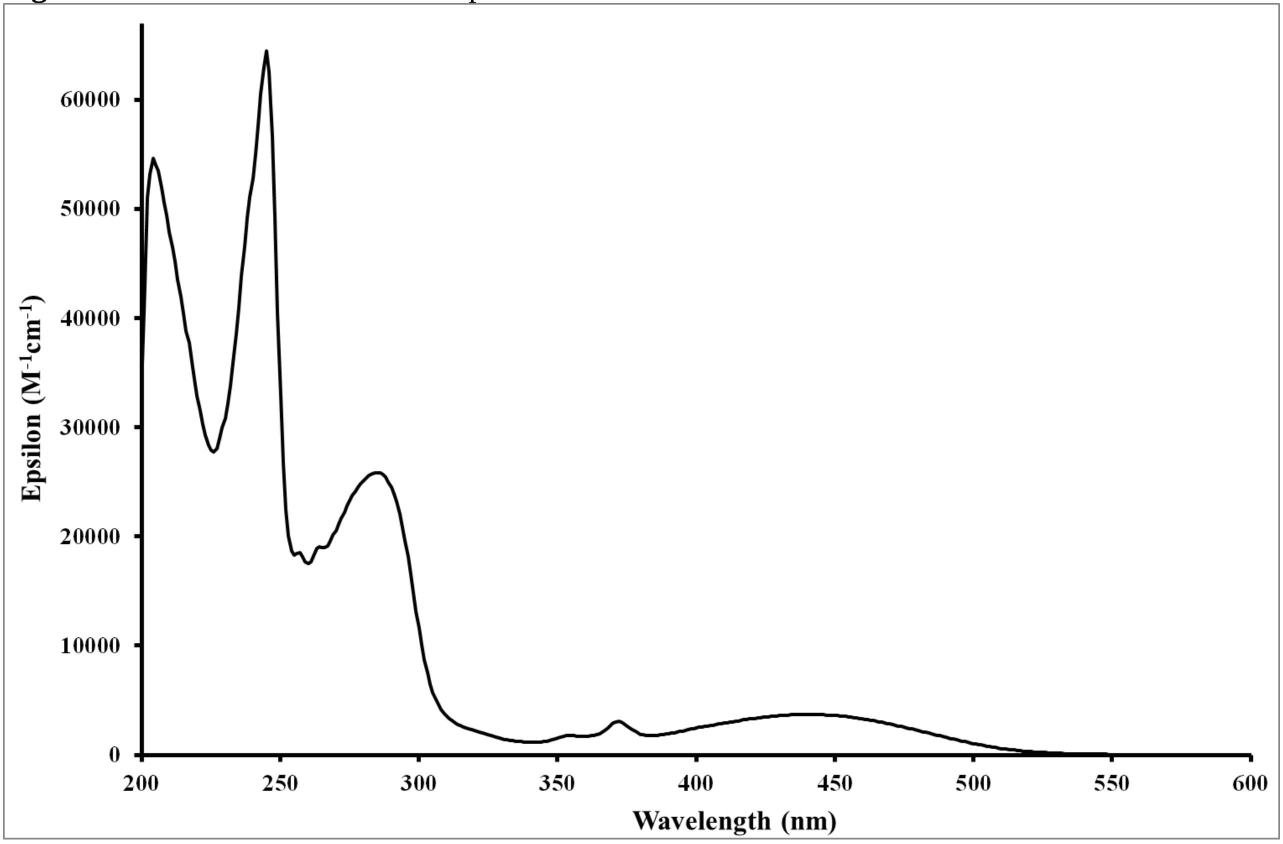

Figure S64. UV-Vis absorbance spectrum of MICAA recorded in methanol

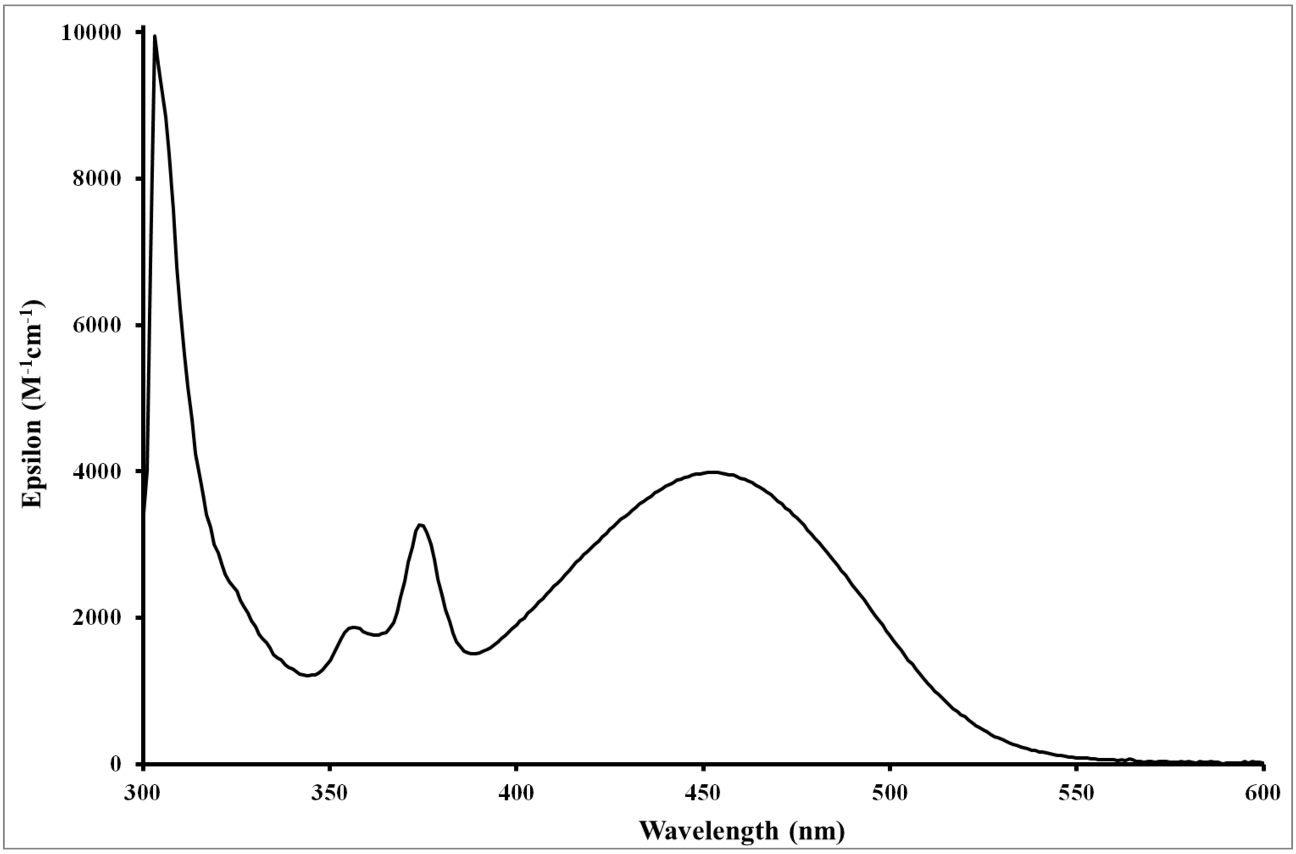

Figure S65. UV-Vis absorbance spectrum of MICAA recorded in pyridine

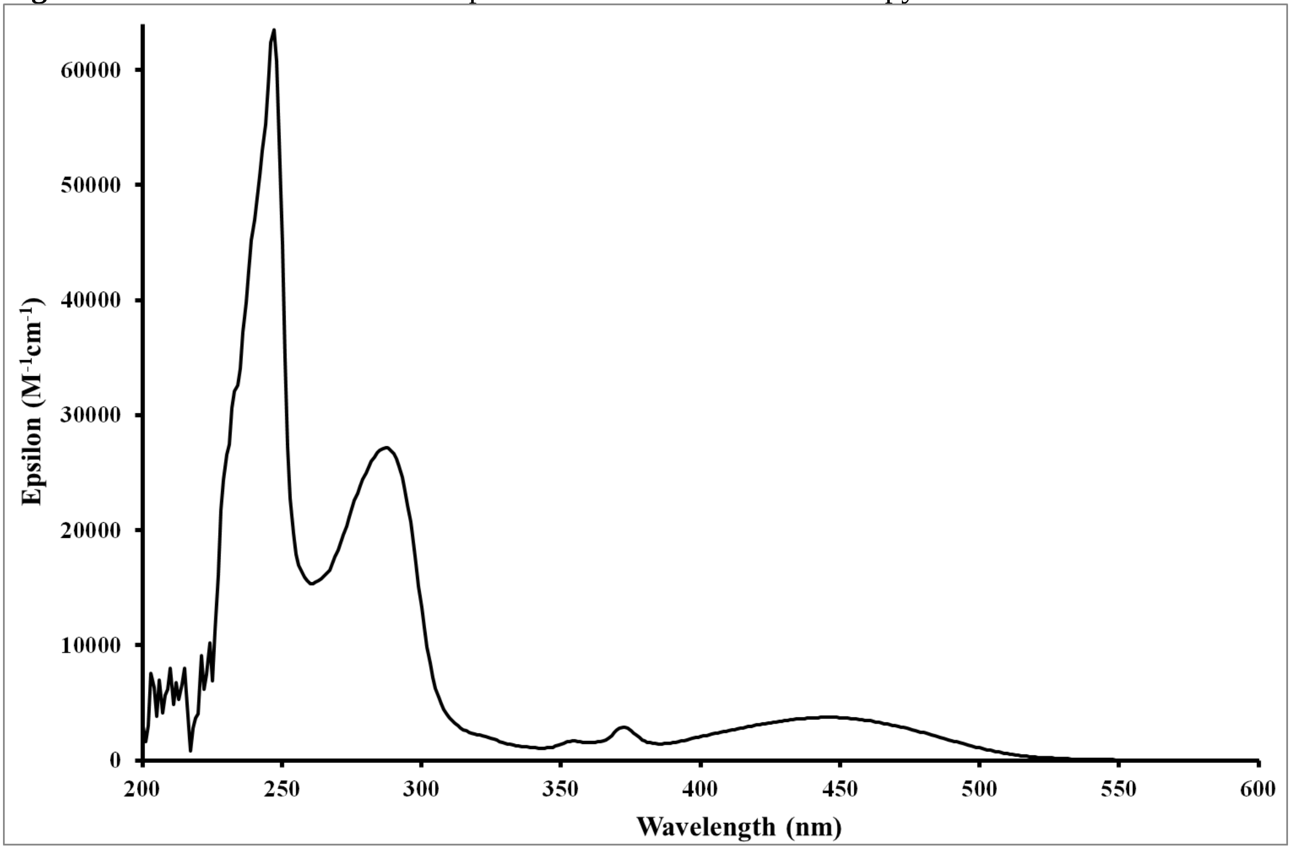

Figure S66. UV-Vis absorbance spectrum of MICAA recorded in tetrahydrofurane

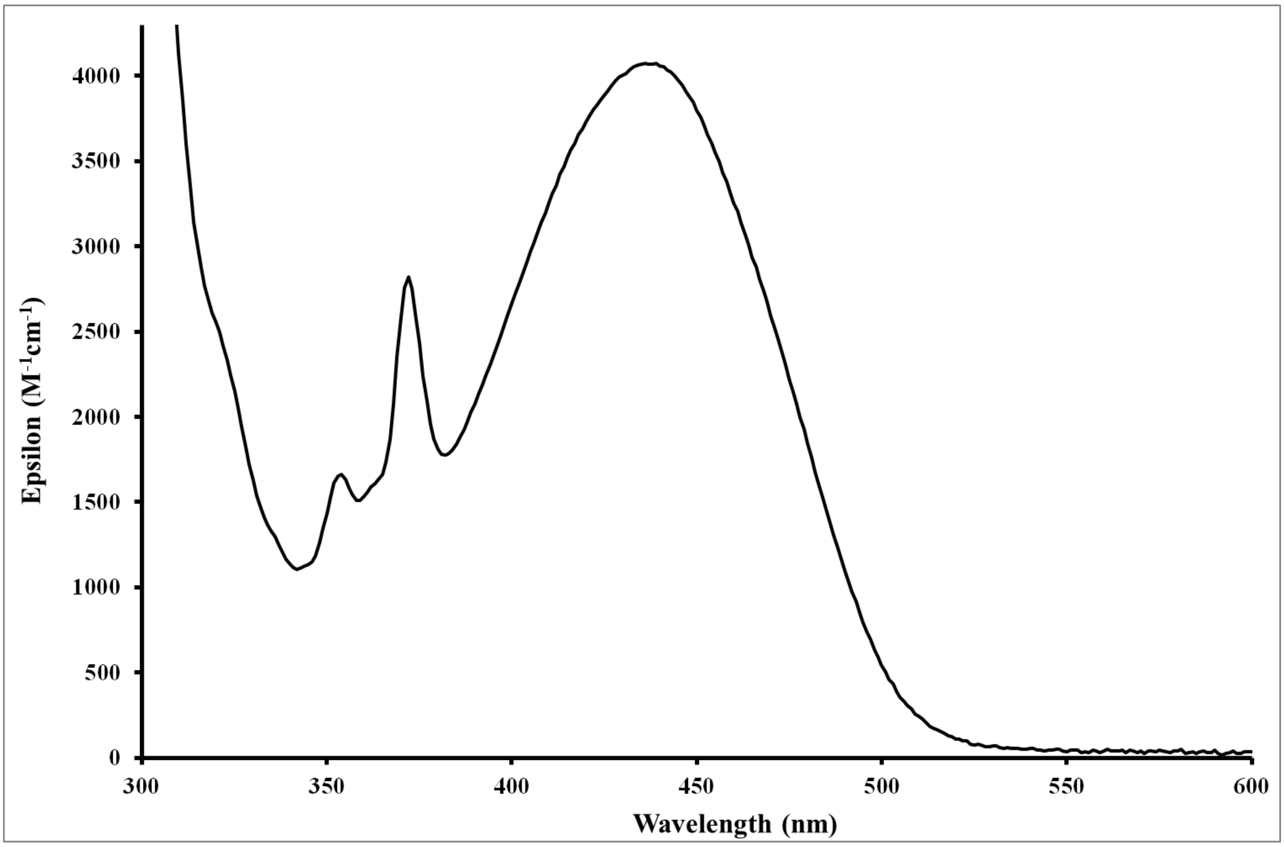

Figure S67. UV-Vis absorbance spectrum of MICAA recorded in toluene

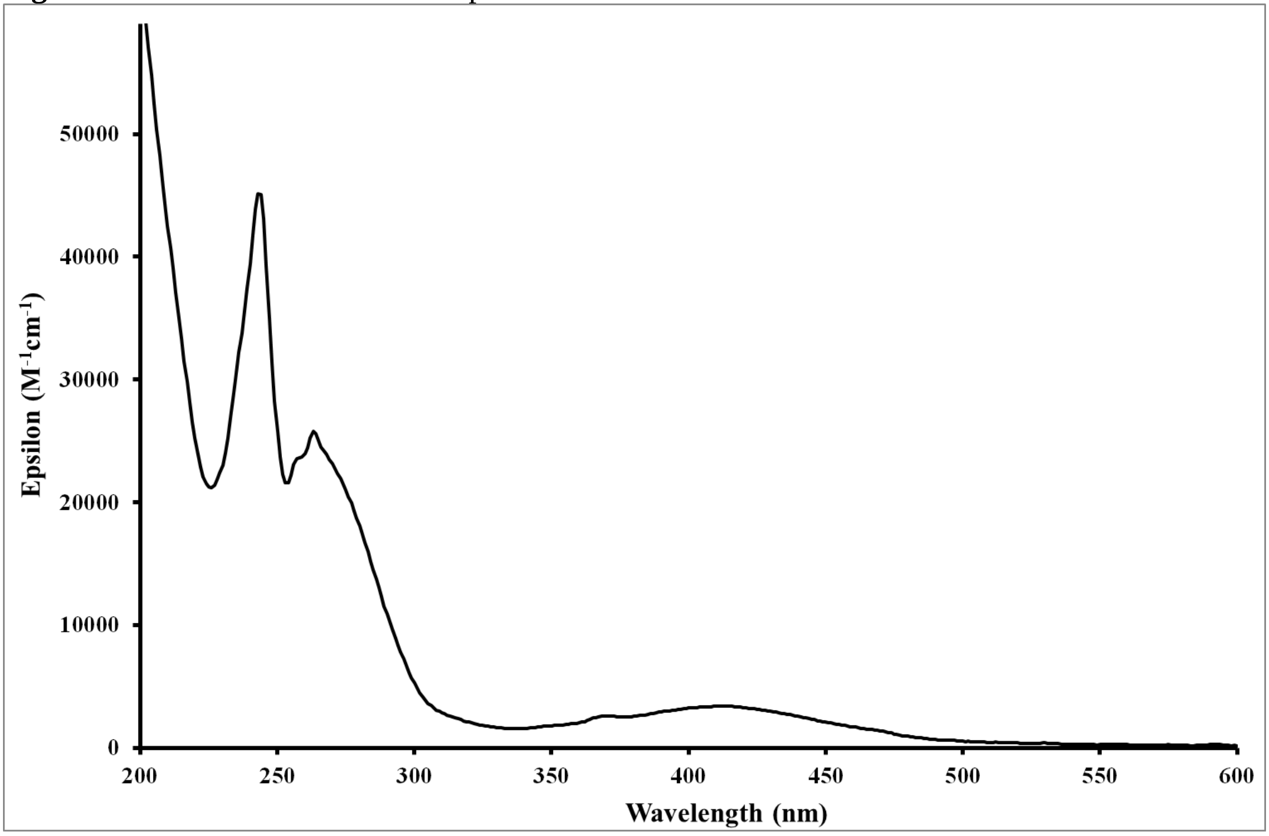

Figure S68. UV-Vis absorbance spectrum of MICAA recorded in water

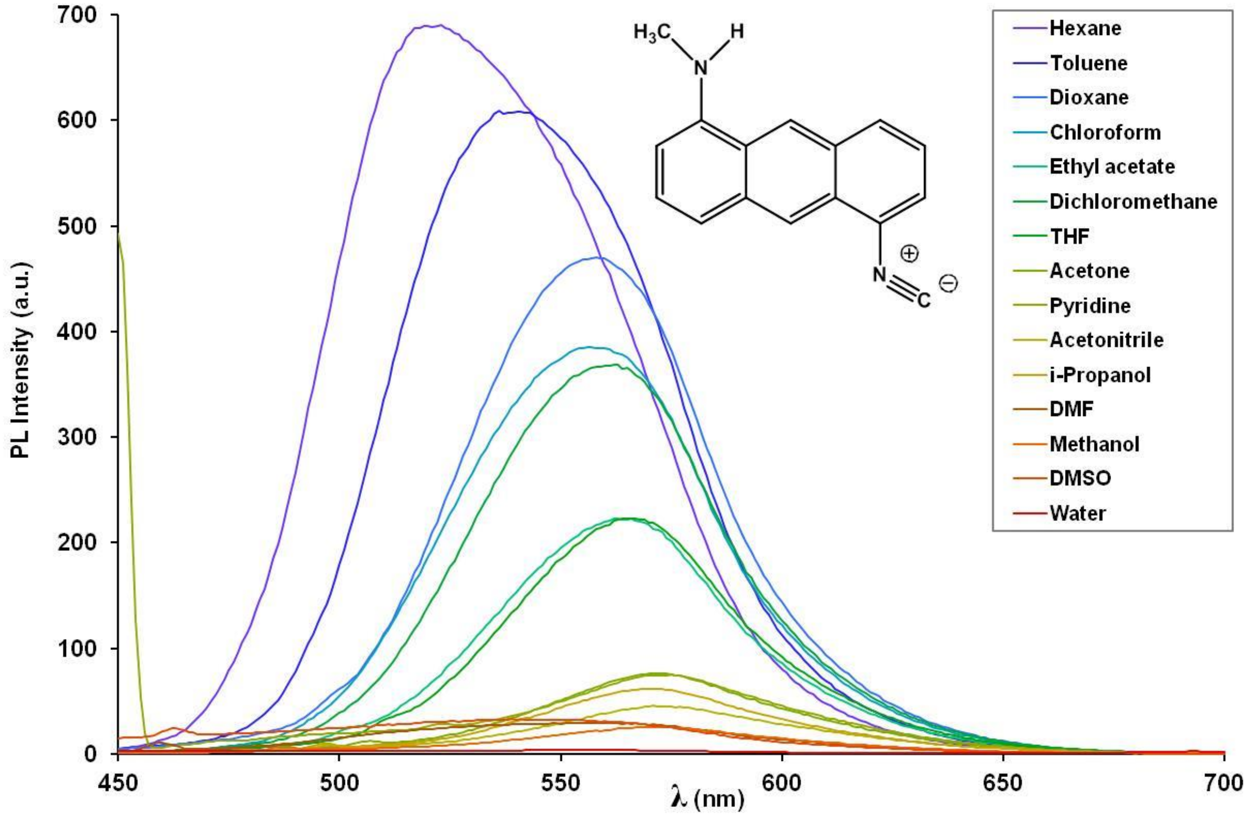

Figure S69. The emission spectra of 1-N-methylamino-5-isocyanoanthracene (MICAA) recorded in solvents of different polarity. (c= 5×10^{-5} M, T=20 ºC, V=3.00 ml).

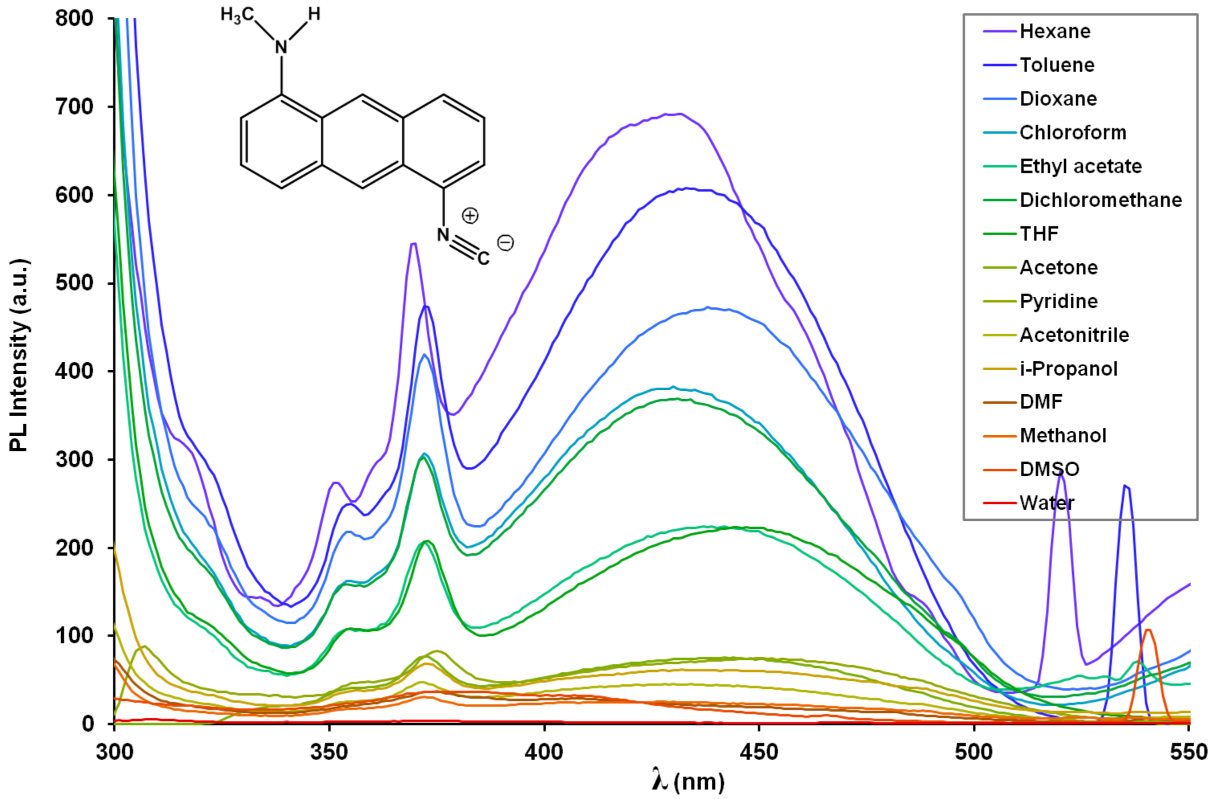

Figure S70. The excitation spectra of 1-N-methylamino-5-isocyanoanthracene (MICAA) recorded in solvents of different polarity. (c= 5×10^{-5} M, T=20 ºC, V=3.00 ml).

Chapter V. 1-N,N-dimethylamino-5-isocyanoanthracene (DIMICAA)

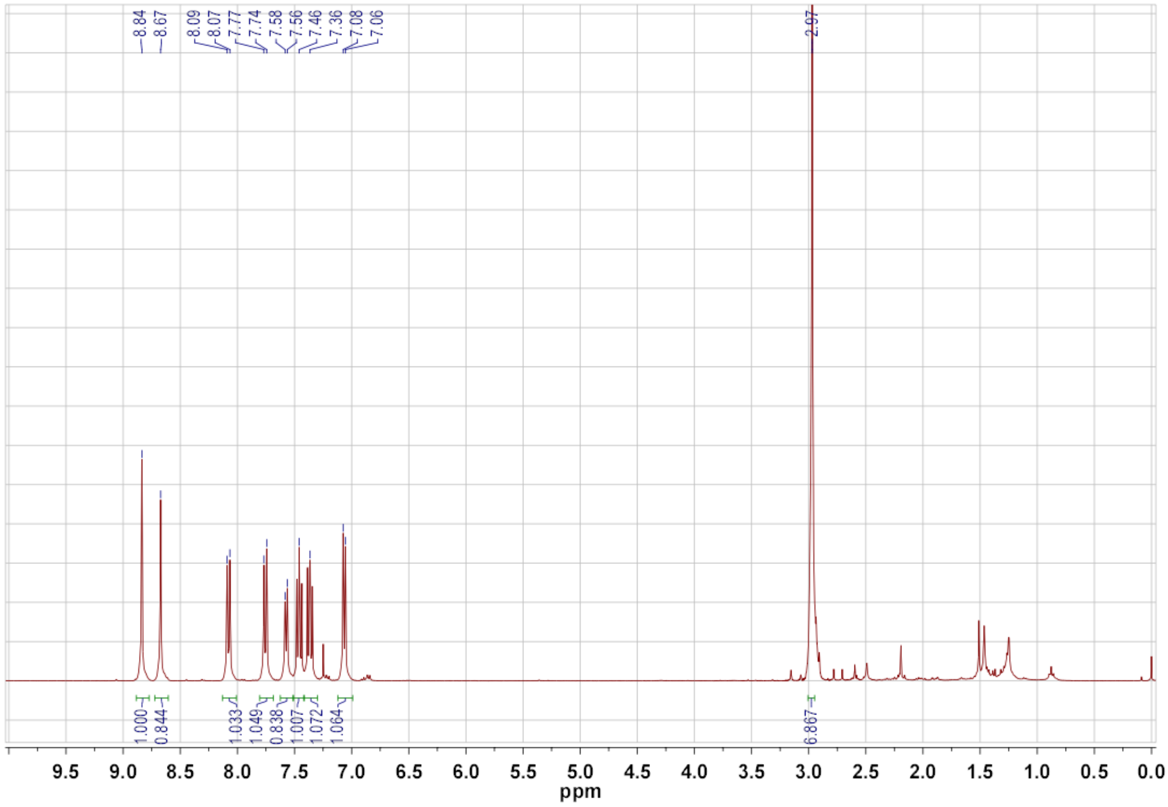

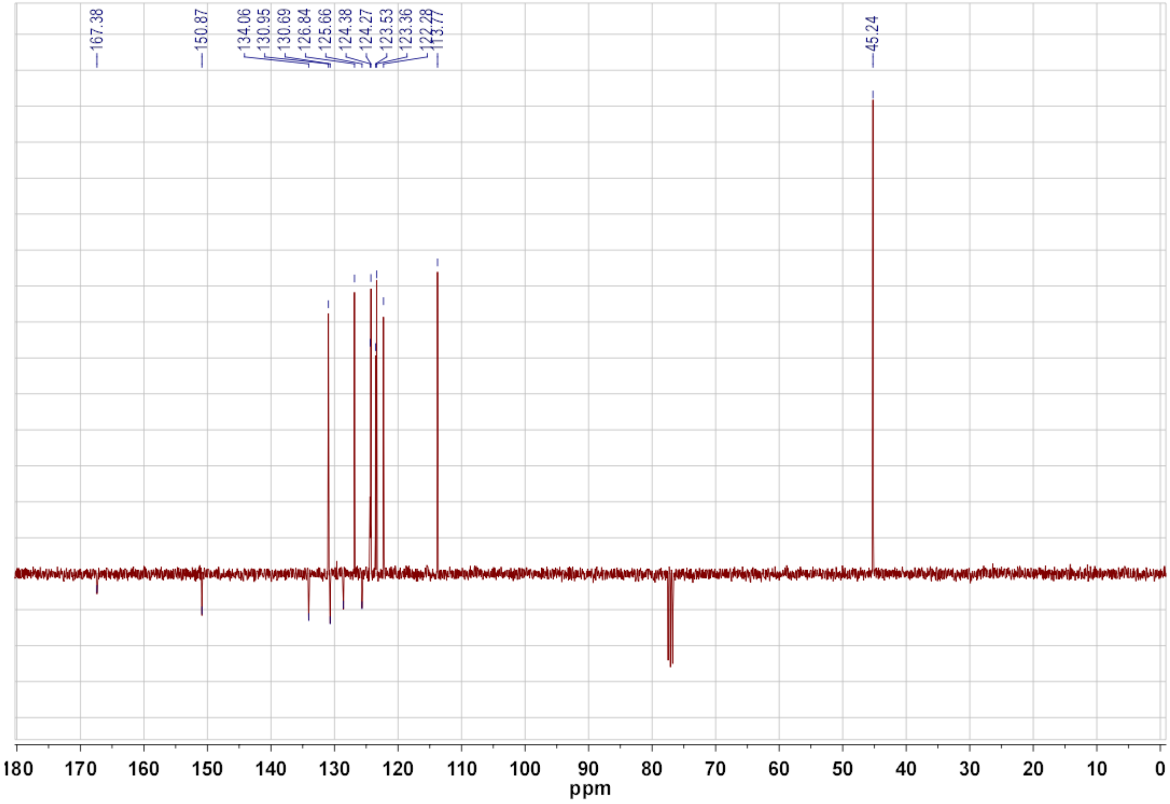

Figure S71. ^{1}H-NMR (top) and ^{13}C-NMR (bottom) spectra of 1-N,N-dimethylamino-5-isocyanoanthracene (DIMICAA) recorded at 20 °C in $CDCl_3$

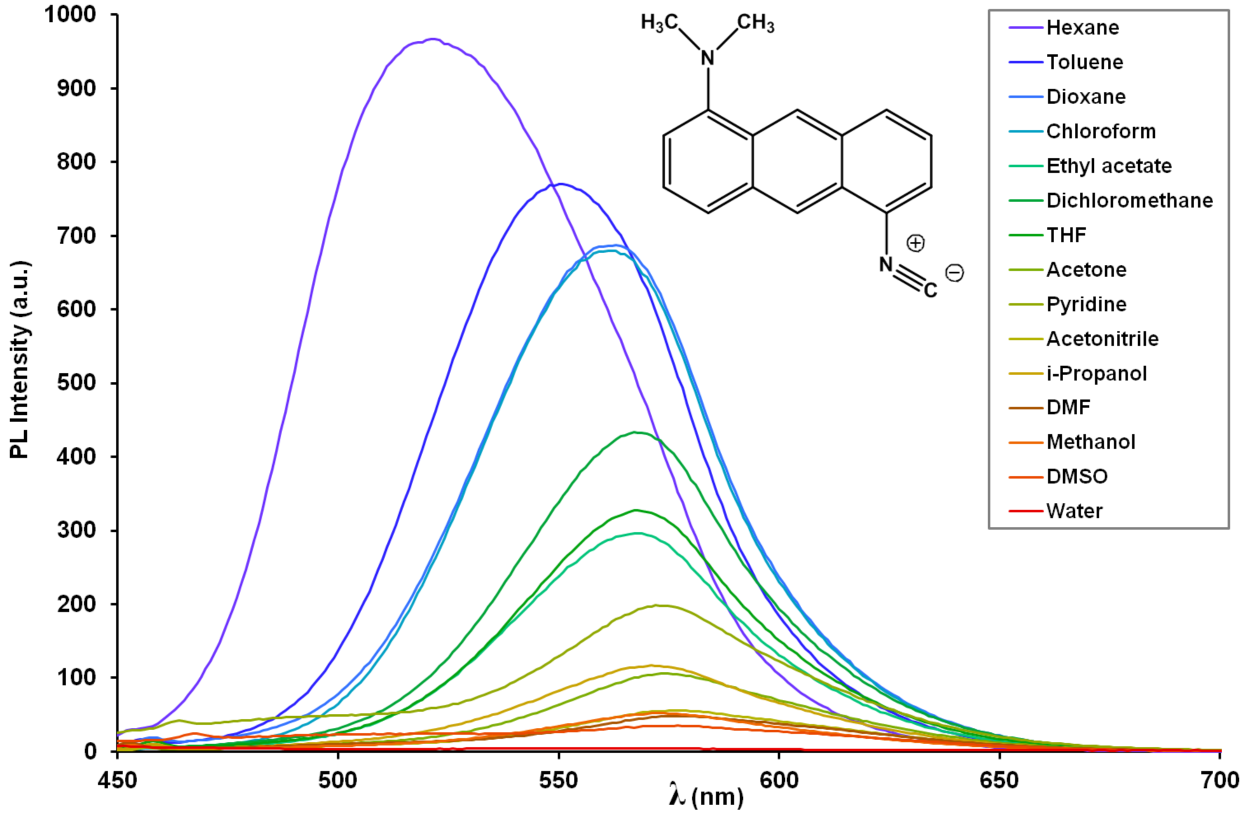

Figure S72. The emission spectra of 1-N,N-dimethylamino-5-isocyanoanthracene (DIMICAA) recorded in solvents of different polarity. (c= 5×10^{-5} M, T=20 ºC, V=3.00 ml).

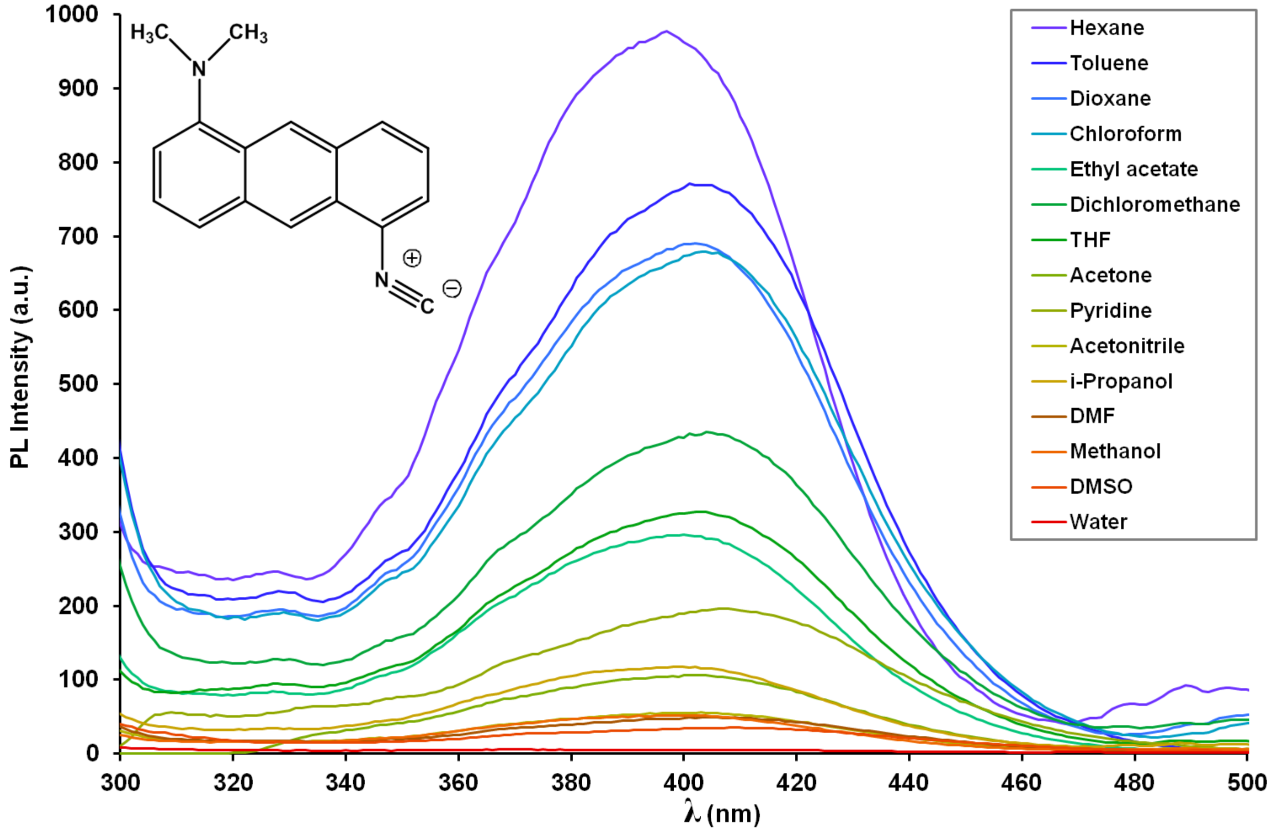

Figure S73. The excitation spectra of 1-N,N-dimethylamino-5-isocyanoanthracene (DIMICAA) recorded in solvents of different polarity. (c= 5×10^{-5} M, T=20 ºC, V=3.00 ml).

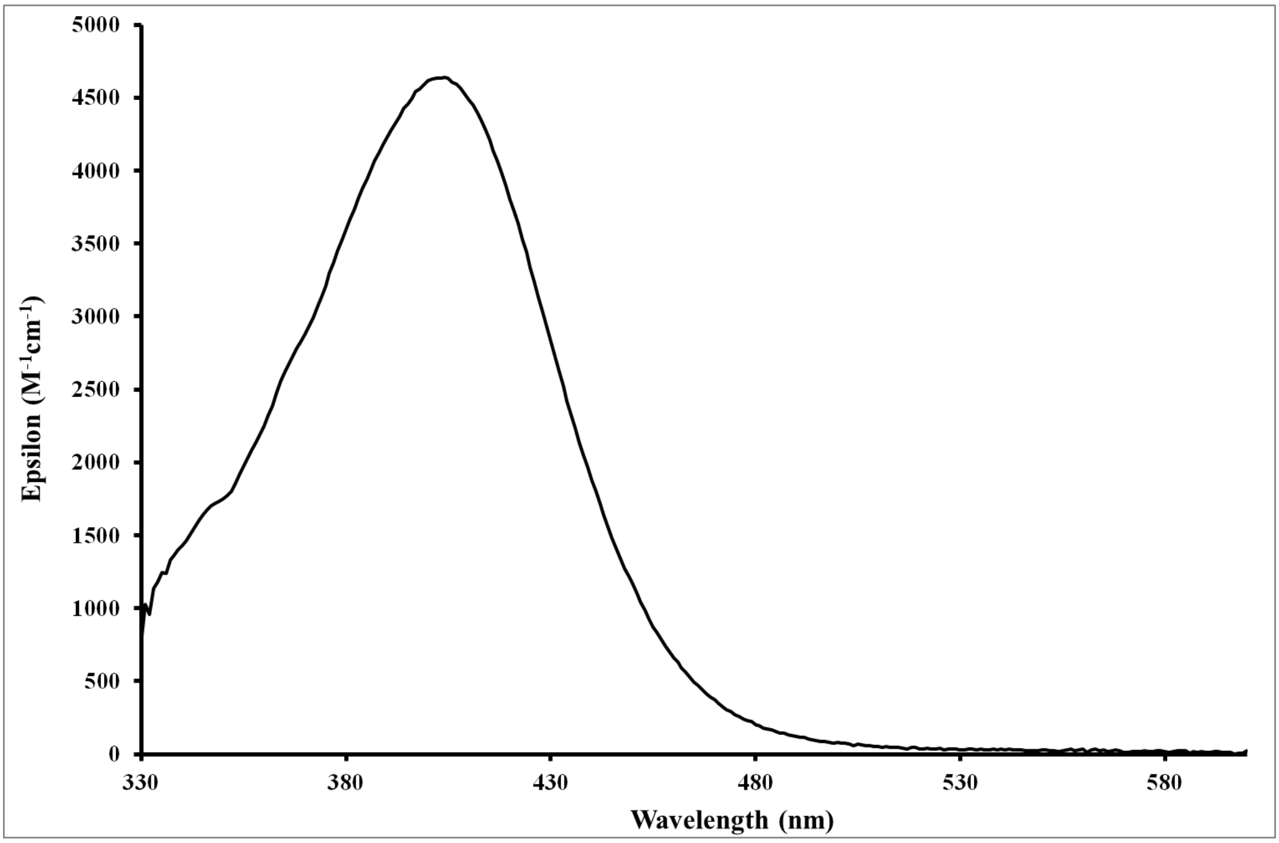

Figure S74. The UV-vis spectrum of (DIMICAA) recorded in acetone

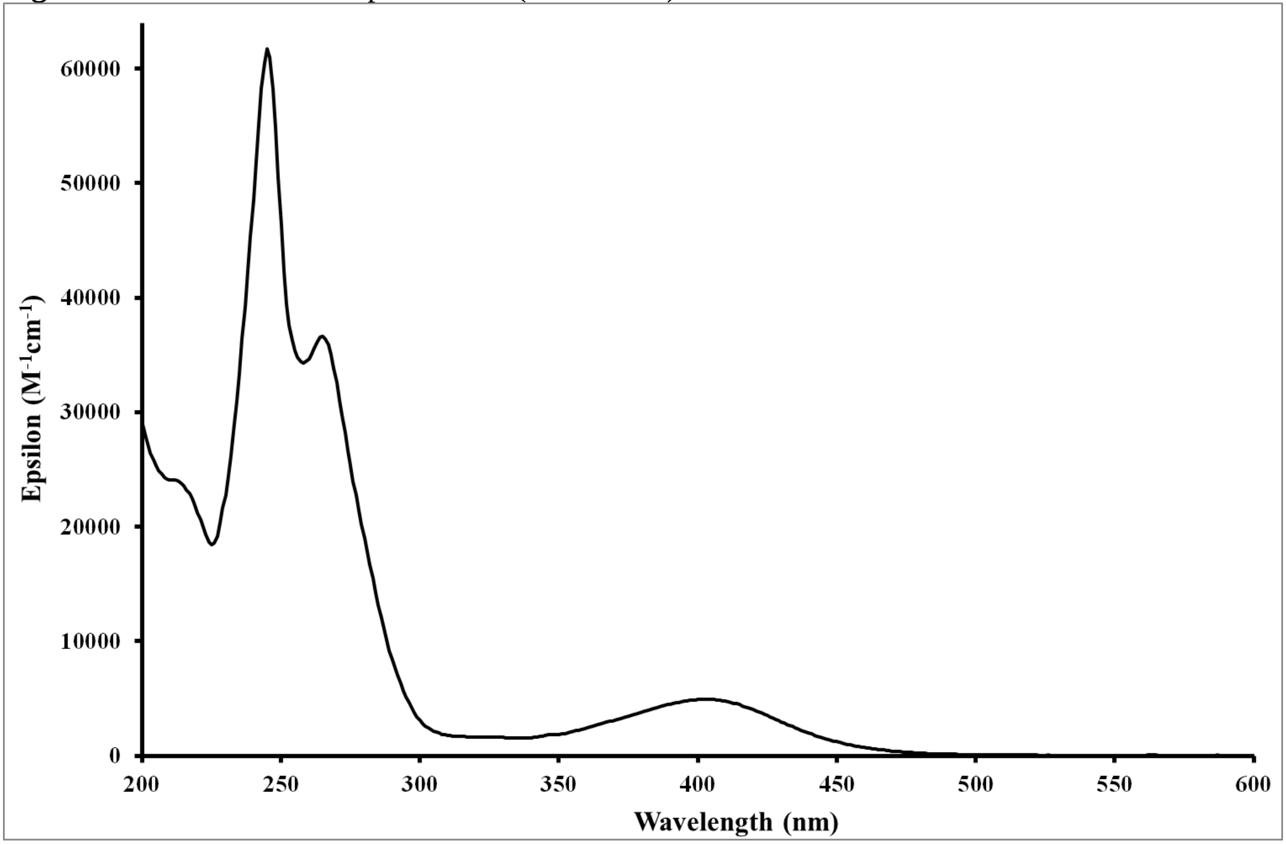

Figure S75. The UV-vis spectrum of (DIMICAA) recorded in acetonitrile

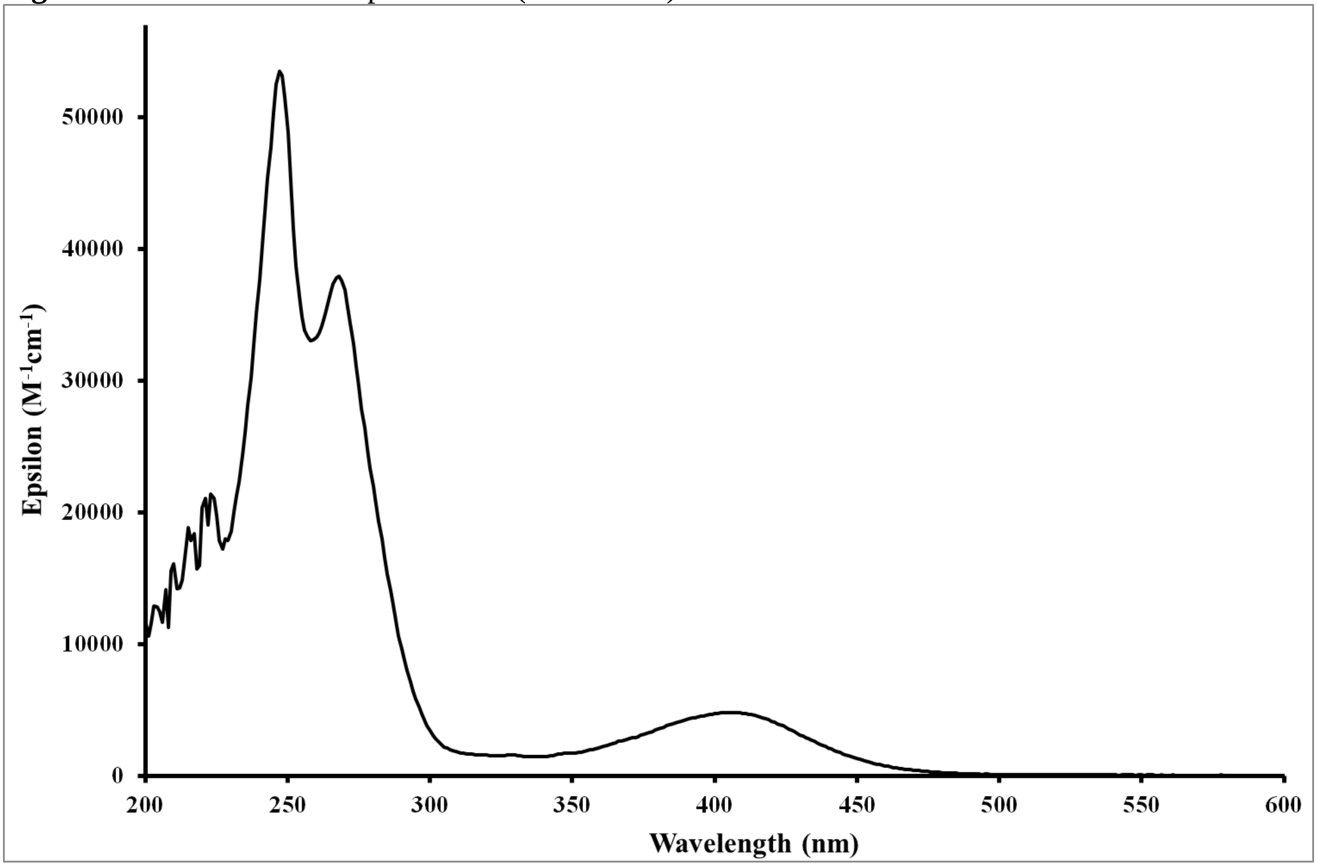

Figure S76. The UV-vis spectrum of (DIMICAA) recorded in methylene-chloride

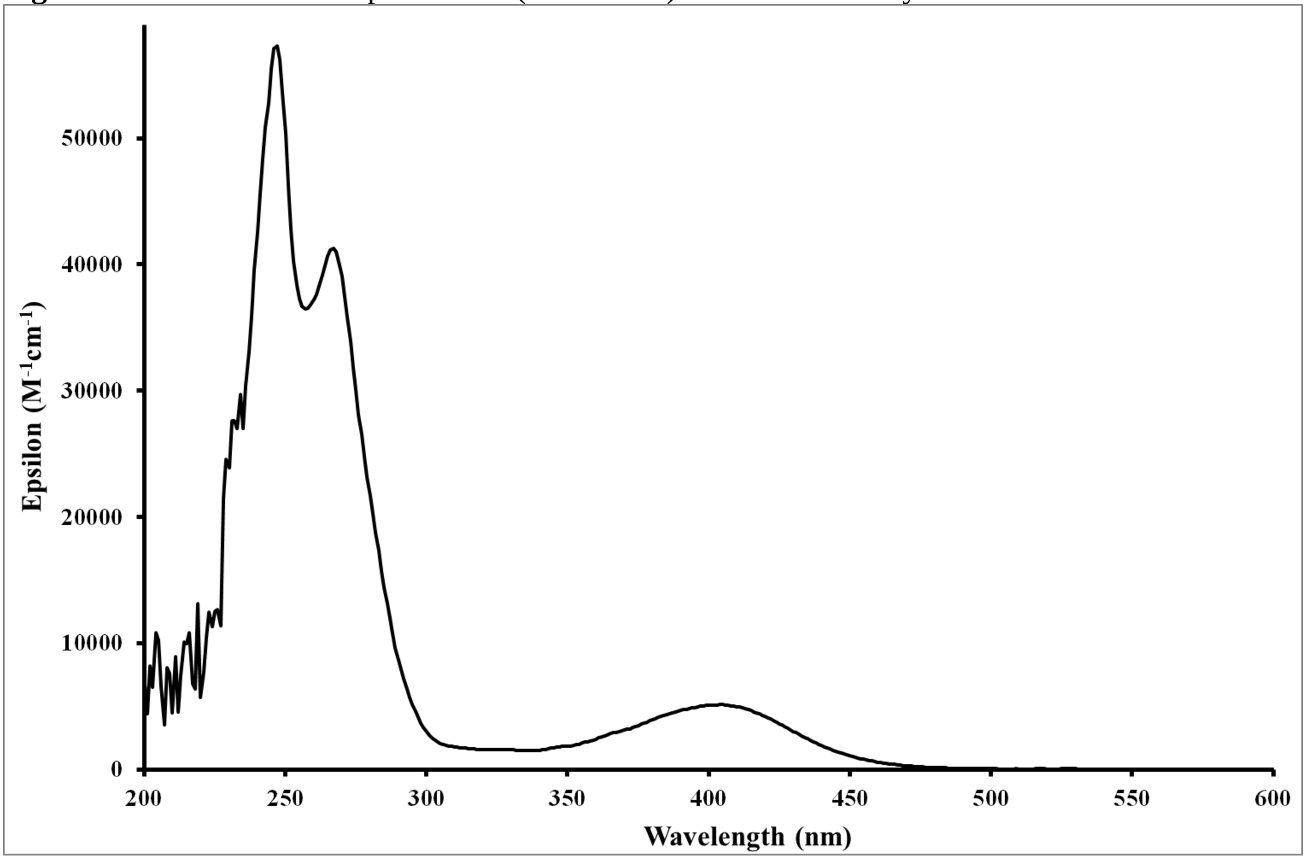

Figure S77. Dioxane The UV-vis spectrum of (DIMICAA) recorded in dioxane

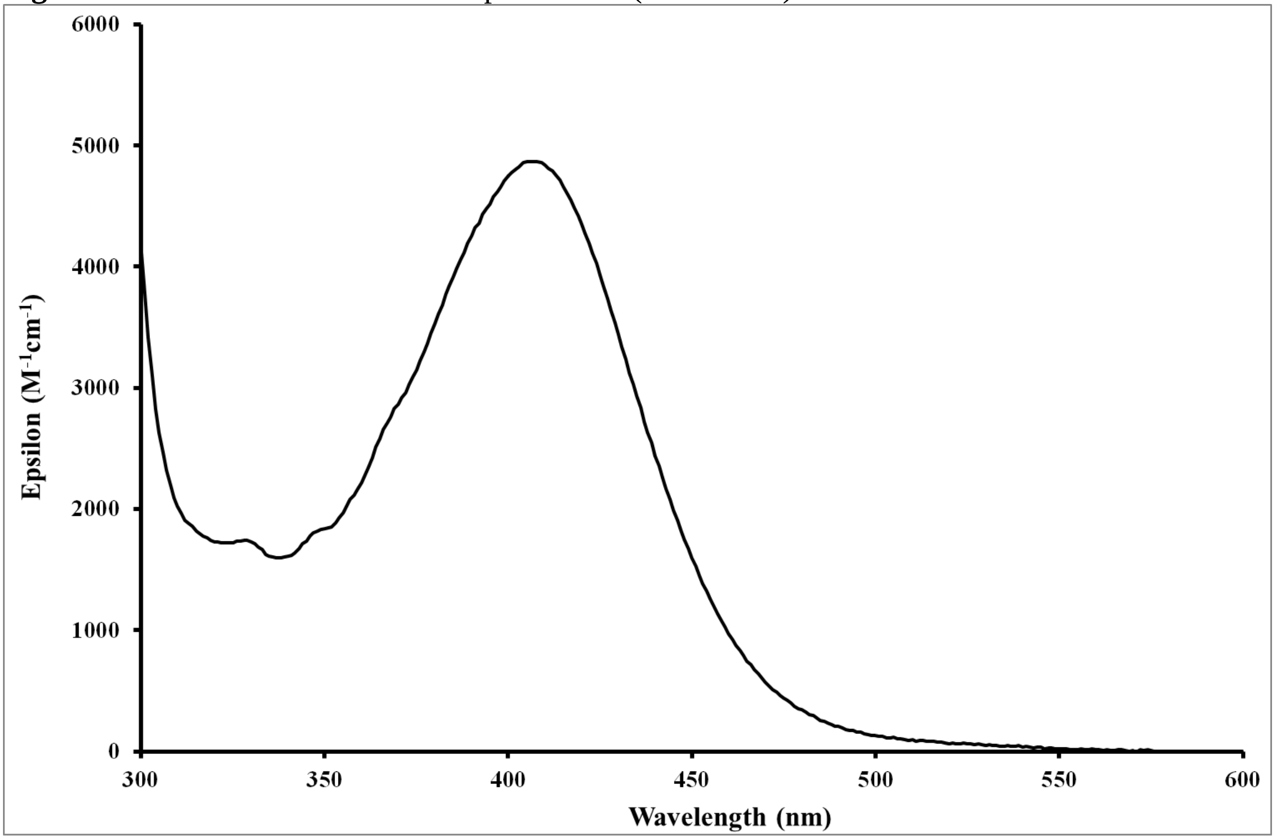

Figure S78. The UV-vis spectrum of (DIMICAA) recorded in DMF

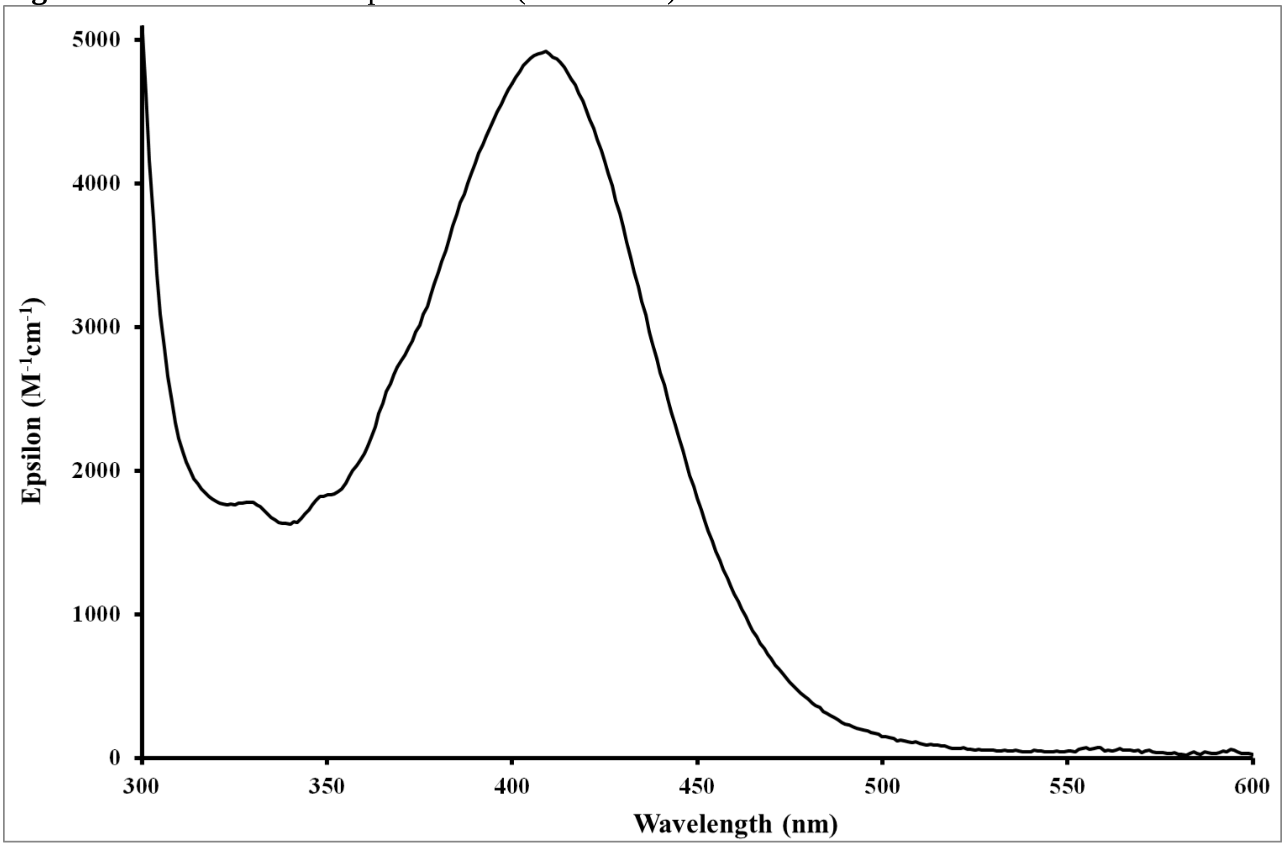

Figure S79. The UV-vis spectrum of (DIMICAA) recorded in DMSO

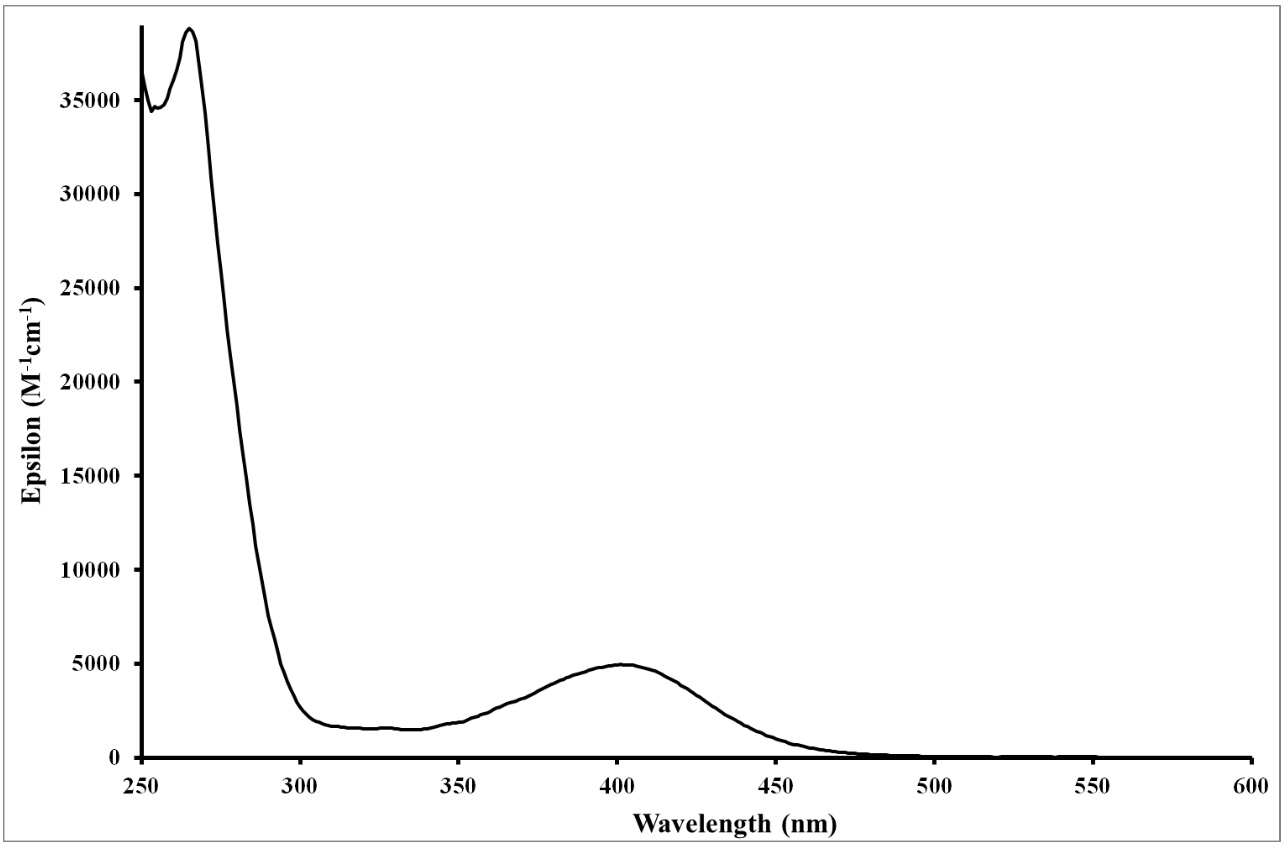

Figure S80. The UV-vis spectrum of (DIMICAA) recorded in EtOAc

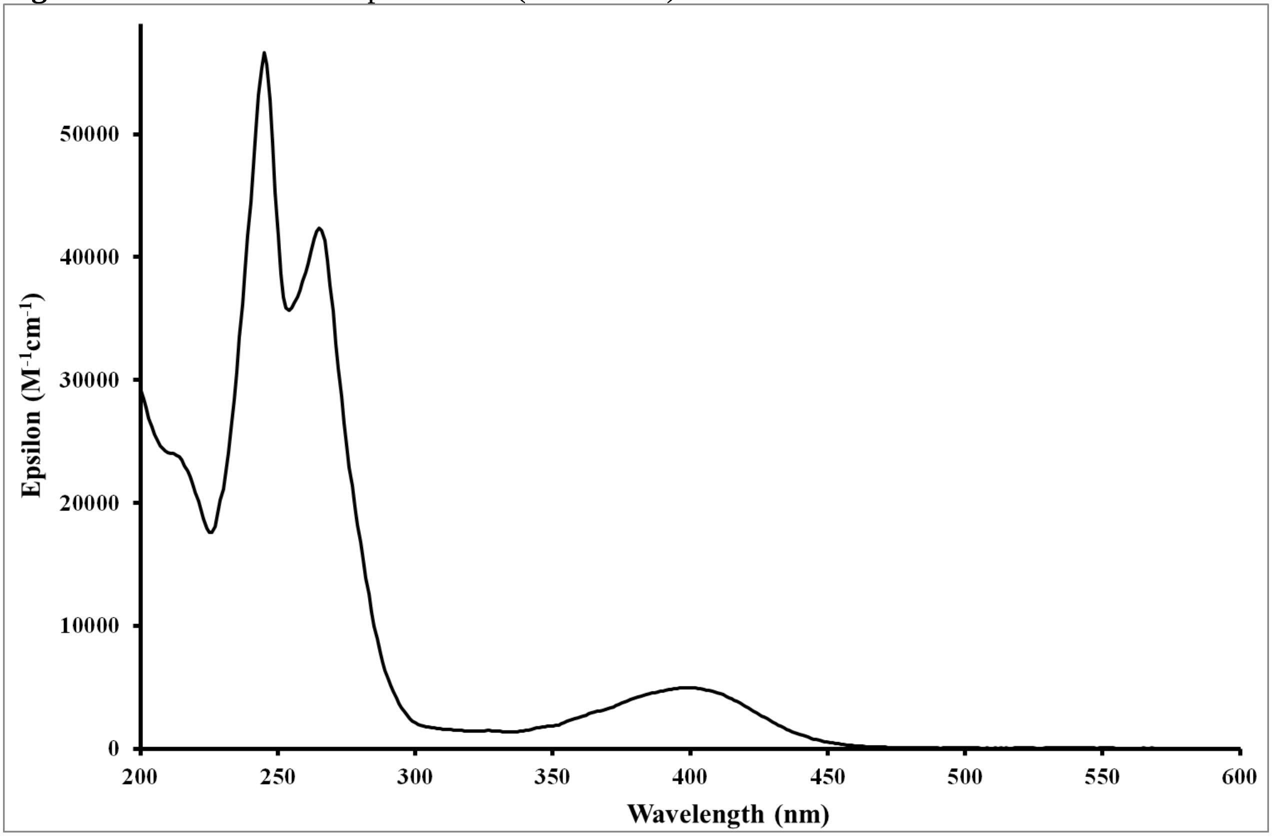

Figure S81. The UV-vis spectrum of (DIMICAA) recorded in hexane

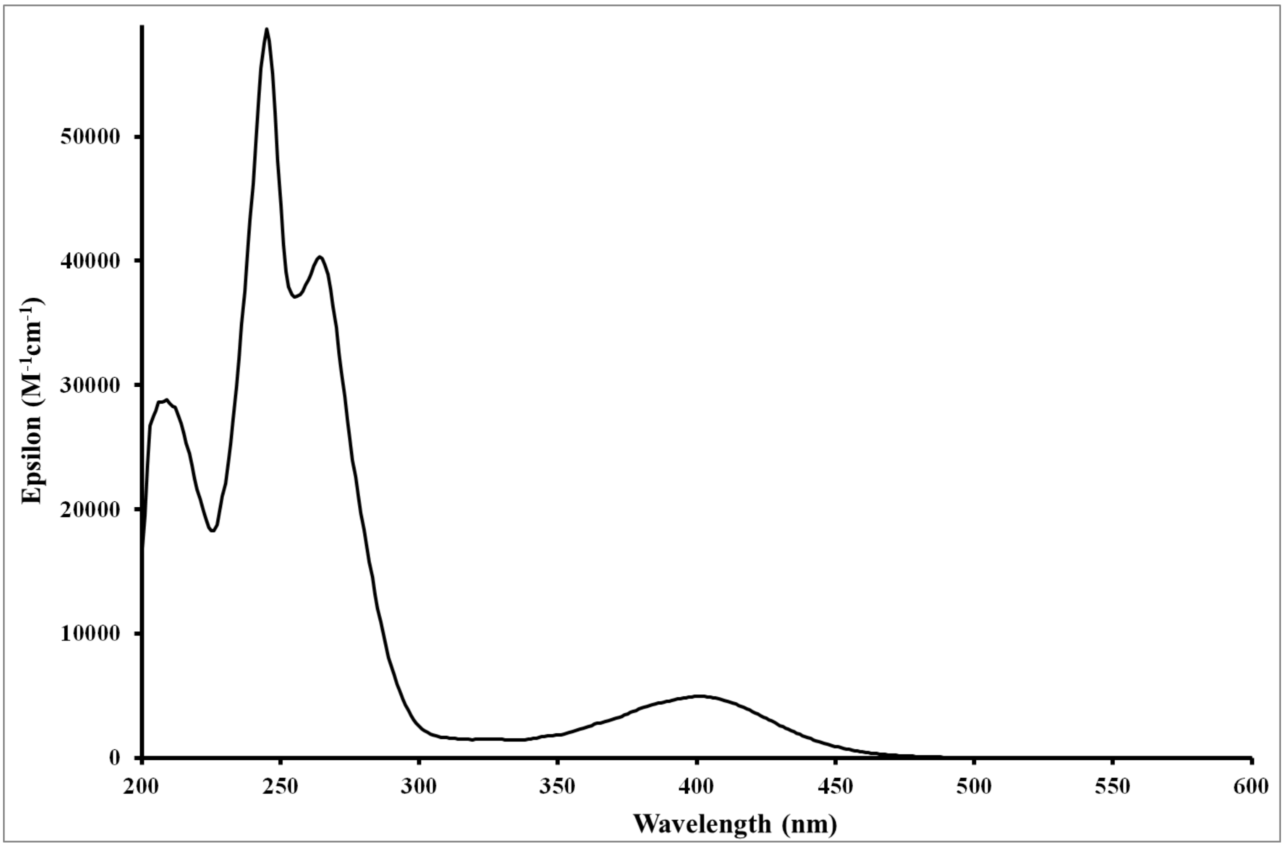

Figure S82. The UV-vis spectrum of (DIMICAA) recorded in isopropanol

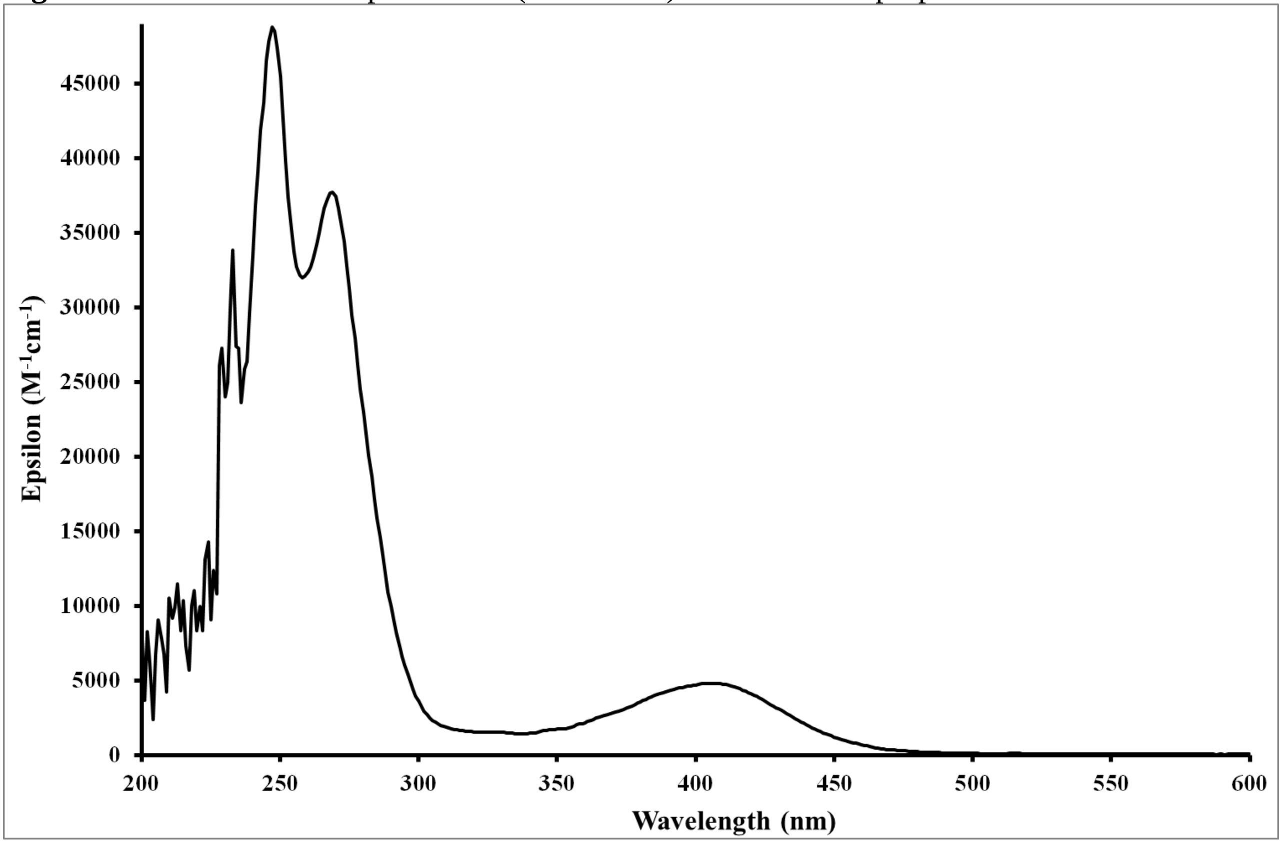

Figure S83. The UV-vis spectrum of (DIMICAA) recorded in choloroform

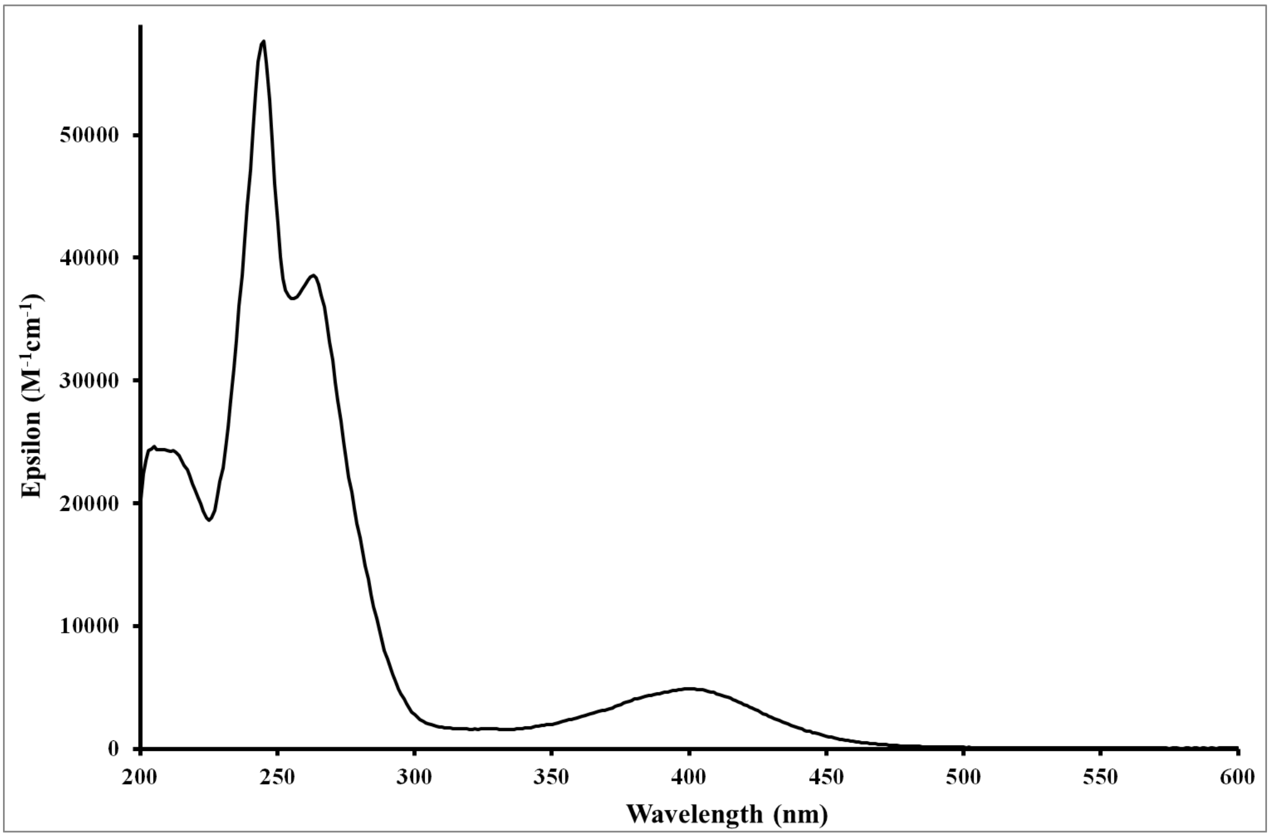

Figure S84. The UV-vis spectrum of (DIMICAA) recorded in methanol

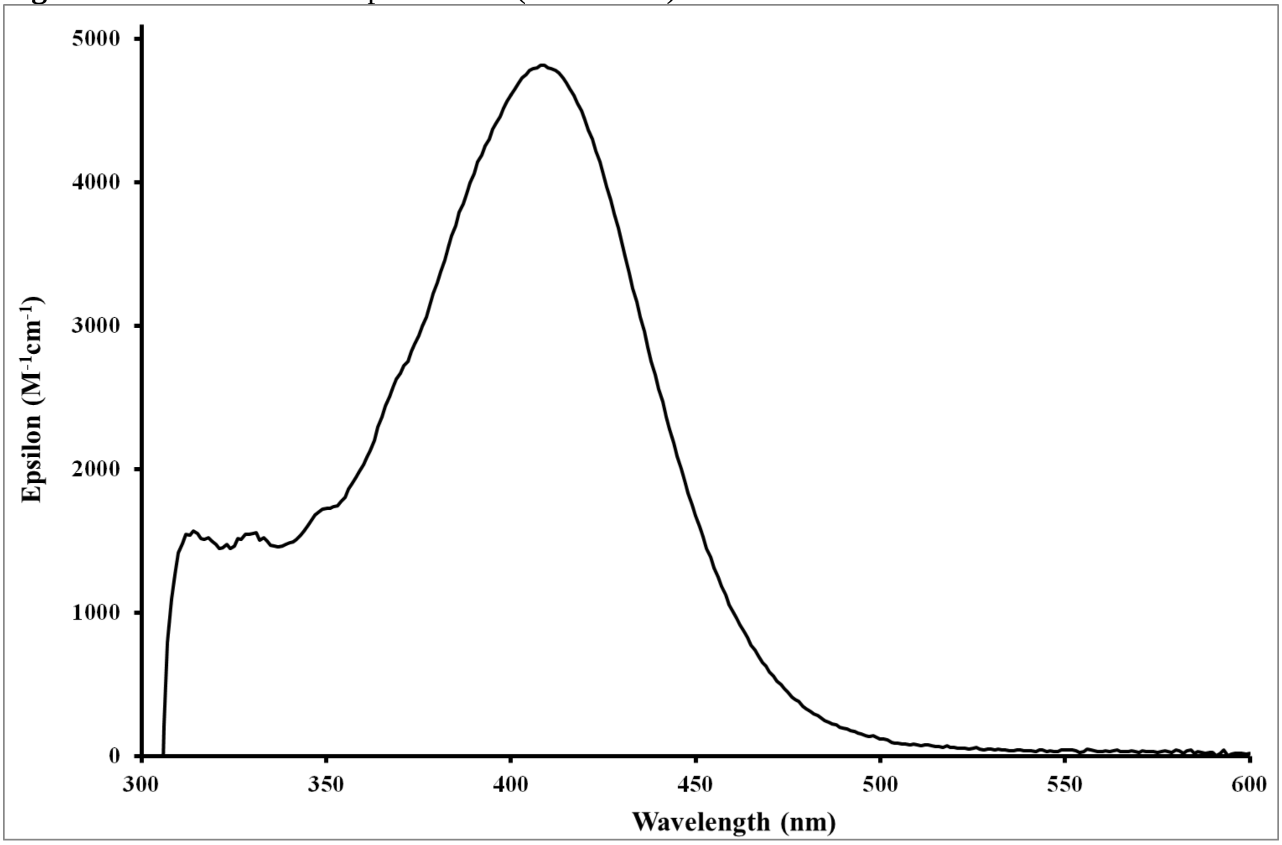

Figure S85. The UV-vis spectrum of (DIMICAA) recorded in pyridine

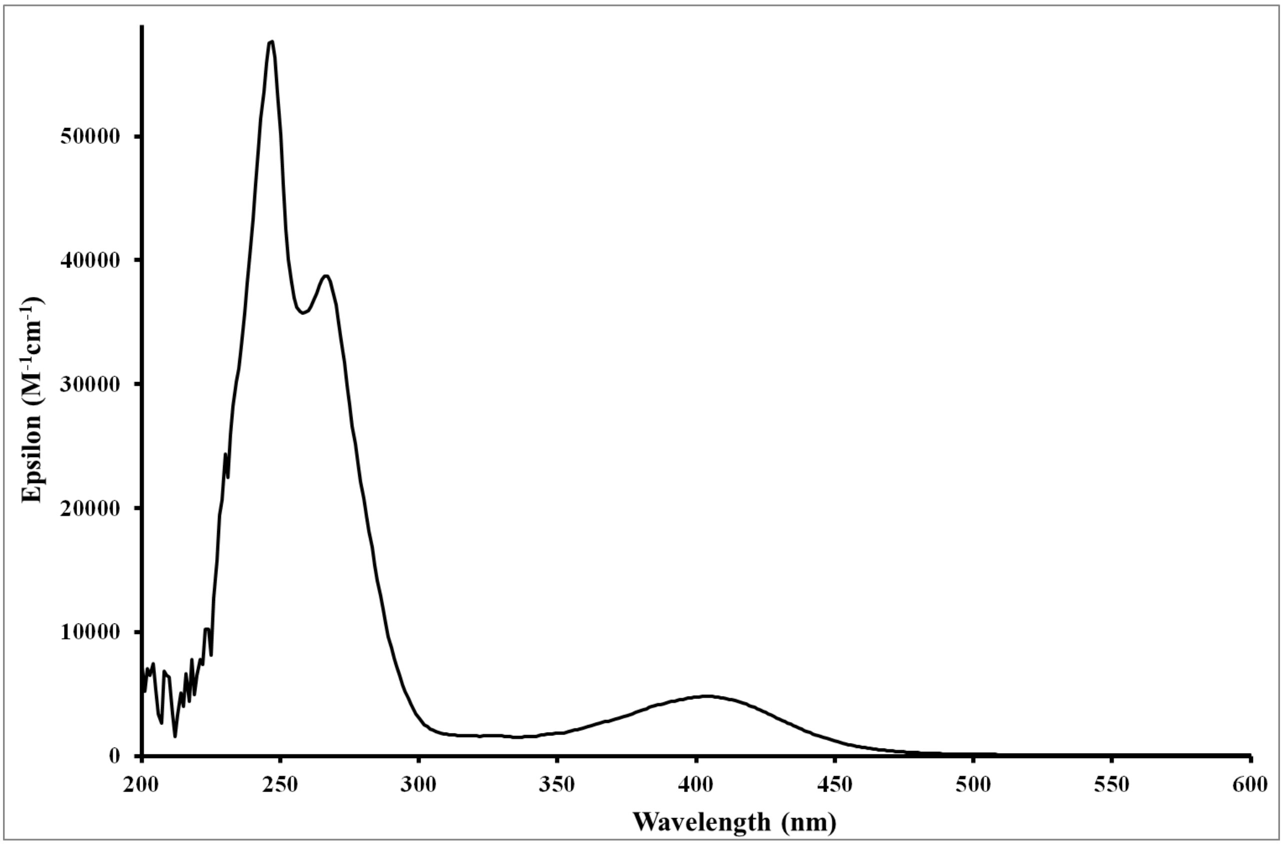
Epsilon (M-1cm-1)
50000
40000
30000
20000
10000
0
200
250
300
350
400
450
500
550
600
Wavelength (nm)

Figure S86. The UV-vis spectrum of (DIMICAA) recorded in tetrahydrofurane

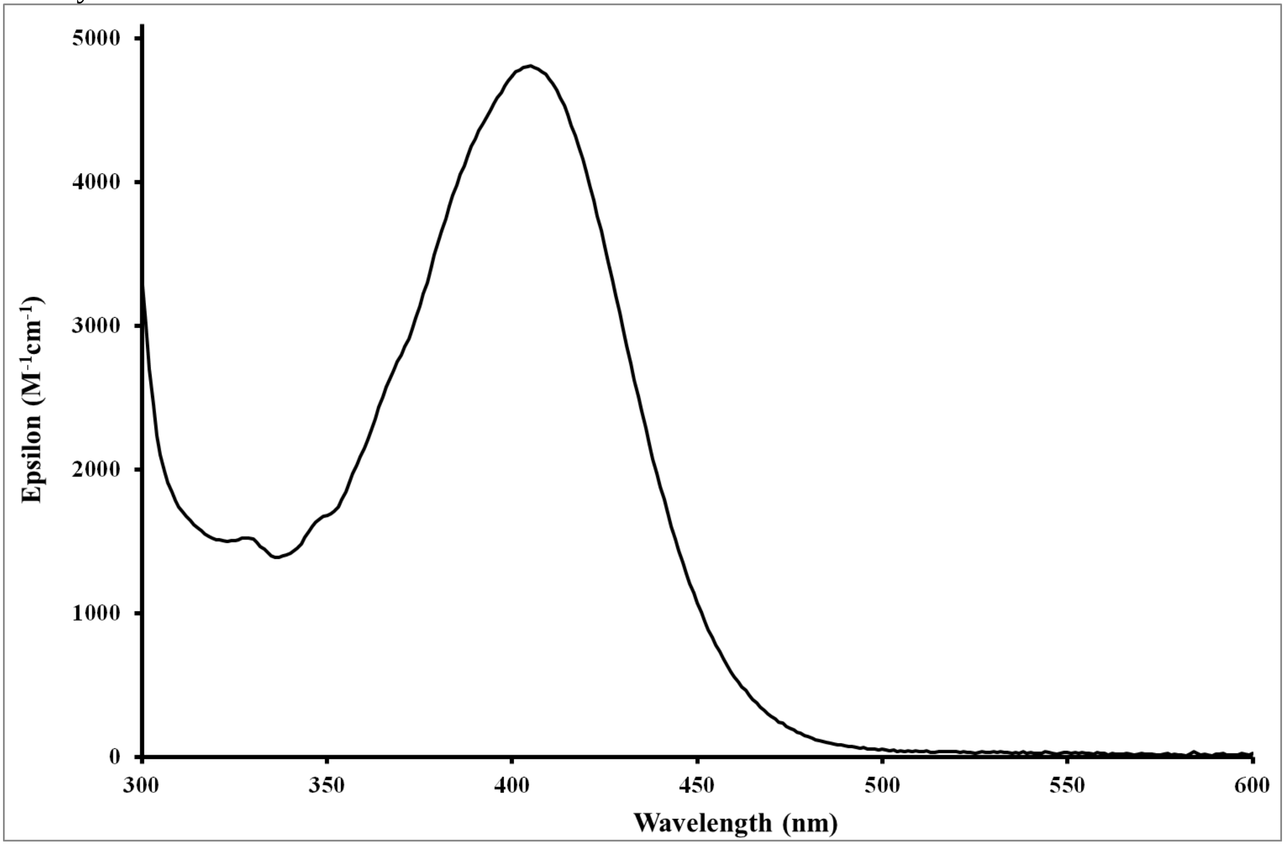

Figure S87. The UV-vis spectrum of (DIMICAA) recorded in toluene

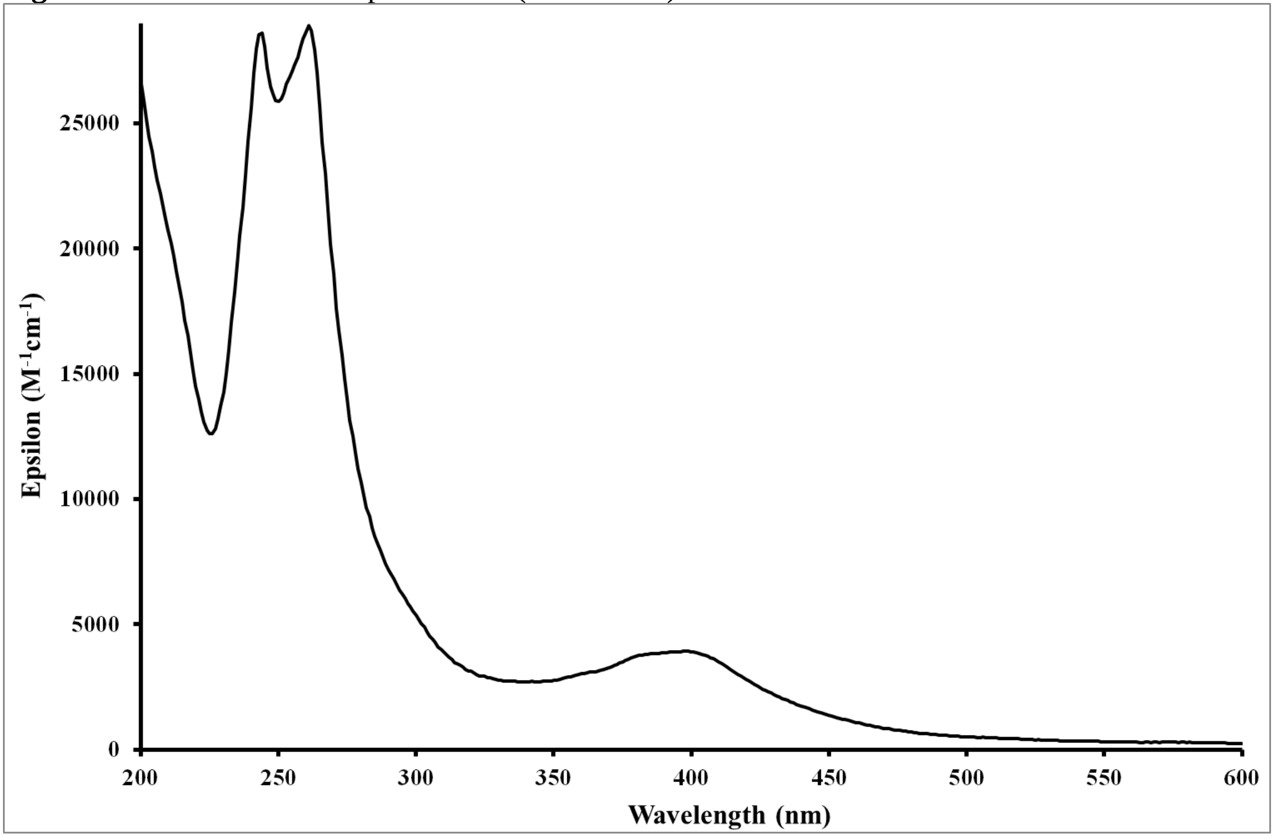

Figure S88. The UV-vis spectrum of (DIMICAA) recorded in water

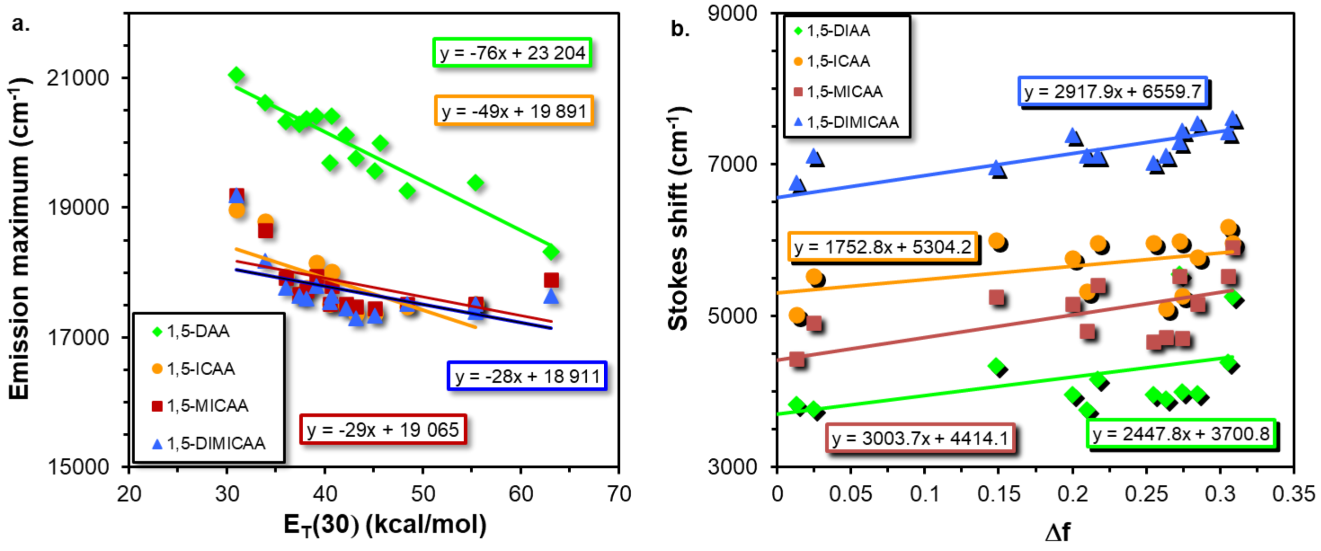

Figure S89. Variation of the fluorescence emission maximum with the empirical solvent polarity parameter $E_T(30)$ (**a**) and the Lippert-Mataga (LM) (**b**) plots for the 1,5-disubstituted anthracene dyes.
